# The Antioxidant Properties of Mushroom Polysaccharides can Potentially Mitigate Oxidative Stress, Beta-Cell Dysfunction and Insulin Resistance

**DOI:** 10.3389/fphar.2022.874474

**Published:** 2022-05-05

**Authors:** Karuppusamy Arunachalam, Puthanpura Sasidharan Sreeja, Xuefei Yang

**Affiliations:** ^1^ Key Laboratory of Economic Plants and Biotechnology and the Yunnan Key Laboratory for Wild Plant Resources, Kunming Institute of Botany, Chinese Academy of Sciences, Kunming, China; ^2^ Southeast Asia Biodiversity Research Institute, Chinese Academy of Sciences, Nay Pyi Taw, Myanmar; ^3^ University of Chinese Academy of Sciences, Beijing, China; ^4^ Department of Botany, NSS College, Palakkad, India

**Keywords:** reactive oxygen species, oxidative stress, antioxidant, diabetes mellitus, mushroom polysaccharides

## Abstract

Diabetes mellitus is a prevalent metabolic and endocrine illness affecting people all over the world and is of serious health and financial concern. Antidiabetic medicine delivered through pharmacotherapy, including synthetic antidiabetic drugs, are known to have several negative effects. Fortunately, several natural polysaccharides have antidiabetic properties, and the use of these polysaccharides as adjuncts to conventional therapy is becoming more common, particularly in underdeveloped nations. Oxidative stress has a critical role in the development of diabetes mellitus (DM). The review of current literature presented here focusses, therefore, on the antioxidant properties of mushroom polysaccharides used in the management of diabetic complications, and discusses whether these antioxidant properties contribute to the deactivation of the oxidative stress-related signalling pathways, and to the amelioration of β-cell dysfunction and insulin resistance. In this study, we conducted a systematic review of the relevant information concerning the antioxidant and antidiabetic effects of mushrooms from electronic databases, such as PubMed, Scopus or Google Scholar, for the period 1994 to 2021. In total, 104 different polysaccharides from mushrooms have been found to have antidiabetic effects. Most of the literature on mushroom polysaccharides has demonstrated the beneficial effects of these polysaccharides on reactive oxygen and nitrogen species (RONS) levels. This review discuss the effects of these polysaccharides on hyperglycemia and other alternative antioxidant therapies for diabetic complications through their applications and limits, in order to gain a better understanding of how they can be used to treat DM. Preclinical and phytochemical investigations have found that most of the active polysaccharides extracted from mushrooms have antioxidant activity, reducing oxidative stress and preventing the development of DM. Further research is necessary to confirm whether mushroom polysaccharides can effectively alleviate hyperglycemia, and the mechanisms by which they do this, and to investigate whether these polysaccharides might be utilized as a complementary therapy for the prevention and management of DM in the future.

## Introduction

Diabetes is a non-communicable endocrine disorder that can lead to metabolic problems including diabetic retinopathy, renal failure, heart attack, stroke, and vascular damage of the lower limbs leading to amputation, all of which are secondary effects of hyperglycemia, hyperlipidemia, and oxidative stress (Jugran et al., 2021). Four types of diabetes are known: type 1 T1DM (insulin-dependent); type 2 (*non-insulin-dependent*); gestational diabetes; and a condition known as prediabetes (Saeedi et al., 2019). The most prevalent form is type 2 diabetes T2DM, while type 1 is the least common. Most of the pathogenic outcomes of both type 1 and type 2 diabetes are caused by immune-mediated destruction or malfunctioning of insulin-secreting β-cells from the endocrine pancreas and the islets of Langerhans (American Diabetes Association, 2018).

According to the World Health Organization (WHO), diabetes is now an epidemic illness, and has a high risk of morbidity and mortality. It affects over 422 million people globally, with the number anticipated to climb by 25% in 2030 and by a further 51% in 2045. The majority of people affected by diabetes are found in lower- and middle-income countries, and the disease kills about 1.5 million people each year (Saeedi et al., 2019). Cardiovascular disease is the primary cause of morbidity and death in people with T2DM (Zheng et al., 2018). About 9% of the global population has diabetes, of which 90% have type 2 diabetes mellitus, with China and India as the two most important epicenters. T2DM commonly occurs as a result of obesity, unhealthy lifestyle, and overconsumption of unhealthy foods containing excess red or processed meat, refined carbohydrates, and sugar-rich soft drinks. Maintaining a healthy body weight, eating a balanced diet, engaging in physical activity, giving up smoking, and reducing long-term alcohol consumption are measures to avoid T2DM (Zheng et al., 2018).

The glycation, oxidation and peroxidation of proteins, glucose and lipids, respectively, induces the formation of reactive oxygen species (ROS), reactive nitrogen species (RNS) and oxidative stress. Oxidative stress can lead to the destruction of cellular machinery and of various enzymes, as well as the augmentation of insulin resistance, which, in turn, is an important factor in the development of T2DM (Asmat et al., 2016).

Many drugs are available for the management of DM, including biguanides, α-glucosidase inhibitors (acarbose, miglitol, voglibose), sulfonylureas, thiazolidinediones, and management of DM can also be achieved through the maintenance of blood glucose level with insulin and hypoglycemic drugs. However, all the antidiabetic drugs listed above are limited by their side effects, which include hypoglycemic coma, kidney and liver problems (Marín-Peñalver et al., 2016). Hence, there is a great deal of interest in potential new and stronger phytotherapeutic compounds.

Polysaccharides derived from mushrooms have become a focus of research due to their various pharmacological properties, which include, antioxidant (Xiao et al., 2012), immunomodulatory (Meng et al., 2016), antihyperglycemic (Zhao et al., 2014), antidiabetic (Zhang et al., 2016), anti-tumor (Mao et al., 2015) and anti-inflammatory effects (Silveira et al., 2015).

Many species of mushrooms are used as foods, and have several nutritional components that have recently gained popularity, with some of them being indicated for the treatment of diabetes and its complications. Some mushroom species have been found to contain compounds that are able to manage blood glucose levels and affect the course of diabetes complications with no harmful side effects in clinical and/or experiments (Aramabašić Jovanović et al., 2021). Additionally, mechanistic investigations have led to the discovery of numerous polysaccharides in various mushroom species (Jovanović et al., 2017; Aramabašić Jovanović et al., 2021).

Anti-hyperglycemic activity has been demonstrated in polysaccharides and their associated proteins, phytonutrients, terpenoids, and some other bioactive components obtained from the fruiting bodies of certain medicinal mushrooms, the cultured mycelium, and cultivated broth. The antidiabetic actions of these compounds are mediated through a variety of mechanisms (Aramabašić Jovanović et al., 2021).

Several reviews on mushroom polysaccharides have been previously published (Lo and Wasser, 2011; De Silva et al., 2012; Ganesan and Xu, 2019; Jiang et al., 2020; Khursheed et al., 2020; Lindequist and Haertel, 2020; Al-Faqeeh et al., 2021). However, these studies do not sufficiently discuss oxidative stress, which results mainly through the action of RONS triggered by hyperglycemia, and which is known to contribute to the development and progression of diabetes and other complications.

There have been numerous reports of pre-clinical and clinical trials on the benefits of mushroom polysaccharides to human health, and polysaccharides isolated from several mushrooms have been investigated for antihyperglycemic activity, inhibition of glucose absorption, increasing β-cell mass and influence on the insulin signalling pathways (Aramabašić Jovanović et al., 2021).

In this study, we review the influence of mushroom polysaccharides on diabetes in numerous animal models, as well as highlighting the underlying molecular pathways linked to inflammatory variables, oxidative stress, and diabetes. The literature review was conducted using electronic databases. In many *in vivo* and *in vitro* investigations, mushroom polysaccharides have been demonstrated to have hypoglycemic, hypolipidemic, and antioxidant properties, improving pancreatic β-cell mass and ameliorating β-cell dysfunction. Furthermore, the polysaccharides have been demonstrated to enhances the activity of insulin signaling pathways *via* insulin receptors, activate the PI3K/Akt pathway and modifying the c-Jun N-terminal kinase (JNK) and mitogen-activated protein kinases (MAPKs) of the ERK pathway. Moreover, the administration of polysaccharides can effectively maintained the blood glucose levels, increasing insulin levels and regulated the expression of impaired carbohydrate metabolizing enzyme mRNA (Jayachandran et al., 2018).

We therefore, conducted a comprehensive and systematic review of mushroom polysaccharides with special emphasis on their antioxidant activity and the mode of action against the RONS which could be responsible for diabetic complications. The goal of the review is to examine the scientific data on 1) the medicinal and edible mushrooms used to prevent and treat diabetes mellitus and 2) the role mushroom polysaccharides play as antioxidants in reducing mitochondrial ROS.

This review provides a necessary overview of the current state of knowledge on the antioxidant properties of mushroom polysaccharides. It is clear from the papers studied that the amelioration of ROS by mushroom polysaccharides could be an effective strategy for the management of DM.

## The Theme and Methods Employed for the Literature Review

A bibliographic analysis of peer-reviewed material published between 1994 and 2021 was conducted using global scientific databases including Scopus, Pubmed, and Google Scholar*.* Search terms included *“mushrooms”, “diabetes”, “antioxidant”, “enzymatic and non-enzymatic antioxidant”, “antidiabetic”, “in vitro assays”, “free radicals”, “in vivo assays”, “insulin”, “insulin mimetics”, “beta cell”, “blood glucose”, “diabetes mellitus”, “polysaccharide”, “lentinan”,* and *“hypoglycaemic activity”*.

**FIGURE 1:**
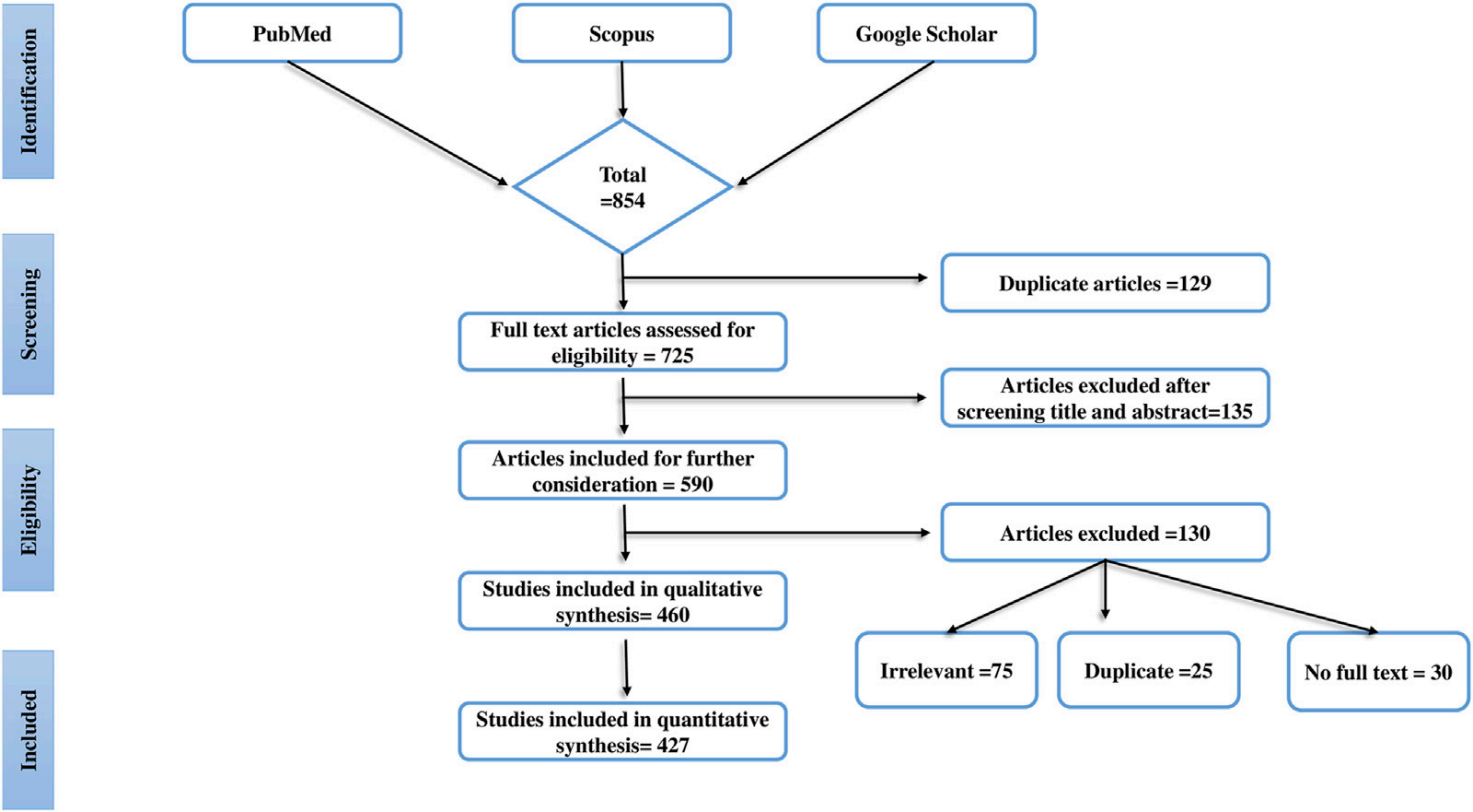
The progress of a systematic literature search is shown in this figure. The systematic literature search’s identification, screening, eligibility, and inclusion procedure is detailed and use the Preferred Reporting Items for Systematic Reviews (PRISMA) flow chart.

Overall, 854 articles were screened and initially selected as they established the antidiabetic activity of mushroom extracts or isolated components in experimental animals. Finally, a total of 427 articles were selected based on the following standard inclusion criteria: 1) the effects of edible and medicinal mushrooms on diabetic complications in animal models; 2) the effects of compounds isolated from mushrooms in the alleviation of diabetic complications; and 3) the effects of mushrooms/their fruiting bodies/mycelium/extracts in the alleviation of oxidative stress in diabetic animals, and there use as antihyperlipidemic and analgesics. To filter the published literature, we also applied the fourth criterion, 4) the exclusion of articles that did not follow standard scientific methods using positive and negative controls. This systematic review also excludes case report studies, cohort studies, congress papers, and letters to the editor. The database Mycobank (https://www.mycobank.org/) was used to corroborate the mushroom names. The research process, and also the factors that influenced our choice of papers, are shown in Figure 1. A preferred repeating item for systematic reviews and meta-analysis (PRISMA) flow diagram was used to represent the results of the article selection.

## What Is Oxidative Stress, and How can It Be Managed?

The generation of free radicals (RONS) is a normal process in the mammalian body. However, excessive free radical generation from endogenous or exogenous sources results in oxidative stress, which has major repercussions, including damage to proteins, lipids, and DNA, as evidenced by phagocytosis, neutrophil function, and, particularly, shear stress-induced vasorelaxation. Oxidative stress causes a rise in mitochondrial superoxide production in the endothelial cells of both large and small arteries and in the heart, resulting in a range of micro- and macrovascular complications (Chandra et al., 2019).

Oxidative stress is defined as the increased accumulation and/or inadequate clearance of highly reactive molecules, particularly the free radical RONS such as superoxide (^•^O_2_-), hydroxyl (OH^•^), peroxyl (^•^RO_2_), hydroperoxyl (^•^HRO_2_-), nitric oxide (^•^NO) and nitrogen dioxide (^•^NO_2_) and the non-radical H_2_O_2,_ hypobromous acid (HOBr), hypochlorous acid (HOCl), ONOO^−^, nitrous oxide (NO_2_), and alkyl peroxy nitrates (RONOO) (Evans et al., 2002). The most studied of these reactive molecules are O_2_-, NO, and ONOO^−^, which play critical roles in diabetic cardiovascular issues. The creation of one ROS or RNS may result in the creation of others *via* radical chain reactions. ^•^O_2_- is created by oxidases such as NAD(P)H oxidase, xanthine oxidase, cyclooxygenase, and, in certain cases by endothelial nitric oxide synthase (eNOS). The mitochondrial electron transport chain also generates ^•^O_2_- during normal oxidative phosphorylation, which is essential for ATP production (Brownlee and Cerami, 1981). Another highly reactive radical, ^•^OH can be formed from H_2_O_2_ in the presence of catalyst elements such as iron or copper (Evans et al., 2002). Trace metals like Fe, Cu, and Mg promote the formation of ROS such as HO^•^ through the Fenton reaction {(Fe^2+^ + H_2_O_2_ → HO^•^ + HO^−^ + Fe^3+^)} catalyzed by Fe^2+^ (Jiang, 2014).

Under normal conditions, antioxidant defense systems remove O_2_-^•^ quickly. It is transformed to H_2_O_2_ by manganese superoxide dismutase (Mn-SOD) in the mitochondria and by copper (Cu)-SOD in the cytoplasm. Glutathione peroxidase (GPx), SOD and CAT (catalase) transform H_2_O_2_ to H_2_O and O_2_ in the mitochondria and lysosomes (Evans et al., 2002).

Generally, antioxidants protect against adverse effects of ROS the oxidation substrates including DNA, proteins, fats, oils, and food (Rozoy et al., 2012). They are very effective in protecting against oxidative stress generated by free radicals in three ways: 1) acting as chemical free radical scavenging agents, and therefore, helping to reduce ROS levels; 2) acting as chelating agents, and preventing free radical formation by substituting electrons with free radicals; or 3) acting as phenolic agents, preventing free radical formation by complexing with metals (Zhang et al., 2020).

GPx, SOD and CAT catalyze the oxidation of O_2_^•−^ and, similarly, vitamin C also acts as a ROS scavenger by transferring an electron to a substrate such as O_2_^•−^ (Banerjee and Vats, 2014; Lykkesfeldt. et al., 2014). Vitamin E is a phenolic molecule thought to function by transferring a hydrogen atom from its phenolic group also on chromanol to lipid peroxide radicals in membranes and/or LDL particles, in addition to being an ROS scavenger and an active iron chelator (Jiang, 2014). The oxidized form of vitamin E is normally persistent but does not engage in oxidative chain reactions. Chelating substances (including proteolysis-induced peptides, carnosine, and anserine) also help lower ROS production by complexing specific metals (Fe, Cu, and Mg) (Zhang et al., 2020). Additional antioxidants which indirectly regulate ROS levels include vitamin D, vitamin B9, coenzyme Q10 (CoQ10), N-acetylcysteine (NAC), and lipoic acid (LA) (Farhangi et al., 2017).

## Pathophysiology and Complications of Diabetes Mellitus

Several diseases are known to be caused by oxidative stress, including rheumatoid arthritis, diabetes, and some cancers. Recent studies have demonstrated the role of oxidative stress caused by free radicals in the pathogenesis of both T1DM and T2DM, and the maintenance of severe diabetic complications because of the capacity of free radicals to damage lipids, proteins, and DNA (Ayepola et al., 2014). The oxidation of glucose, non-enzymatic protein glycation, and higher lipid peroxidation results in free radical-induced oxidative stress, leading to damage to the cellular machinery and enzymes as well as to the rise in insulin resistance which ultimately results in DM (Maritim et al., 2003; Pham-Huy et al., 2008).

**FIGURE 2:**
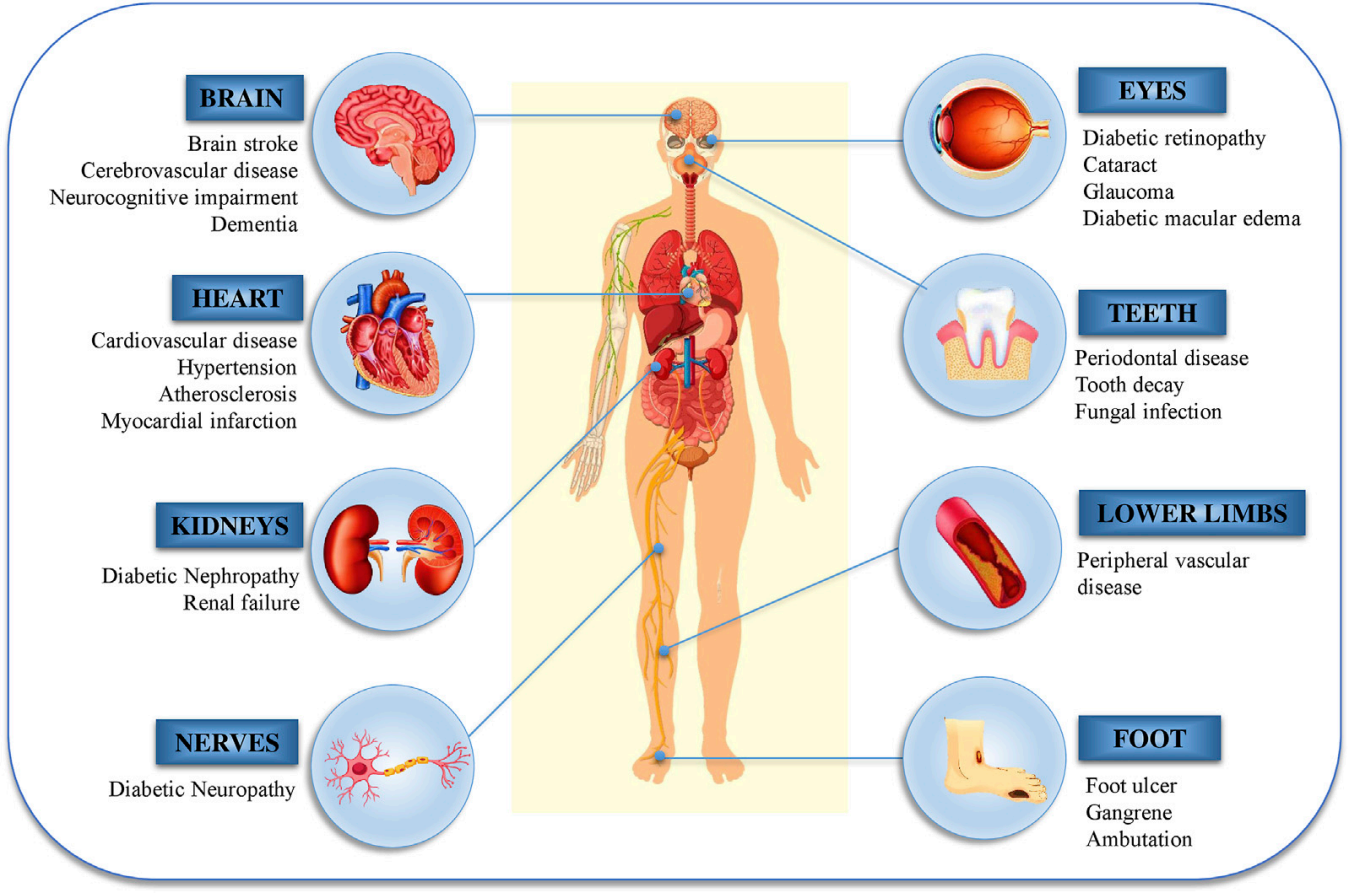
Diabetic nephropathy, retinopathy, and neuropathy are the most common micro- and macro-vascular consequences of diabetes.

Another major source of oxidative stress in diabetes mellitus is mitochondrial oxidative metabolism. A part of the consumable oxygen is converted to water through phosphorylation in the mitochondria, while the rest forms oxygen free radicals (˙O_2_), which are primary ROS that are converted to additional reactive species such as peroxynitrite (ONOO^−^), hydroxyl radicals (˙OH), and hydrogen peroxide (H_2_O_2_) (Moussa, 2008; Erejuwa, 2012). The cellular pathophysiology of DM and its effects are shown in Figure 2.

Free radicals and oxidative stress increase the risk of coronary artery disease, neuropathy, nephropathy, retinopathy, and stroke, all of which are complications of diabetes. Several studies have examined the relationship between diabetes and oxidative stress through the investigation of biomarkers of DNA damage and byproducts of lipid peroxidation (Ayepola et al., 2014).

Damages to Lipids, proteins, and DNA are among the indicators of oxidative stress in DM, as alterations in enzymatic systems, lipid peroxidation, poor glutathione metabolism, and low vitamin C levels (Asmat et al., 2016). Hyperglycemia promoted oxidative stress in the blood vessels of during diabetics (Ceriello, 2006). Moreover, hyperglycemia has been linked to the stimulation of many metabolic pathways, including the NF-κB, c-Jun N-terminal kinases/stress-induced activated protein kinases (JNK/SAPK), and p38-MAPK (p38 mitogen-activated protein kinases) pathways, along with insulin resistance and β-cell dysfunction (Evans et al., 2003). Diabetes-related oxidative stress induced by excessive glucose can result in insulin resistance, microangiopathy, dyslipidemia and atherosclerosis (Giugliano et al., 1995).

## Sources of Oxidative Stress Linked to Diabetes

**FIGURE 3:**
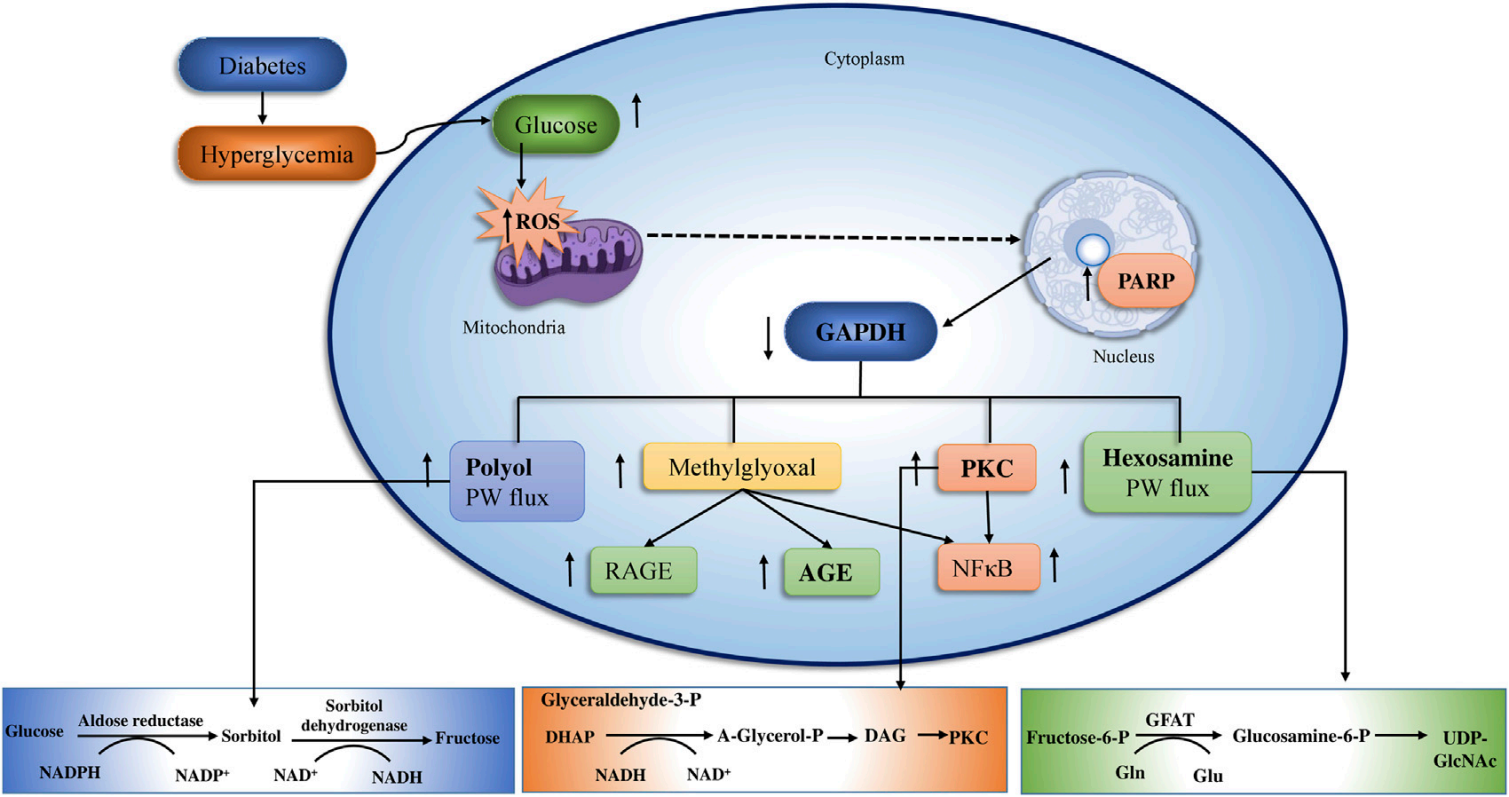
The above figure depict the underlying pathway of hyperglycemia-induced mitochondrial dysfunction. Oxidative stress and vascular endothelial dysfunction Four major mechanisms connected to vascular endothelial cell failure include PKC, AGEs/RAGE, polyol, and hexosamine. The polyol and AGEs/RAGE pathways increase ROS levels in endothelia cells, that activates NF-κB, ultimately induces inflammation and thrombosis in vascular endothelia by raising the transcription of VEGF, VCAM-1, and ET-1. (adopted from Brownlee, 2001).

Long-term glucose augmentations cause diabetic complications in target organs in patients with T2DM. Hyperglycemia produces glucose toxicity in tissues with an insulin-independent glucose absorption route. The pathogenic consequence of excessive glucose, perhaps in conjunction with fatty acids is an increase in cellular peroxynitrite (Rolo and Palmeira, 2006). The peroxynitrite damages DNA by producing poly ADP ribose polymerase (PARP), which reduces ATP production and promoted ADP-ribosylation of glyceraldehyde-3-phosphate dehydrogenase (GADPH), leading to diabetic complications (Nishizuka, 1995; Chung et al., 2003; Robertson, 2004; Nagai et al., 2014; Ighodaro, 2018) (Figure 3).

Elevated intracellular ROS increase defective angiogenesis in response to ischemia, activate a variety of pro-inflammatory pathways and also produce long-term epigenetic changes that drive chronic pro-inflammatory gene expression after glycemia is reversed (“hyperglycemic memory”). The pathway-selective insulin resistance enhances mitochondrial ROS formation *via* free fatty acids that deactivate anti-atherosclerosis enzymes, and thus ROS contribute to atherosclerosis with cardiomyopathy in patients with T2DM. Moreover, overexpression of SOD has been shown to prevent diabetic retinopathy, nephropathy, and cardiomyopathy (Giacco and Brownlee, 2010).

## Historical Perspective of Mushrooms

The consumption of mushrooms is believed to be as ancient as the history of food gathering (Boyden, 1973), and many human cultures still have a culture of harvesting and consuming wild-grown mushrooms as part of their diet. For millennia, many societies have utilized mushrooms as either a crucial resource for sustenance or healing (Power et al., 2015; Kotowski, 2019). Cultivated mushrooms have now also appeared on the market, and mushroom farming is becoming more popular across the world (Srikram and Supapvanich, 2016; Sevindik et al., 2020). A total of 1069 mushroom species have been documented as being edible out of 14,000 recorded mushroom species, including 270 with potential benefit as medicines to improves human health (El Sheikha and Hu, 2018).

The output and commercial value of macrofungi have increased globally. As a consequence of increased mushroom consumption, the global mushroom industry is expected to approach US$ 62.19 billion by 2023 (Niego et al., 2021).

Mushroom-derived medicines are employed in contemporary clinical therapies in Taiwan, Japan, China, South Korea, and other Asian nations. Several biologically active compounds have been isolated from these formulations, including the high-molecular-weight polysaccharides (such as β-D-glucans and glucuronoxylomannans), proteins, polysaccharide-protein complexes, lipopolysaccharides, glycoproteins, and lectins, as well as low molecular weight metabolites such as lactones, terpenoids, alkaloids, sterols, and other phenolic chemicals (Vitak et al., 2017).

Carbohydrates make up the majority of the dry matter in mushrooms, and comprise both soluble (rhamnose, glycogen, mannitol, and glucose) and non-soluble (chitin, mannans, and β-glucan) carbohydrates (Wasser and Weis, 1999). In addition to the presence of Vitamins B and D, minerals and amino acids, mushrooms have been found to contain bioactive components including polysaccharides (37–48%), fibers (13–24%), peptides, proteins (20–25%), proteoglycans, phenolic compounds, terpenes, and lectins (Samsudin and Abdullah, 2019; Yadav and Negi, 2021). Mushrooms also contain ergosterol and lanosterol, which have antihypertensive, immune-modulating, anticancer, antibacterial, antioxidant, anti-hypocholesterolemic, anti-hyperglycemic, antiviral, antifungal, anti-inflammatory, and anti-osteoporotic properties (Wasser and Weis, 1999).

For centuries, diverse mushroom species have been used to treat DM symptoms (e.g. honey urine or overproduction of urine) (Zong et al., 2012). Research into antidiabetic polysaccharides has become more important and therefore, mushroom polysaccharides should be tested for their efficacy in DM control (Ganesan and Xu, 2019).

## Bioactive Polysaccharides From Mushrooms

Wild mushrooms have been shown to contain more fibre and bioactive compounds than do on cultured mushrooms (Srikram and Supapvanich, 2016; Sevindik et al., 2020).

Mushrooms polysaccharides are physically different from other types of polysaccharides in their molecular weights, chemical properties, degree of branching, skeleton lengths, three-dimensional orientation, and other characteristics (Fan et al., 2012). Unlike most plant polysaccharides, which have a complex structure made up of pectic polysaccharides (Ndeh et al., 2017), the majority of mushroom polysaccharides are either β-or D-glucan, or a mixed glucan. The observed glucans are present a diversity of linkages, and are usually linked as (1 → 3), (1 → 6)-D-glucans, (1 → 3)-D-glucans, and (1 → 6)-D-glucans (Maity et al., 2015). β-D-glucans function as biological response modifiers, stimulating the production of macrophages, NK cells, T cells, and cytokines (Bhanja et al., 2012; Zhang et al., 2015a). Several β-D-glucans also have the ability to stimulate splenocytes and thymocytes, as well as having significant antioxidant properties (Nandi et al., 2014; Maity et al., 2015).

Edible mushrooms have also been found to contain a variety of immunomodulating heteroglycans, which are made up of sugars including mannose, galactose, as well as fucose and glucose in varying molar ratios (Bhunia et al., 2012).

Recent studies have suggested that polysaccharides have a variety of antidiabetic functions, including antioxidant, hypoglycemic, hypolipidemic, and *via* anti-inflammatory activities that help to increase pancreatic β-cell mass as well as alleviating β-cell disorders. It stimulates the PI3K/Akt pathway and modifies the ERK/JNK/MAPK mechanisms after enhancing insulin signal transduction pathways through interaction with the insulin receptors (Ganesan and Xu, 2019).

**FIGURE 4:**
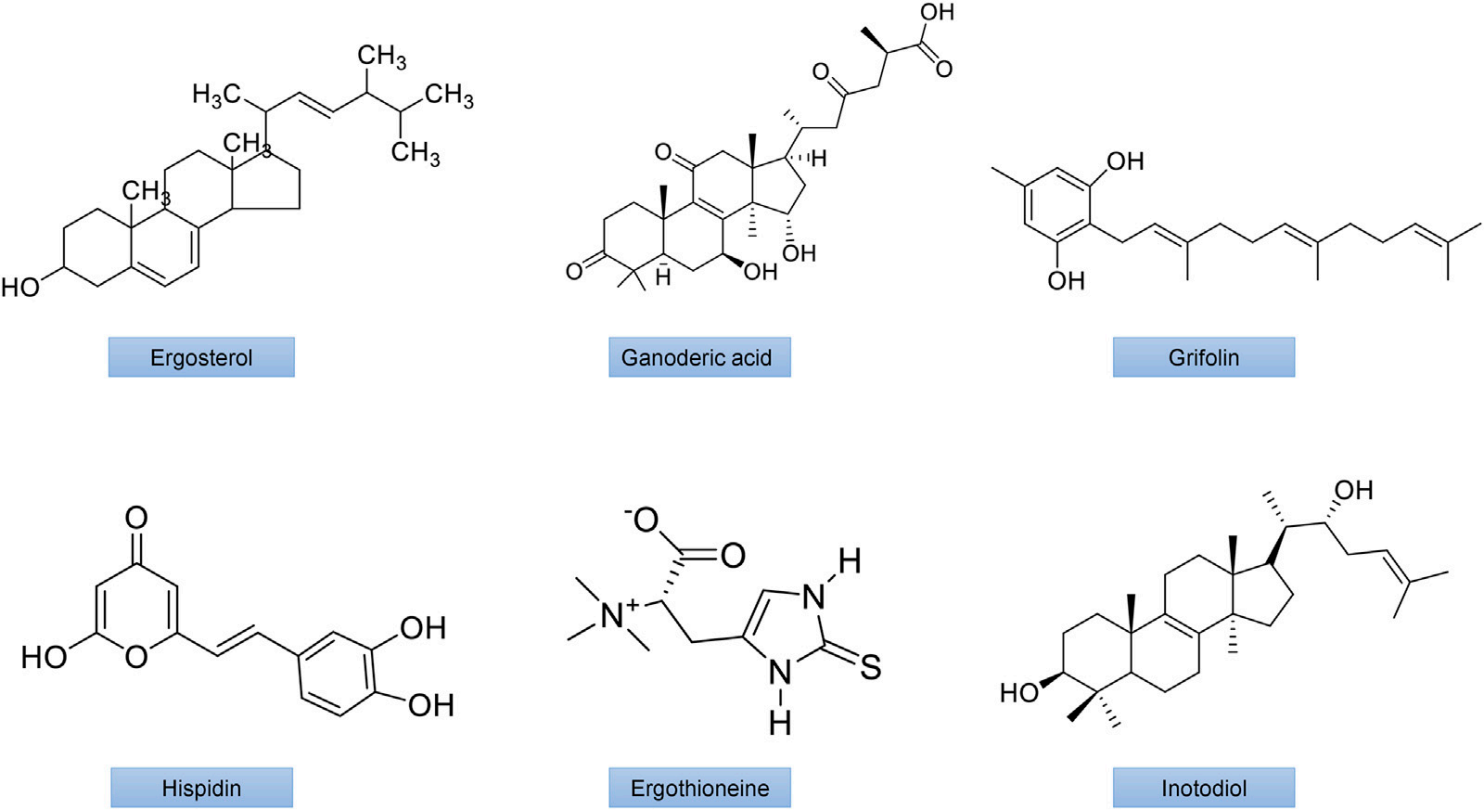
The chemical structures of important mushroom antidiabetic activity compounds and polysaccharides. Ergosterol from *Inonotus obliquus*; Ganodermic acid from *Ganoderma lucidum;* Grifolin from *Grifola frondosa;*Hispidin from *Phellinus linteus*; Ergothioneine from *Agaricus bisporus*; Inotodiol from *Inonotus obliquus*.

**FIGURE 5:**
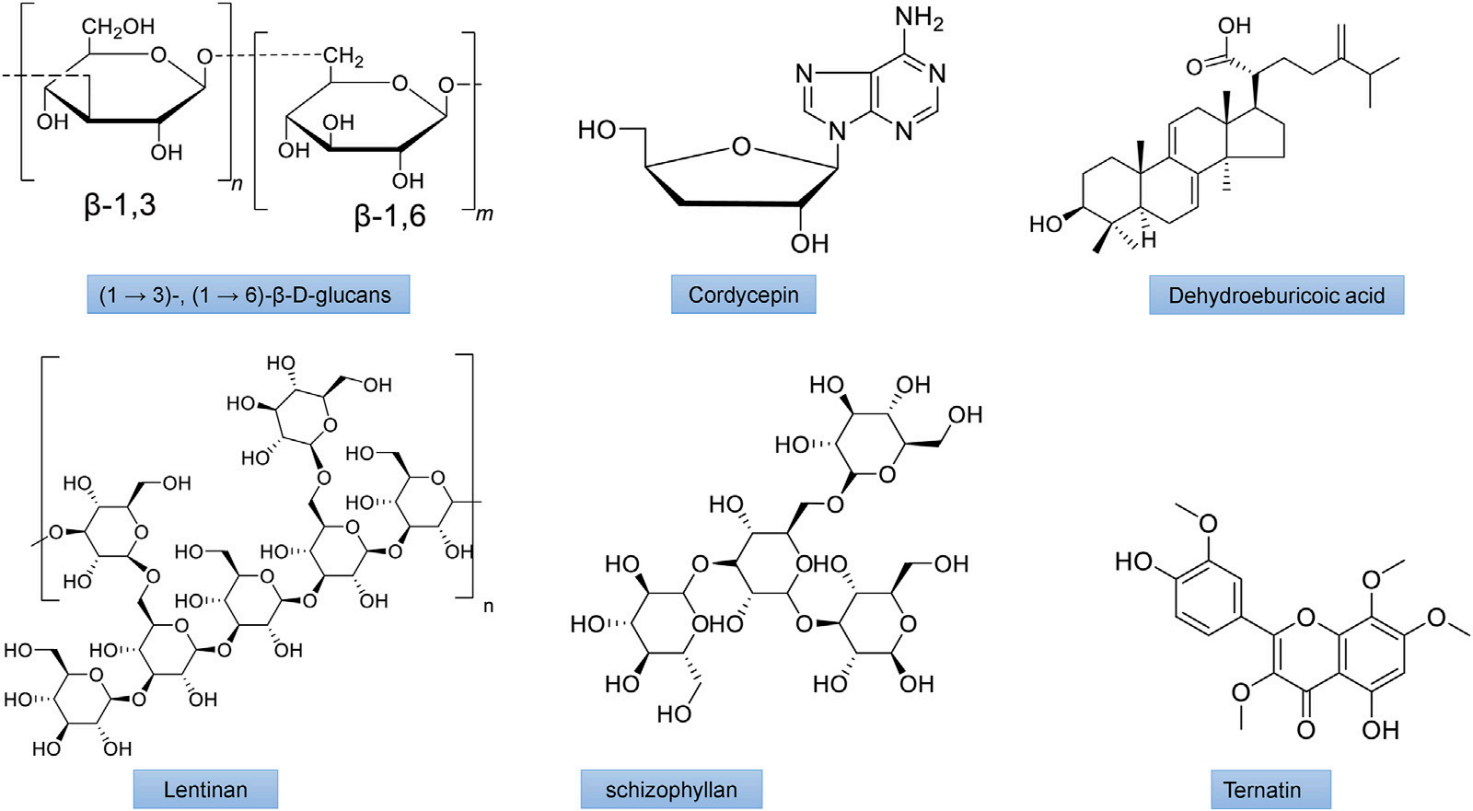
The chemical structures of important mushroom antidiabetic activity compounds and polysaccharides. β-1,3/1,6 glucan from *Agaricus blazei*; Cordycepin from *Cordyceps militaris*; Dehydroeburicoic acid from *Antrodia camphorate*; Lentinan from *Lentinus edodes*; Schizophyllan from *Schizophyllum commune*; Ternatin from *Coriolus versicolor*.

Numerous bioactive polysaccharides isolated from mushrooms (Figures 4, 5), such as lentinan from *Lentinus edodes* (Berk.) Singer, Grifron-D from *Grifola frondosa* (Dicks.) Gray, schizophyllan from *Schizophyllum commune* Fr., polysaccharide K (PSK or krestin) from *Coriolus versicolor* (L.) Quel., glucans and heteroglycans from *Lentinus squarrosulus* (Mont.), and similar polysaccharides from many other mushrooms including *Pleurotus florida* Singer, *P. ostreatus* (Jacq.ex,fr), *Termitomyces robustus* (Beeli) R. Heim and *Calocybe indica* Purkayastha and A. Chandra have also been reported to have potentially immunomodulating, antioxidant, antiviral, anti-inflammatory, anticarcinogenic, and neuroprotective and anticancer activities. The polysaccharide lentinan inhibits activation of NF-κB, JNK and p38 MAPK signaling pathways as well as ROS formation. It also has anti-apoptotic and anti-dysfunctional activity in STZ-induced diabetic rats (Zhang X. et al., 2016). PSK has been demonstrated to improves insulin resistance and hyperlipidemia through controlling the expression of inflammatory cytokines (Xu et al., 2016).

The polysaccharide hispidin, isolated from *Phellinus linteus* (Berk. and M.A. Curtis) Teng. displays antioxidant activity and can improve β-cell condition by inhibiting H_2_O_2_ induced apoptosis and is also able to upregulate insulin secretion (Pei et al., 2015; Maity et al., 2021). Similarly, biologically active polysaccharides obtained from commercially significant macrofungi, including *Ganoderma lingzhi* S.H.Wu, Y. Cao and Y.C. Dai and *G. sichuanense* J.D. Zhao and X.Q. Zhang., have shown hypoglycemic, hypolipidemic, antidiabetic, and antioxidant activities in the blood, liver, and skeletal muscles of STZ-induced T2DM rats blood, as well as in the blood, heart, spleen, liver, and kidney of male Kunming mice (Liu et al., 2019; Xu et al., 2019; Chen et al., 2020). In mice with diabetes induced by high fat diet (HFD) and streptozotocin, polysaccharides isolated from *G. frondosa* were shown to have potential hypoglycemic and prebiotic properties (Guo et al., 2020).

Polysaccharides from *Pleurotus eryngii* (DC.) Quel., demonstrated hypoglycemic activity (Chen L. et al., 2016) in KK-Ay mice with hyperlipidemia, while those from *P. ostreatus* (Jacq.) P. Kumm., were able to control dyslipidemia in STZ-induced diabetic rats and fat-emulsion-induced hyperlipidemia rats (Zhang Y. et al., 2017; Wisbeck et al., 2017). In HFD-induced hyperlipidemic mice, intracellular as well as extracellular polysaccharide preparations of *Trametes versicolor* (L.) Lloyd. demonstrated antihyperlipidemic properties are shown in Figures 4, 5 (Huang et al., 2020).

Polysaccharides and certain other important compounds isolated from mushrooms. The sections that follow focus on mushroom polysaccharides that have been discovered in recent years, as well as the mechanisms underpinning their various antidiabetic activities.

## Mechanisms Underlying the Anti-oxidative Stress Activity of Mushroom Polysaccharides

Antioxidants are beneficial because they eliminate ROS, which can damage DNA and essential proteins. Enhanced GSH and the increased activities of the liver oxidative enzymes CAT and SOD are typically linked to the antioxidative effects of mushroom polysaccharides *in vivo* (Xiao et al., 2012).

**FIGURE 6:**
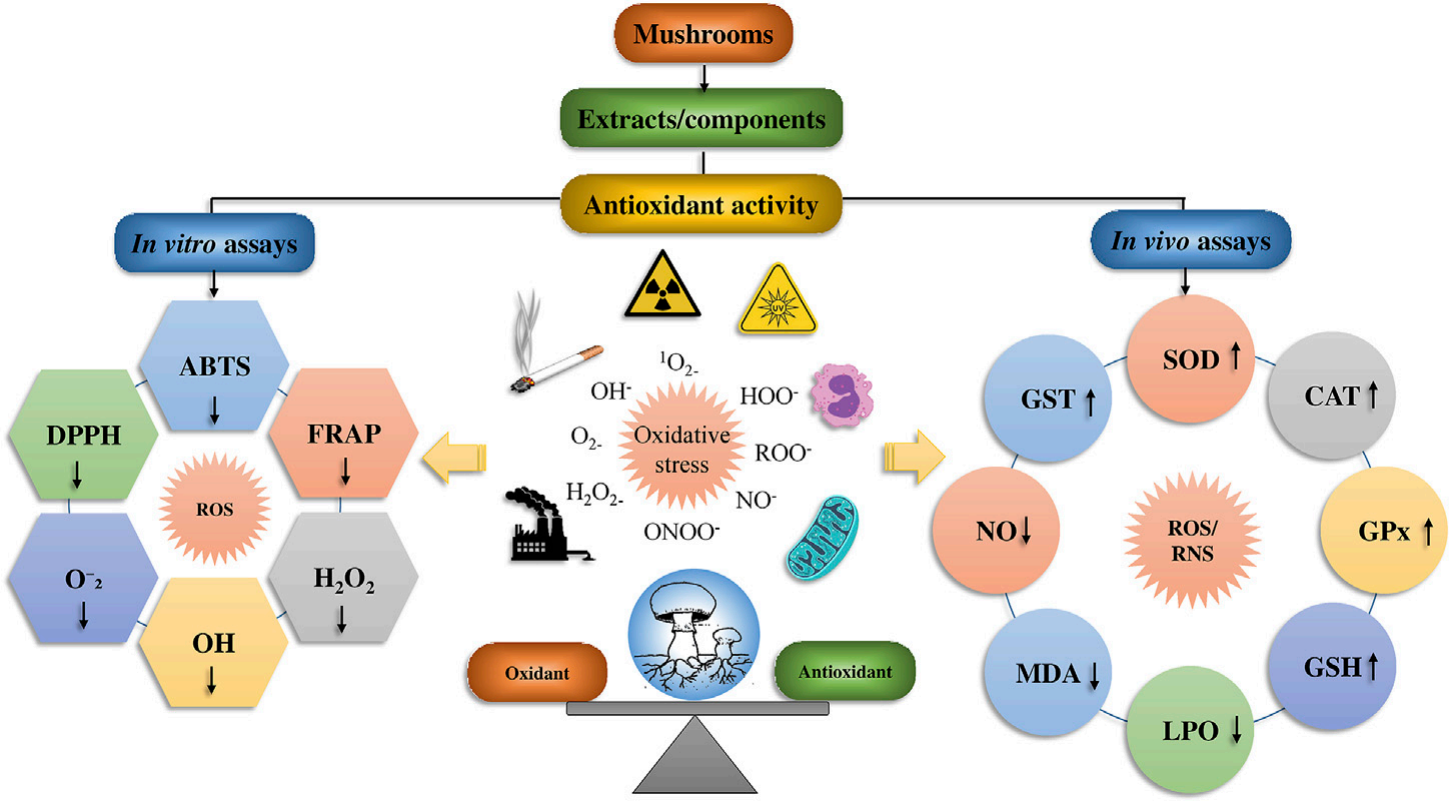
The biomechanics of the mushroom’s antioxidant effect and its bioactive mushrooms compounds.

The antioxidant activity of mushroom compounds has already been extensively studied, and effects including lipid peroxidation and MDA (malondialdehyde) suppression, decrease in low-density lipoprotein (LDL) in humans, chemical free radical scavenging activity, and other factors were well known. Mushrooms contain many different compounds, with some being unique to particular mushroom species. The fruiting bodies and mycelium in wild or cultured mushrooms may contain phenolics, polysaccharides, tocopherols, flavonoids, carotenoids, glycosides, ergothioneine, and ascorbic acid. Of these compounds, the polysaccharides are regarded as the main contributors to the observed antioxidant activity (Sánchez, 2017). The antioxidant potency of mushroom polysaccharides can be ascertained using a variety of chemical assays, such as azinobis-3-ethylbenzthiazoline-6-sulphonate (ABTS) and 1,1-diphenyl-2-picrylhydrazyl (DPPH) assays, reducing power, hydroxyl radicals, nitric oxide, O_2_^•−^, lipid peroxide, as well as ferric-reducing antioxidant power (FRAP) and other techniques (Figure 6).

### Chemical Free Radical Scavenging Activity Assays

Numerous chemical assay studies have demonstrated that certain extracts and constituents of mushrooms, including phenolics, triterpenoids, and polysaccharides, have anti-radical activities (Barros et al., 2008; Kozarski et al., 2011; Mahendran et al., 2012; Reis et al., 2012; Liu J. et al., 2013; Jaszek et al., 2013; Rajoriya et al., 2015b; Jaworska et al., 2015; Sevindik, 2019) (Supplementary Table S1). Kim et al. (2008) reported that the phenolic compounds (caffeic acid, catechin, gallic acid, naringin, ferulic acid, myricetin, pyrogallol, protocatechuic acid, homogentisic acid, and quercetin) present in fresh mushroom fruiting bodies showed high DPPH radical and O_2_^•–^ scavenging activity. The free radical scavenging activities of four mushroom species were assessed by Kozarski et al. (2011), who found that polysaccharide extracts derived from *G. lucidum* correlated substantially with both the reducing power and the total quantity of phenols and β-glucans.

Maity et al. (2014) investigated the chemical free radical scavenging activity of a branched β-D-glucan (PS-I) extracted from the edible mushroom *Entoloma lividoalbum* (Kühner and Romagn.) Kubicka. The authors found PS-I scavenged ^•^OH and O_2_^•–^ exhibiting EC_50_ values of 480 μg/ml and 150 μg/ml, respectively, and the fungus also had reducing power and total antioxidant capacity. In the same study, MGPS polysaccharide was isolated from an aquous exctract of *Meripilus giganteus* (Pers.) P. Karst., and was found to have substantial free radical scavenging activities in terms of ferrous ion chelating capacity and ^•^OH and O_2_^•–^ radical scavenging properties (Maity et al., 2014). A decoction of the edible fungus *Lentinus sajor-caju* (Fr.) Fr., was found to contain a novel anti-radical agent heteroglycan that showed DPPH, OH and ABTS radical scavenging abilities as well as reducing power and the capacity to chelate ferrous ions (Maity et al., 2014). Furthermore, in another study β-D-glucan extracted from *E. lividoalbum* showed ^•^OH and O_2_^•–^ radical scavenging activities with IC_50_ values of 400 μg/ml and 75 μg/ml and optimal reducing power at 470 μg/ml (Maity et al., 2015).

Pattanayak et al. (2015) demonstrate that the antioxidant heteroglycan from the aqueous extract of the edible fungus *Termitomyces clypeatus* R. Heim (comprising (1 → 3)-α-d-galactopyranosyl, (1 → 3)-α-d-mannopyranosyl, (1 → 3)-α-d-glucopyranosyl, (1 → 3)-β-d-glucopyranosyl, (1 → 6)-β-Dglucopyranosyl, and (1 → 6)-α-d-galactopyranosyl), showed ferrous ion chelating and O_2_^•–^ scavenging capabilities, as well as strong reducing power with an EC_50_ ranging from 180–475 μg/ml (Pattanayak et al., 2015).

Wang et al. (2015) and Li et al. (2016) reported the Fe^2+^ chelating and ABTS, DPPH, O_2_^−^, and OH radical scavenging activities of polysaccharides extracted from *Calocybe gambosa*, (Fr.) Don., as well the extract and polysaccharides from *Pleurotus eryngii* (DC.) Quel. Similarly, Khan et al. (2017) also described chemical free radical scavenging activity through DPPH and ABTS radical scavenging and metal ion chelation by the β-glucans extracted from three edible mushroom species, *Agaricus bisporus* (J.E. Lange) Imbach, *P. ostreatus* (Jacq.) P. Kumm., and *Coprinus comatus* (O.F. Müll.) Pers.

The β-glucan extracted from *Coprinus atramentarius* (Bull.) Fr. also showed strong free radical scavenging activity (Wu et al., 2014). Similarly, the polysaccharides obtained through homogeneous solvent extraction or alkali extraction of *Pleurotus tuber-regium* (Fr.) Singer. also showed substantial chemical free radical scavenging activity that was positively linked with the total phenolic content, showing that edible mushrooms have had the potential to assist as natural free radical scavengers through the inherent ability of phenolic compounds to inhibit lipid peroxidation (LPO) (Wu et al., 2014).

Therefore, many studies have demonstrated the chemical free radical scavenging ability of mushrooms in diverse chemical assays. Although, the chemical free radical scavenging tests such as DPPH or FRAP using chemicals are useful as a starting point in the evaluation of chemical free radical scavenging ability, the test by itself alone is not a valid proof to claim the test substance antioxidant effect (Heinrich et al., 2020). Chemical free radical scavenging ability investigations are restricted because they cannot simulate the interaction of a phytocompound with all of these chemicals and cell types found within a complex organ. In addition, they can only be examined in isolation, while the human body is a dynamic environment with various pathways and cells that are constantly communicating with one another. This makes the these chemical free radical scavenging investigations challenging to predict the intricacies of possible interactions (Gargiulo, 2018). However, further *in vivo* experiments/pharmacological assays can confirm their antioxidant activity by testing in rodents models (Heinrich et al., 2020).

### *In vivo* Assays

Many *in vivo* studies have confirmed that mushroom extracts and compounds isolated from them are able to enhances the activities of enzymatic antioxidants such as SOD, CAT, GSH and GPx that are involved in the removal of damaging ROS (Ribeiro et al., 2015). Shi et al. (2013) *confirm the antioxidant activity of G. lucidum* *polysaccharides (GLP), which is as a result of the provided hydrogen atom interact with radicals to terminate the radical chain reactions* and therefore lower the levels of LPO and MDA (Jia et al., 2009). GLPs also protect cells against oxidative damage by enhancing activities of CAT and SOD, and by preventing the depletion of GSH-Px (Zhang et al., 2016; Lu et al., 2020a). The various studies (summarized in Supplementary Table S1) into the antioxidant properties of mushroom polysaccharides point to their functioning as potent antioxidants, preventing radical-mediated toxicity and ultimately protecting tissue damage from ROS and other free radicals.

The most important class of mushroom polysaccharides with antioxidant activity are the β-D-glucans. The β-D-glucans may be effective in humans in the restoration of the immune system, as well as in the fight against DM, cancer and infectious disorders (Khan et al., 2017). Various other pharmacological compounds generated from mushroom polysaccharides including schizophyllan, lentinan, grifolan, polysaccharide peptide complex (PSP) and polysaccharide-protein complex (PSK), have demonstrated promising therapeutic benefits in the management of DM, as well as exhibited their antioxidant capacity (Khan et al., 2017). Despite their obvious advantages as antioxidant agents (mushroom polysaccharides), a more complete examination and evaluation of the effectiveness and safety of the present treatment approach is necessary. In the future, further research into the pathophysiology of oxidative stress and well-planned antioxidant treatment, especially with a mechanistic approach in diabetes patients, should be a major priority.

## Antioxidant Potential of Mushrooms and the Mechanisms Underlying Their Efficacy in the Management of DM

Oxidative stress has long been associated with and implicated in the aetiology of DM (Chung et al., 2003), and insulin resistance has been linked to the generation of ROS and oxidative stress in recent studies (Nagai et al., 2014; Ighodaro, 2018). *In vitro* tests and animal models of diabetes suggests that antioxidants increase insulin sensitivity and can alleviate complications brought on by insulin resistance (IR) in DM (Folli et al., 2011).

Zahid et al. (2020) showed that an ethanol extract of *Fomitopsis pinicola* (Sw.) P. Karst. was able to reduce STZ-induced hyperglycemia at a high dosage (300 mg/kg). In biochemical tests, treatment with high doses of *F. pinicola* extracts increased high-density level lipoprotein- C (HDL-C) and reduced total cholesterol (TC), triglyceride (TG), and LDL-C. Likewise, treatment with *F. pinicola* extract was able to protect organ tissues against oxidative stress through restoring the levels of CAT, SOD and GSH-Px enzymes. Meanwhile, levels of MDA were reduced following *F. pinicola* extract treatment (Zahid et al. (2020).

**FIGURE 7:**
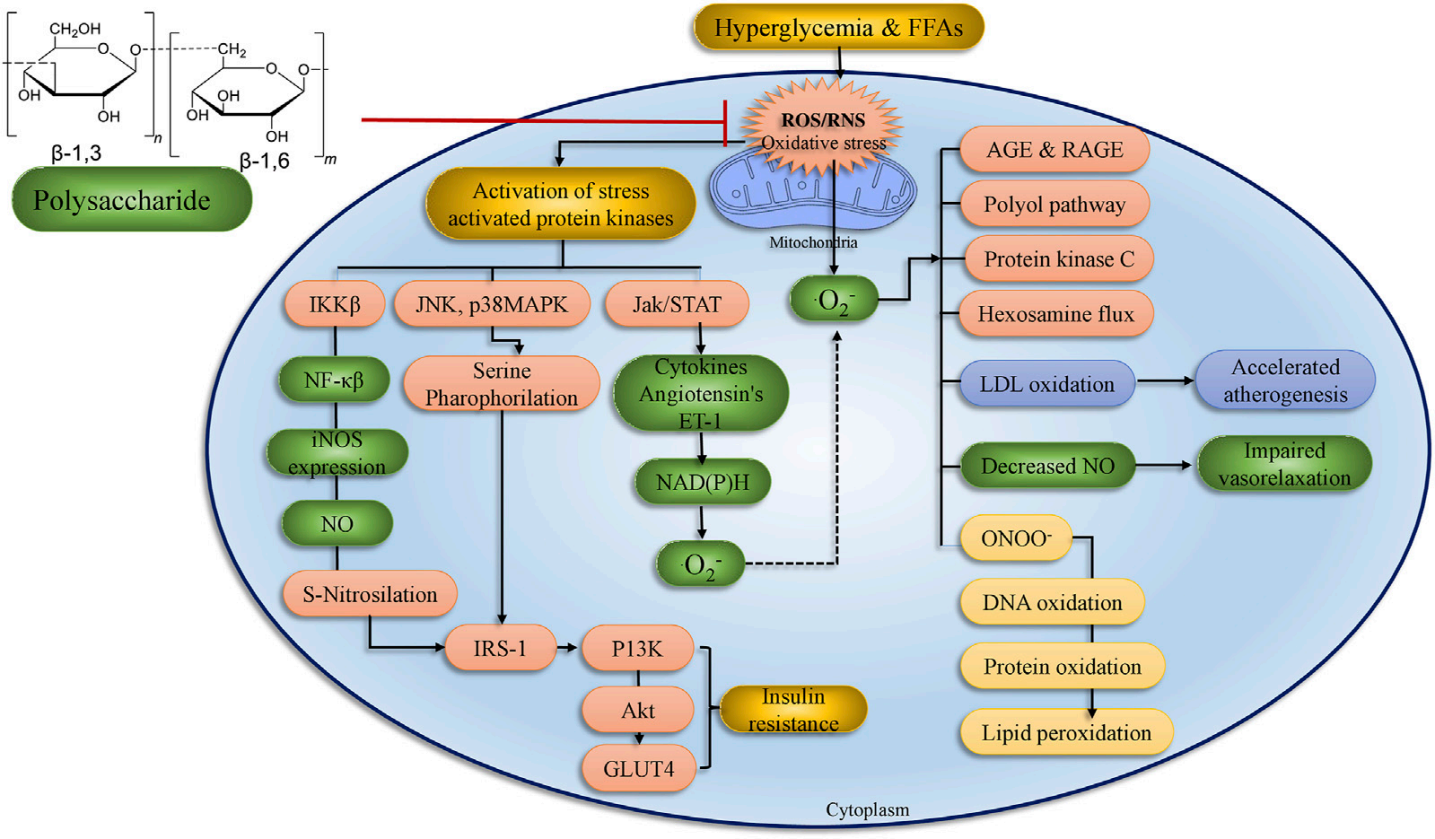
Treatment with polysaccharides has a positive impact on diabetes problems in organs and tissues. ROS formation by hyperglycemia, free fatty acids, and cytokines has a cumulative impact and process. Stress-sensitive pathways such as polyol, advanced glycation end products, PKC, and hexosamine flux are activated when mitochondrial ROS levels are too high. The current original study text goes into great detail on the processes. IRS, insulin receptor substrate; ROS, reactive oxygen species; IR: insulin receptor; NF-κB, Nuclear factor-kappa B; JNK, c-Jun N-terminal kinases; PI3K, phosphoinositide 3-kinase; LPO, lipid peroxidation; AKT, serine/threonine-specific protein kinase; RNS, reactive nitrogen species (adopted from Banerjee and Vats, 2014).

Treatment with bioactive mushroom compounds has been shown in numerous studies to have a therapeutic effect in the management of DM and its complications. The antidiabetic actions of different mushroom polysaccharides are given in Supplementary Tables S1, S2 and Figure 7. Supplementary Table S2 summarizes anti-hyperglycemic activity and anti-oxidant function of various polysaccharides, phenolics, terpenoids, and other chemicals isolated from mushrooms. Figures 4, 5 detail the structures of these components.

Oral treatment with a purified polysaccharide extracted from *P. eryngii* using hot water demonstrated hypolipidemic and hypoglycemic properties in high fat-fed KKAy mice. Body weight gain was slowed, and reduced the level of plasma insulin, serum triglyceride, low-density lipoprotein cholesterol, and blood glucose (Chen L. et al., 2016).

Polysaccharides derived from *Pleurotus sajor-caju* (Fr.) Singer were also found to reduce body weight gain and fasting blood glucose levels and serum insulin levels in response to polysaccharide treatment, similar to the positive control treated with metformin (Kanagasabapathy et al., 2012). These data suggested that certain mushroom polysaccharides may be able to, at least partially, replace western medical treatments, and lay the groundwork for the creation of novel medications. Furthermore, in mice fed on a high-fat diet, the polysaccharide-rich β-glucans from *Pleurotus sajor-caju* mushrooms had a hypoglycemic effect, mediated by the upregulation of insulin-response glucose-transporter (GLUT-4) expression, and through decreasing the levels of NF-κB expression (Kanagasabapathy et al., 2012).

In another study, ergosterol derived from *P. ostreatus* also increased glucose uptake, GLUT 4 expression, and protein kinase C (PKC) and Akt activation in L6 cells (Xiong et al., 2018), whereas, in mice, ergosterol has been shown lower fasting blood glucose (FBG) and IR (Xiong et al., 2018). Wahyuni et al. (2017) observed that treatment with an ethanolic extract of *P. ostreatus* reduced pancreatic β-cell injury in Wistar mice. In another study, polysaccharides extracted from *P. ferulae* enhanced antioxidant capacity as well as HDL-C while lowering LDL-C levels in hyperglycemic rats (Choi et al., 2016a).

In various laboratory experiments in mouse models, the polysaccharides derived from the following mushroom cultures have demonstrated antidiabetic properties, including reductions in hyperglycemia, obesity, and hypertension. The fungi studied were *Agaricus brasiliensis* Fr.*, Agrocybe chaxingu* N.L Huang., *Catathelasma ventricosum* (Peck.) Singer*, G. lucidum, G. frondosa, Hericium erinaceus* (Bull.) Pers., *Lentinus edodes* (Berk.) Singer*., L. strigosus* Fr., *Phellinus linteus* (Berk. and M.A Curtis) Teng., *Pleurotus eryngii* (DC.) Quél*., P. florida* Singer*, P. ostreatus* (Jacq.) P. Kumm., *Pleurotus sajor-caju* (Fr.) Singer.*, P. tuber-regium, Schizophyllum commune* Fr.*, Sclerotium rolfsii* Sacc.*, Sparassis crispa* (Wulfen) Fr.*,* and *Tremella aurantialba* Bandoni and M. Zang., *T. fuciformis* Berk.*,* (Wang J. et al., 2016; Friedman, 2016; Gong et al., 2020; Jiang et al., 2020; Khursheed et al., 2020; Yin et al., 2020). The observed antidiabetic activity is likely to be as a result of the regulation of impaired glucose tolerance (the levels of glucose in the blood plasma) and the levels of various lipoproteins, which both improved host immunity and had an antioxidant effect. Furthermore, levels of insulin and intestinal microbiota were maintained, and changes in the structure of β-cells of the pancreatic glands were also observed (Friedman, 2016).

Zhu et al. (2016) demonstrated that polysaccharides derived from *G. lucidum* showed antidiabetic activity when fed to the mice over 4 weeks as part of their diet The antidiabetic activity was thought to occur through reduction of the fasting blood glucose levels and enhanced endothelium-dependent aortic relaxation, as well as increased levels of phosphoinositide 3-kinase (PI3K), phosphorylated Akt, eNOS, and nitric oxide and possibly linked to the involvement of the 29 PI3K/Akt/eNOS pathway.

In another study, Chen et al. (2018) evaluated the curative effect of an isolated heteropolysaccharide from *G. frondosa*, revealing that treatment with this polysaccharide could substantially increase glucose uptake and reduce insulin resistance in HepG2 cells, as well as improving glucose tolerance in T2DM induced mice. The mechanism was thought to be validation of the augmentation of the IRS1/PI3K-JNK insulin signalling pathway by polysaccharides at the protein level (Chen et al., 2018).

Natural polysaccharides have multiple mechanisms of action against DM, including increasing plasma insulin levels and decreasing pancreatic glucagon activity (reducing glycogen breakdown and gluconeogenesis) (He et al., 2006). Polysaccharides can also enhances insulin receptor sensitivity leading to reduced insulin resistance, can elevate glycogen synthesis in the liver and can improves the rate of glucose utilization and anti-inflammatory activity in peripheral tissues (Huang et al., 2014). Mushroom polysaccharides, therefore, have many potential advantages and are a promising topic of research underlining this mechanism of action (Meneguin et al., 2021).

Increased intracellular glucose levels stimulate the production of ROS in mitochondria and inhibit the enzyme GAPDH. As a consequence, four downstream tissue damage pathways are activated and result in: 1) increased polyol pathway flux; 2) increased non-enzymatic formation of AGEs and increased expression of receptors for AGEs (RAGEs); 3) activation of PKC; and 4) increased hexosamine pathway activity (Brownlee, 2005). In further analysis of the modes of action underlying the antidiabetic activity of mushroom polysaccharides, evidence gaps must be addressed before these compounds can be considered as therapies for the management of diabetes (Figure 7).

Edible mushroom polysaccharides have already demonstrated effectiveness as anti-hypoglycemic agents, and should be further researched as a potential source of hypoglycemic drugs. Furthermore, the consumption of mushrooms polysaccharides in the diet could be promoted with the aim of preventing diabetes (Gong et al., 2020).

## Peroxisome Proliferator Activated Receptor-γ (PPAR-γ) Pathway

The activation of PPAR-γ, which are ligand-activated transcription factors of the nuclear hormone receptor superfamily, causes insulin sensitization and improved glucose metabolism. The co-activator complex binds to a DNA sequence in the promoter region of target genes, prompting their transactivation or transrepression. PPAR-γ operate either as homodimers or heterodimers with the co-activator complex (Michalik and Wahli, 2006).

Hispidin isolated from *Phellinus linteus* was found to dramatically reduce ROS in H_2_O_2_-treated cells in a concentration-dependent manner. It also found to prevent apoptosis and to increase insulin secretion in H_2_O_2_-treated cells (Jang et al., 2010). Both these studies demonstrated that the antihyperglycemic action of hispidin occurred through avoidance of the ROS-mediated degeneration of β-cells. Hispidin also protect against the complications of diabetes by acting as a natural inhibitor of aldose reductase (Lee et al., 2010b; De Silva et al., 2012).

In another investigation, related observations has shown that, of the newly discovered polychlorinated compounds (chlorophyllins A-C) extracted from the *Phellinus ribis* (Schumach.) Quél*.*, chlorophellin C demonstrated considerable agonistic activity against PPAR-γ in NIH-3T3 cells (Lee IK. et al., 2008). Similar activity was reported for the polychlorinated substances extracted from *P. ribis* in Lee IK. et al. (2008). Another study reported that treatment with extracellular polysaccharides from *P. tuber* resulted in maintenance of a stable fatty acid composition and control of hyperlipidemia and obesity via the upregulation of hepatic PPAR-mRNA and protein levels, as well as exhibiting antihyperglycemic properties in obese-diabetic rats (Huang et al., 2014).

Additional investigation confirmed that extracellular polysaccharides were extracted from *Stropharia rugosoannulata* Farl. ex Murrill., lowered plasma glucose, total cholesterol, and triacylglycerol levels and decreasing lipid metabolism controlled by PPAR-γ in STZ-induced diabetic rats (Zhai et al., 2013).

The oral treatment (100 or 300 mg/kg, b. w.) with extracellular polysaccharides extracted from *G. frondosa* had anti-hyperlipidemic activity in high-fat diabetic rats by lowering BGL, TC, TG, and lipid profiles, fatty acid composition, and expression of liver PPAR-α. Likewise, oral supplementation with mushroom polysaccharide resulted in the simultaneous restoration of enzymatic antioxidants and increased HDL-C levels in high-fat diet plus STZ-induced rats (Lei et al., 2012; Huang et al., 2014).

## Polyol Pathway

The polyol pathway is thought to be involved in the development of diabetic degenerative syndromes including neuropathy, nephropathy, retinopathy, cataracts, and cardiovascular disease (de la Fuente and Manzanaro, 2003). Hyperglycemia may have a role in the pathogenesis of diabetes *via* increased flow of the aldose reductase-related polyol pathway and increased generation of AGEs. Aldose reductase inhibition, as well as protein glycation inhibition, may therefore be utilized in the treatment of diabetes. These activities were demonstrated by compounds extracted from the fruiting body of *P. linteus*. Davallialactone, methyldavallialactone, hypholomine B, interfungin A, and inoscavin A all demonstrated substantial inhibitory activity on rat retina (Lee et al., 2008a).

A meroterpenoid and five additional compounds (ganoleucin D, spiroapplanatumine K, spiroapplanatumine L, (+)-spirolingzhine A, and spirolingzhine D) were extracted from the fruiting body of the mushroom *Ganoderma leucocontextum* T.H. Li, W.Q. Deng, Sheng H. Wu D.M. Wang and H. P. Hu were found to have an inhibitory effect on aldose reductase and a significant inhibitory impact on HMGCoA reductase (HMGCR; 3-hydroxy-3-methyl-glutaryl-coenzyme A reductase) in molecular docking experiments (Zhang J. et al., 2017).

## Advanced Glycation End-Products (AGEs)

AGEs, often called glycotoxins, are a group of highly oxidant compounds associated with diabetes and other chronic diseases. In a non-enzymatic process, reducing sugars react with free amino groups in proteins, lipids, or nucleic acids to produce AGEs (Uribarri et al., 2010). The development of novel therapeutic agent(s) for the prevention of diabetic complications might result from the study of mushrooms with excellent AGE inhibitory activity. Yan et al. (2013) discovered that a polysaccharide extracted from the mycelium of *Ganoderma capense* (Lloyd) Teng. had anti-glycation and anti-radical properties in *in vitro* assays.

*Lignosus rhinocerus* sclerotia was powdered and subjected to a combination of cold water extraction and Sephadex^®^ G-50 (fine) gel filtration chromatography. The medium-molecular-weight (MMW) fraction showed anti-glycation activity in a human serum albumin-glucose system, through inhibition of the formation of N-(carboxymethyl)lysine, pentosidine, and AGE complexes (Yap et al., 2018). These results suggest that the MMW fraction of this species might be developed into a potent glycation inhibitor to prevent diabetic complications caused by AGE.

In another study, an alloxan-induced diabetic mice model was used to assess the antidiabetic activity of the dry matter of a culture broth (DMCB) of *Inonotus obliquus* (Ach. ex Pers.) Pilát. Oral treatment with the DMCB (500 and 1000 mg/kg for 21 days) resulted in significant decreases in blood glucose level, free fatty acids and LDL-C levels while improving insulin, HDL-C, and liver glycogen levels. CAT and GSH-Px activities also improved following DMCB treatment (Sun et al., 2008).

Wang et al. (2018b) investigated the therapeutic effect against diabetes of a polysaccharide isolated from *I. obliquus* (UIOPS) and its complex with chromium (III) (UIOPS-chromium (III) complex, UIOPC). UIOPC inhibited both the α-glucosidase and α-amylase enzymes, while both UIOPC and UIOPS showed anti-glycation antioxidant activities in hepatic L02 cells following H_2_O_2_-induced oxidative damage. Similarly, UIOPC regulated postprandial blood glucose levels, and both UIOPS and UIOPC showed considerable inhibition of three steps of AGE generation (Wang et al., 2018b).

Two independent studies, Meng et al. (2011) and Li (2011), reported that polysaccharides extracted from *Ganoderma lucidum* diminish myocardial collagen crosslinking in diabetic rats by reducing AGE levels and myocardial fibrosis as well as by increasing the activities of the antioxidant enzymes CAT and GSH-Px and enhancing their capacity to scavenge oxygen free radicals. Similarly, treatment with *G. lucidum* polysaccharides decreases oxygen free radical stimulus and the progression of diabetic myocardial fibrosis. This suggested that these polysaccharides might be employed to treat myocardial fibrosis (Meng et al., 2011). Furthermore, the *G. lucidum* polysaccharides mixed with metformin successfully prevent myocardial fibrosis in STZ-induced diabetic rats by boosting the activities of antioxidant enzymes and by downregulating myocardial connective tissue growth factor (CTGF). The impact of this combination therapy was greater than therapy with either of the drugs used separately at the same dosage (Qiao et al., 2014).

The oral administration of an extract from *Lactarius deterrimus* Gröger at a dosage of 60 mg/kg i. p. daily for 4 weeks to STZ-induced diabetic rats resulted in reductions in hyperglycemia, lipid levels, circulating levels of glycated haemoglobin, glycated serum protein, and AGEs (Mihailović et al., 2015). AGEs, which were assumed to be oxidative byproducts of diabetic hyperglycemia, are now becoming recognized as a possible risk factor for islet β-cell damage, peripheral insulin resistance (IR), and DM (Yap et al., 2018).

The experimental studies discussed above on the effects of mushrooms polysaccharides in the diet suggested that these compounds effectively lower AGE levels, restore the innate immune system and improves IR, providing new perspectives on diabetes pathology and treatment.

## Protein Kinase C (PKC) Pathway

PKC is thought to govern pancreatic activities in normal acinar cells, ductal cells, and islets, including in disease states like IR, DM, pancreatitis, and especially pancreatic ductal adenocarcinoma (Fleming and Storz, 2017). PKC also has a role in the progression of insulin tolerance and DM through the regulation of the proliferation and activity of β-cells, and also through insulin secretion and cell death. During pancreatitis, PKC is released following pancreatic injury and inflammation with trypsinogen activation and basolateral exocytosis (Theivendren et al., 2021).

Ergosterol isolated from *Pleurotus ostreatus* (Jacq.) P. Kumm., displayed antidiabetic activity in STZ (35 mg/kg, i. v.)-induced KK-Ay mice. Antidiabetic efficacy was observed at dosages of 1.0 or 2.0 g/kg body weight, orally administered over 4 weeks, and resulted in reduced blood glucose levels. In contrast, the expression of adipose tissue GLUT-4, levels of inflammatory biomarkers such as AMPK, Akt and the activity of the PKC pathway were all downregulated in the ergosterol-treated group (Xiong et al., 2018).

Oral treatment (8 weeks) of STZ-induced diabetic rats with polysaccharide (OMP) derived from *P. ostreatus*, resulted in a reduction in blood glucose levels. OMP treatment also increased the levels of p-AMPK relative to β-actin in the white adipose tissues, compared with those of the control group. Similarly, GLUT4 expression increased significantly in proportion to GAPDH in both muscle and adipose tissues in diabetic rats treated with OMP (Asrafuzzaman et al., 2018).

Polysaccharides extracted from *Antrodia camphorate* (M.Zang and C.H.Su) Sheng H. Wu, Ryvarden and T.T. Chang was found to significantly decrease platelet aggregation and PKC phosphorylation in platelets activated in phorbol-12, 13-dibutyrate (PDBu) at concentrations of 56—224 μg/ml. Furthermore, polysaccharides inhibited the PKC and Akt pathways during collagen-induced calcium (Ca^2+^) mobilization (Lu et al., 2014). The authors suggested that oral administration of these mushroom polysaccharides prevented several glucose-induced vascular dysfunctions and inhibited PKC activation, which suggests that mushroom polysaccharides might be an alternative source of antioxidants to treat hyperglycemia-induced oxidative stress.

## NF-κB Pathway

NF-κB is involved in the pathophysiology of DM. Persistent hyperglycemia stimulates the transcription factor NF-κB, which causes the production of a variety of cytokines, chemokines, and cell adhesion molecules (Suryavanshi and Kulkarni, 2017). The expression of NF-κB is a thoroughly researched intracellular pathway, and is affected by hyperglycemia, ROS, and oxidative stress. The pathway is also important in immunological and inflammatory responses, as well as in apoptosis. NF-κB controls the expression of a wide number of genes, including those that have been connected to DM (e.g., vascular endothelial growth factor (VEGF) and RAGE). Elevated levels of several products of those genes (e.g., VEGF, RAGE) controlled by NF-κB are linked to abnormal NF-κB regulation, and can cause a variety of chronic illnesses, including diabetes and atherosclerosis (Giacco and Brownlee, 2010).

Hu et al. (2017) found that the oral administration of polysaccharides isolated from *Auricularia auricular* (Gray) G.W. Martin., resulted in hypoglycemic and antidiabetic nephritis activity in STZ-induced diabetic rats at doses of 400 mg/kg for 4 weeks. The polysaccharides resulted in an 80.1% reduction in HbA1c levels. The polysaccharides also lowered the levels of TNF-α, IL-2, and IFN-γ with the activation of the NF-κB pathway.

A streptozotocin (STZ)-induced hyperglycemic rat model connected to the Nrf2 and NF-κB pathways was used to assess the hypoglycemic effect of extracts from *Tuber melanosporum* Vittad. A variety of biomarkers and inflammatory indicators were studied by the authors. The treatment with *T. melanosporum* (TE) polysaccharides decreased glucose levels and reduced oxidative stress by modulating SOD, CAT, vitamin-E and C in diabetic rats. The treatment also increased the gene expression of Nrf2 and NF-κB in rats, in comparison to the control group. The effects associated with the mRNA expression of genes in the inflammatory and oxidative stress pathways was significantly reduced following treatment with TE in diabetic rats (Zhang et al., 2018e).

Kanagasabapathy et al. (2012) discovered that polysaccharide-rich β-glucans (240 mg/kg body weight) from *Pleurotus sajor-caju* mushrooms could prevent hyperglycemia as well as hyperinsulinemia/IR in high-fat diet-induced mice. The authors also reported that polysaccharide-rich β-glucans increased the expression of the insulin-response glucose-transporter (GLUT-4) and also the genes encoding the antidiabetic and antiatherogenic hormone adiponectin (IL-6).

According to Mao et al. (2015), a polysaccharide isolated from *Grifola frondosa* showed an immunomodulatory effect in mice splenocytes through the MAPK and NF-κB signalling pathways. Another study reported that polysaccharides extracted from *Auricularia auricular-judae* (Bull.) Quél., exhibit anti-nephritic activity in STZ-induced diabetic rats through modulating oxidative stress as well as through the NF-κB pathway (Hu et al., 2017).

A related study reported that zinc-polysaccharides (MZPS) extracted from *Pleurotus djamor* mycelium reduced oxidative stress and protected against kidney and liver damage through the activation of enzymatic antioxidants (SOD, GSH, GSH-Px and CAT) in STZ-induced diabetic mice (Zhang et al., 2015a). The MZPS treatment also markedly reduced levels of MDA, alanine aminotransferase (ALT), aspartate aminotransferase (AST), blood urea nitrogen (BUN), creatinine, TC, LDL-C and HDL-C in liver, kidney and serum samples (Zhang et al., 2015a). These selected studies provide evidence for the antidiabetic properties of mushroom polysaccharides that might be inhibited by the NF-κB inflammatory pathway, which is proposed as a new target for the management of DM.

## p38 MAPK Pathway

The p38 MAPK pathway is activated in response to hyperglycemia and diabetes. Administration of insulin, as well as hyperglycemia, activates p38 MAPK in vascular smooth muscle cells (Wang S. et al., 2016). High hyperglycemia generates a fourfold increase in p38 MAPK in rat aortic vascular smooth muscle. This activation was observed in the glomeruli of STZ-induced diabetic rats, and was followed by increased phosphorylation of heat shock protein 25 (Hsp 25) by the down substrate of p38 MAPK (Denhez et al., 2019). Overall levels of JNK/SAPK and p38 MAPK are higher in the nerve tissue of individuals with T2DM diabetes (Evans et al., 2003).

An *in vitro* study was conducted using bioactive polysaccharides extracted from *Agaricus blazei* on LPS-induced RAW 264.7 cells. The polysaccharide reduced JNK, ERK, and p38 expression while increasing insulin levels in STZ-induced diabetic mice. These data clearly showed that polysaccharides are able to have a hypoglycemic impact through inhibition of pancreatic β-cell death *via* downregulation of the JNK/p38 pathway (Cheng et al., 2017).

Experiments have also shown that mushroom polysaccharides can alter endothelial dysfunction in an STZ diabetes-induced animal model. Polysaccharides extracted from *Ganoderma atrum* J.D. Zhao, L.W. Hsu and X.Q. Zhang., reduced FBG dramatically and improved plasma lipid profiles (Zhu et al., 2016). Furthermore, treatment with polysaccharides derived from *Grifola frondosa* significantly reduced GSK-3 (glycogen synthase kinase-3) expression, while significantly increased expression of insulin receptor, IRS1, PI3K, Akt, eNOS, and GLUT4 in the liver tissue of diabetic rats (Li et al., 2019).

Treatment with polysaccharides from *G. frondosa* greatly increased glucose metabolism and glycogen production in HepG2 cells. Similarly, insulin receptor was activated and Akt expression was elevated, while GSK-3 expression was reduced following polysaccharide treatment, as verified using Western blot experiments (Ma et al., 2014). Another study tested the effect of treatment with a methanolic extract of *Hericium erinaceus* (Bull.) Pers. in HFD-STZ diabetes-induced rats. At a dose of 100 mg/kg-b. w, *H. erinaceus* extract treatment decreased levels of BGL, TGs, and TC in the rats (Wang et al., 2005). From another investigation, oral administration of an alcoholic extract of *H. erinaceus* decreased BGL in STZ-induced diabetic rats. In the liver tissue of the rats, *H. erinaceus* extract lowered lipid levels and increased the activities of the enzymes CAT, SOD, GSH-Px and GSH, while levels of MDA were significantly reduced (Liang et al., 2013).

A detailed study by Cai et al. (2020) identified the polysaccharide HEP-C from *H. erinaceus*. The oral administration of this polysaccharide (at doses of 150 and 300 mg/kg/b.w) reduced FBG. On the other hand, polysaccharide treatment improved glucose tolerance, serum lipid metabolism and hepatic functions, elevated the activities of antioxidant enzyme, and prevented lipid peroxidation in STZ-induced diabetic rats. The hypolipidemic activity of the polysaccharide HEP-C was accompanied by stimulation of the phosphatidylinositol-3-kinase/protein kinase B (PI3K/Akt) signalling pathway (Cai et al., 2020). The treatment of alloxan-induced diabetic mice with polysaccharides extracted from *H. erinaceus* resulted in an anti-hyperglycemic effect through reductions in BGL and improvement in glucose tolerance levels (Chaiyasut and Sivamaruthi, 2017). Many studies report that P38 MAPK is abnormally expressed in the cardiovascular system in STZ-induced diabetic animals (Wang S. et al., 2016). The results discussed above suggested that mushroom polysaccharides can inhibit the p38 MAPK pathway through immunomodulation which can prevent the development of DM.

## Hexosamine Pathway

The hexosamine pathway, which is activated by ROS, oxidative stress, and suppression of GAPDH enzyme activity, results in cell death in arteries and nerves, and leads to nephropathy, neuropathy, and cardiovascular disorders (Volpe et al., 2018). An excessive flow of glucose or free fatty acids (FFAs) into a variety of cell types activates the hexosamine biosynthetic pathway, resulting in insulin resistance as well as the establishment of chronic complications of diabetes (Teo et al., 2010). In vascular endothelial cells, hyperglycemia results in a significant activation of the hexosamine pathway, which could be reversed using an electron transport inhibitor, a mitochondrial uncoupling agent (carbonyl cyanide chlorophenylhydrazone, CCCP), and increasing the synthesis of either uncoupling protein 1 or MnSOD (Rajamani and Essop, 2010).

Treatment with polysaccharides isolated from *Ganoderma lucidum* was found to increase wound angiogenesis and to improves delayed wound healing in STZ-induced T1DM mice by partly lowering cutaneous MnSOD nitration, p66Shc, and reducing oxidative stress in mitochondria (Tie et al., 2012).

In another study, polysaccharides extracted from *Phallus impudicus* L., were found to potentially promote epithelialization, granulation tissue contraction, and overall growth when given topically to full-thickness cutaneous wounds in STZ-induced diabetic rats. Furthermore, treatment with these polysaccharides decreased the values of circulating immune complexes increased the phagocytic activity of human blood cells (Vyacheslav et al., 2019).

There is evidence that the NF-κB, JNK/SAPK, p38 MAPK, and hexosamine pathways are stress-sensitive signalling systems that might be triggered in *in vitro* experiments by hyperglycemia and ROS (Chen et al., 2018). Chronic activation of these pathways has been linked to diabetic issues later in life (Mellitus, 2005). This seems to be an area that merits further investigation, as it might lead to new insights into the molecular aetiology of hyperglycemia as well as the identification of pharmaceutical targets for the treatment and/or prevention of the late consequences of diabetes (Singh et al., 2005). When polysaccharides from multiple dietary sources are combined, they may potentially be effect in the reduction of hyperglycemia, hyperlipidemia, low-level inflammation, and oxidative stress in diabetics, suggesting that oral intake of mushroom polysaccharides might be a potential option for the management of diabetes.

## Oxidative Stress and Insulin Resistance

Insulin resistance and reduced insulin production are both key aspects of T2DM pathogenesis (Scheen, 2004). Insulin resistance occurs several years before the development of T2DM, affects a huge portion of the population, and is complex (Mellitus, 2005).

An investigation by Laurino et al. (2017) into the antidiabetic properties of *Lentinula edodes* (Berk.) Pegler., demonstrated that treatment with an extract from this mushroom resulted in reduction of BGL in gestational diabetes-induced rats. This result indicates that extracts from *L. edodes* have a potential application as nutraceuticals in the diets of pregnant women with gestational diabetes (Laurino et al., 2017). Jia et al. (2009) found that oral treatment with *Ganoderma lucidium* polysaccharides for 30 days in diabetic rats resulted in reduced BGL and lipid peroxidation levels. Furthermore, the polysaccharide treatment restored the levels of insulin and antioxidants.

Treatment with a polysaccharide (IOPS) isolated from *Inonotus obliquus* significantly decreased body fat, FBG, and liver glycogen, as well as IR, in diabetic mice. Moreover, treatment with IOPS also promoted hepatic cholesterol transport, increased HDL-C levels, and lowered levels of TG, TC, and LDL-C. The hepatic antioxidant system in the liver was also elevated, as were the expression levels of p-Akt and GLUT4, following IOPS administration. These data suggested a putative antihyperglycemic mechanism involving Akt as well as PI3K phosphorylation and GLUT4 translocation (Wang et al., 2017). In another investigation, two triterpenoids, both with α,β-dimethyl and α,β-unsaturated δ-lactone side chains, were isolated from *I. obliquus*. In comparison to the positive control acarbose, treatment with these triterpenoids were found to be more effective in an α-glucosidase inhibition assay (Ying et al., 2014).

Stojkovic et al. (2019) evaluated the *in vitro* antidiabetic properties of extracts from six medicinal mushroom species, including *Agaricus blazei*, *Coprinus comatus* (O.F. Müll.) Pers., *Cordyceps militaris* (L.) Fr., *Inonotus obliquus, Morchella conica* Pers., and *Phellinus linteus*. In the comparative study on enzyme inhibition in *I. obliquus* extract exhibited the highest potential for the inhibition of α-amylase (Stojkovic et al., 2019).

Liu Y. et al. (2013) showed that polysaccharides (CVPs) isolated from *Catathelasma ventricosum* exhibited anti-hyperglycemic activities with significant reductions in BGL, TC, TGs, and MDA. On the other hand, treatment with CVPs significantly increased levels of HDL-C and antioxidant enzyme activity (Liu Y. et al., 2013). Liu et al. (2016b) suggested that polysaccharides from *C. ventricosum* showed anti-hyperglycemic activity in STZ-induced ICR mice at a dose of 100 mg/kg/day for 30 days, and resulted in a substantial reduction in BGL, TGs, TC, and LDL-C, and elevated levels of HDL-C (Liu et al., 2016b)*.*

Wu and Xu (2015) investigated the antidiabetic and antioxidant activities of eight medicinal mushroom species from China using an *in-vitro* aldose reductase (AR) inhibition assay and α-glycosidase tests. In this study, *Ganoderma lucidum* extract had the best dose-dependent activity against α-glycosidase, the best aldose reductase inhibitory capacity and the best DPPH free radical quenching effect of all the studied mushrooms. A growing number of studies have suggested that growth factors may play a role in the pathogenesis of diabetes complications, owing to the complicated process of disease development and the intricate linkages between oxidative stress and diabetic complications. On the other hand, the direct or underlying mechanisms responsible for the association between antioxidants and problems associated with diabetes, have yet to be established.

## Polysaccharide Activity Against Insulin and Fat Deposition by Activation of Autophagy

T2DM is caused by autophagy, a lysosomal breakdown mechanism. Autophagy also helps to lower BGL and preserve pancreatic islets from damage caused by high BGL levels (Stienstra et al., 2014). When Kunming mice were administered with an extract from *Pleurotus eryngii*, overall growth (weight), as well as levels of plasma insulin, TGs, TC, LDL-C, and blood glucose, were all reduced, while HDL-C and liver glycogen levels were increased (Chen L. et al., 2016). Another study found that supplementing the diets of diabetic rats with polysaccharides extracted from *Auricularia auricular* decreased weight gain and LDL levels (Lu et al., 2018). Shen et al. (2019) suggested that treatment with polysaccharides from *Auricularia judae* improved glucose absorption but decreased AGEs in HepG2 cells. In another study, polysaccharides derived from *Coprinus comatus* exhibited antioxidant and α-amylase inhibition activities in *in vitro* experiments (Cao et al., 2019).

Zhao et al. (2018) examined the antihyperglycemic activity of polysaccharides from *Cordyceps militaris*. Treatment at 100 mg/kg resulted in lower levels of lipid peroxidation, BGL, TG, TC, LDL-C, FBG, HOMA-IR, VLDL-C and IR, but promoted antioxidant enzyme activities in diabetic induced mice. Similarly, treatment with a polysaccharide from *Cordyceps taii* Z.Q. Liang and A.Y. reduced BGL and hyperlipidemia in diabetic induced mice. In addition, polysaccharide treatment reduced TG, TC levels, while HDL-C levels increased (Xiao et al., 2012).

Yoshioka et al. (2017) found that adenosine extracted from *Grifola gargal* Singer., increased glucose absorption in skeletal muscle cells through the AMPK and P13k signalling pathways in skeletal muscle cells. In another study, β-glucans isolated from *Lentinula edodes* exhibited an antidiabetic effect with decreased BGL levels and increased enzymatic antioxidants levels when administered to diabetic mice (Afiati et al., 2019). Polysaccharides extracted from *Ganoderm atrum* showed hypoglycemic activity with reduction of ALT, AST and hepatic glycogen levels. Furthermore, polysaccharide treatment significantly downregulated the expression of PPAR-γ and increased those of GLUT-4 transporters in liver tissues of diabetic rats (Zhu et al., 2016).

Oh et al. (2010) discovered that submerged-broth culture of *Agaricus blazei* showed antidiabetic activity in rats. Similarly, a polysaccharide (PLP) isolated from *Phellinus linteus* exhibited an antidiabetic effect when administered to diabetic rats. The oral administration of PLP lowered FBG levels and improved glucose tolerance through modification of the hepatic phospholipid metabolism as well as restoration of insulin signalling transduction *via* the FOXO signalling pathway (Chen H. et al., 2016).

A polysaccharide (PLP) from *Phellinus linteus* inhibited BGL in diabetic mice. The PLP treatment decreased lymphocyte infiltration in pancreatic islets. Furthermore, PLP inhibited the production of inflammatory markers such as interleukins, IFN-γ, and TNF-α as well as reducing the numbers of macrophages and β-Cell lipid-induced toxicity (Kim et al., 2010a). The studies discussed here suggested that certain mushroom polysaccharides are effective antioxidants and may help to reduce oxidative destruction and oxidative stress (both of which are related to diabetes) in *in vivo* conditions when administered in food, but may also have an influence on obesity and other complications.

## Activation of Stress-Sensitive Signalling Systems, Insulin Receptor Substrate Serine Phosphorylation, and Insulin Resistance

ROS and oxidative stress activate many serine kinase pathways. Insulin receptor substrate-2 (IRS-2), protein kinase-B (PKB), the Foxo protein (FOXO), and the p85β regulatory subunit of PI-3 kinase are among the many molecules involved in the insulin signaling pathway. These molecules have attracted much research interest, as a consequence of their dysfunction leads to IR in *in vivo* (Brownlee, 2001 **)**. The identification of signaling deficiency, as well as the understanding of the complicated interactions between the many factors that modulate insulin sensitivity, is essential for the development of innovative and more precise antidiabetic drugs (Saini, 2010).

Treatment with the polysaccharide GFP-N derived from *Grifola frondosa* improves IR in *in vivo* by regulating JNK and IRS1/PI3K signaling and GFP-N enhanced hepatic IR, lowering blood glucose levels, and protecting the kidneys and liver from damage (Chen et al., 2019c). According to a randomized double-blind study by Hsu et al. (2007), treatment of human patients with T2DM with a combination of an extract from the mushroom *A. blazei* together with gliclazide and metformin for 12 weeks improved IR (Hsu et al., 2007). Furthermore, Yang et al. (2018) found administration of *Lentinula edodes* polysaccharides enhanced insulin production and lowered insulin resistance, while also improving gut microbiota dysbiosis, in insulin-deficient type 2 diabetic mice. A further study reported that treatment with polysaccharides derived from *G. frondosa*, *L. edodes*, *Phellinus linteus*, and *Ophiocordyceps sinensis* effectively reduced FBG and IR and improved glucose tolerance in STZ-induced diabetic mice. The oral treatment of polysaccharides has been found to effectively increase the expression of IRS1 and PI3K mRNAs while lowering the expression of JNK1/2 mRNA (Khursheed et al., 2020). According to these reports, mushroom polysaccharides have been found to control hyperglycemia, reverse IR and protect against diabetic complications. As a result treatment with these polysaccharides is seen as a promising alternative for the management of diabetic problems.

## Mechanism-Based Therapeutic Approaches

Much research into mushroom-derived products has shown that the use of these compounds in the treatment of diabetes and its complications can be beneficial. Summarized in Supplementary Table S2 are the most recent findings on the effects of mushroom polysaccharides on hyperglycemia and their use as a diabetic complication adjuvant therapy. The study of mushroom polysaccharides has primarily focused on lipid metabolism and diabetic complications caused by oxidative stress. Because the current diabetes treatments do not prevent diabetic complications, one goal of drug discovery is to uncover therapies that target new metabolic pathways. New mechanism-based prospective treatments include poly (ADP-ribose), PARP, SOD/CAT mimetics, and transketolase activators (benfotiamine) (Du et al., 2008). A particular PARP inhibitor reduces reactive hypoglycemia activation of PKC, NF-κB, intracellular AGE production, as well as the hexosamine pathway in cultured artery endothelial cells. PARP inhibition reduces nephropathy and sensory neuropathy in diabetic animals by preventing arterial endothelial cell damage and podocyte death (Szabó et al., 2006).

The next group of mechanism-based treatments is SOD/CAT mimetics. Excess superoxide directly inhibits essential anti-atherosclerosis endothelial enzymes, bypassing the five threatening pathways connected to metabolite-induced diabetic complications. eNOS and prostacyclin synthase are both inhibited in diabetic humans and other diabetic animals (Giacco and Brownlee, 2010). In addition to restricting the activation of the mechanisms indicated above, reducing the amount of superoxide directly is crucial to prevent oxidative inactivation of these critical enzymes. Antioxidants that neutralize ROS one-for-one are unlikely to be useful under these conditions because superoxide overproduction resulting from hyperglycemia is a continuous process. Based on the evidence of the favorable benefits of overexpression of antioxidant enzymes in mouse models, a novel form of antioxidant, for example, a catalytic antioxidant such as a SOD/CAT mimic, is needed (Salvemini et al., 1999).

We hypothesized in this review that mushroom polysaccharides could act as antioxidants, with ROS being inhibited by uncoupling protein-1, an electron transport chain complex II inhibitor, and manganese SOD, an oxidative phosphorylation uncoupler. By normalizing mitochondrial ROS concentrations, antioxidant agents decrease glucose-induced PKC, AGEs, sorbitol accumulation, and NF-kB activation (Nishikawa et al., 2000).

Interestingly, Huang et al. (2014) reported that extracellular polysaccharides derived from *Pleurotus tuber* maintained a steady fatty acid composition and regulated hyperlipidemia and obesity in diet/STZ-induced diabetic rats. This occurs *via* the upregulation of hepatic PPAR-mRNA and protein levels while also having an anti-hyperglycemic effect. The polysaccharides extracted from *Auricularia auricular* have anti-nephritic effects when administered in STZ-induced diabetic rats regulating oxidative stress as well as the NF-κB pathway (Hu et al., 2017). Polysaccharides from *Ganoderma lucidum* reduce myocardial collagen crosslinking in diabetic rats by lowering AGE levels and increasing the activities of antioxidant enzymes, suggesting that they could be used to treat myocardial fibrosis. These *G. lucidum* polysaccharides can also increase the ability to scavenge ROS, which are injurious to myocardium, reduce AGEs and myocardial fibrosis, and delay the progression of diabetic myocardial fibrosis (Li, 2011; Meng et al., 2011).

Some studies have reported that *Ganoderma lucidum* polysaccharides successfully inhibit myocardial fibrosis when administered to diabetic rats in combination with metformin, through enhancing antioxidant enzyme activity (Qiao et al., 2014). Similarly, *G. lucidum* polysaccharides have been reported to promote wound angiogenesis and accelerate wound healing in T1DM-induced mice (through partial suppression of cutaneous MnSOD nitration, p66Shc, and increased oxidative stress in mitochondria (Tie et al., 2012). *G. lucidum* polysaccharides inhibited the expression of adipogenic transcription factors PPAR-γ, sterol regulatory binding element protein-1c (SREBBP-1c), and CCAAT/enhancer-binding protein- (C/EBP-), as well as fatty acid synthase (FAS), acyl-CoA synthetase-1 (ACS1), fatty acid transport protein-1 (FATP1) and perilipin, fatty binding protein-1 (FBP-1) involved in lipid metabolism in 3T3-L1 cells (Thyagarajan-Sahu et al., 2011). An acidic polysaccharide (TAP) derived from *Tremella aurantia* Schwein showed an antidiabetic effect through enhancement in the activities of glucokinase, hexokinase, and glucose-6-phosphate dehydrogenase, as well as a reduction in the activity of glucose-6-phosphatase, in the livers of induced diabetic mice (Kiho et al., 2000).

Lei et al. (2013) discovered that a β-glucan was extracted from *Grifola. frondosa* protect mouse pancreatic cells from damage by oxidative stress. Extracellular polysaccharides (at 20 mg/kg) of three varieties of *Pleurotus tuber-regium* mushrooms were administered orally to high-fat diabetic rats, and both obesity and diabetes were reduced. Polysaccharides appear to act as PPAR agonists, enhancing lipid absorption, activation, and catabolism through transcriptional modifications in genes that control these processes, resulting in lower blood triglyceride and higher HDL levels (Huang et al., 2014).

From the many *in vivo* and *in vitro* studies examined in this review, oral administration of mushroom polysaccharides appears to reduce hyperglycemia and hyperlipidemia *via* numerous underlying molecular mechanisms. All of these studies showed that the antioxidative properties of these polysaccharides can help to improves the antioxidant system and reduce oxidative stress, suggesting that polysaccharides could be employed as an alternative therapy for diabetes-related hyperglycemia with oxidative stress.

We investigated the current state of knowledge on oxidative stress molecular pathways and the role of mushroom polysaccharides as antioxidants in downstream tissue damage processes. Given the link between oxidative stress and diabetes, several novel diabetic treatments approaches targeting oxidative stress reduction have been recently tested and approved, including treatment with enzymatic antioxidant-like mimics (SOD/CAT/GPx mimetics), vitamins (A, C, E), β-carotene, flavonoids, selenium, zinc, NAC, and CoQ10. In some clinical trials, antioxidant treatment has been demonstrated to have no effect on diabetes or its consequences. This may be due to the poor solubility of the antioxidant, its non-selectivity, or possibly its instability in conventional delivery systems. This could be overcome or improved by novel antioxidant delivery systems, following further safety, efficacy and pharmacokinetic research (Folli et al., 2011; Zhang et al., 2020).

## Conclusion and Future Research Implications

The antioxidant effects of mushroom polysaccharides and their potential role in the treatment of T2DM have attracted a large amount of research attention. Preclinical investigations suggested that hyperglycemia and perhaps elevated FFA levels (alone or in combination) stimulate the creation of ROS and RNS, resulting in increased oxidative stress in a variety of tissues. In the absence of an appropriate compensatory response from the cell’s natural antioxidant network, the cellular machinery becomes overwhelmed, causing redox imbalance and worsening the disease. ROS damage the cells not only by oxidizing DNA, proteins, and lipids, but also by activate stress-sensitive intracellular signalling pathways such as the NF-κB, p38 MAPK, JNK/SAPK, hexosamine, PKC, AGE/RAGE, sorbitol, and other pathways. This results in the synthesis of several gene products that are implicated in the aetiology of the chronic effects of diabetes. The results from different studies conducted suggested that activation of the same or related stress pathways causes insulin resistance and decreased insulin production. For this reason, we believe that there are links between hyperglycemia and FFA-induced augmentations in ROS and oxidative stress, activation of stress-sensitive pathways, insulin resistance, β-cell dysfunction and the development of diabetes complications.

Multiple observations have been made by different authors suggesting that polysaccharides act as antioxidants, reducing the production of ROS and a targeting the pathways and chemicals that lead to diabetic complications, and treatment with antioxidants may be considered as a potentially important therapy for the treatment of diabetic patients. Further research is, however, needed to understand the mechanisms behind the reduction in ROS levels as well as how blocking ROS-induced stress pathways can improves the action or secretion of insulin, and the post-translational modifications of gene products formed from the various mitochondrial ROS activated signalling pathways. The majority of studies recommend that larger clinical trials examine the efficacy of mushroom polysaccharides as an antioxidant treatment for diabetes.

Much information is available on the processes that lead to oxidative stress in diabetes. The findings discussed in this review are likely to lead to the development and testing of new antioxidant agents, such as SOD and CAT mimetics, which could, at least in the short term, slow the progression of diabetic complications. The various studies and discussions on mushroom polysaccharides that are reviewed in this manuscript suggest that mushroom polysaccharides with antioxidant activity should be further researched, and support their development as therapeutic agents in the treatment of diabetes.
