# Supplementary figures and images for: The Antioxidant Properties of Mushroom Polysaccharides can Potentially Mitigate Oxidative Stress, Beta-Cell Dysfunction and Insulin Resistance
Source: Front Pharmacol. 2022 May 5;13:874474. doi: 10.3389/fphar.2022.874474 (PMC9117613; doi:10.3389/fphar.2022.874474)

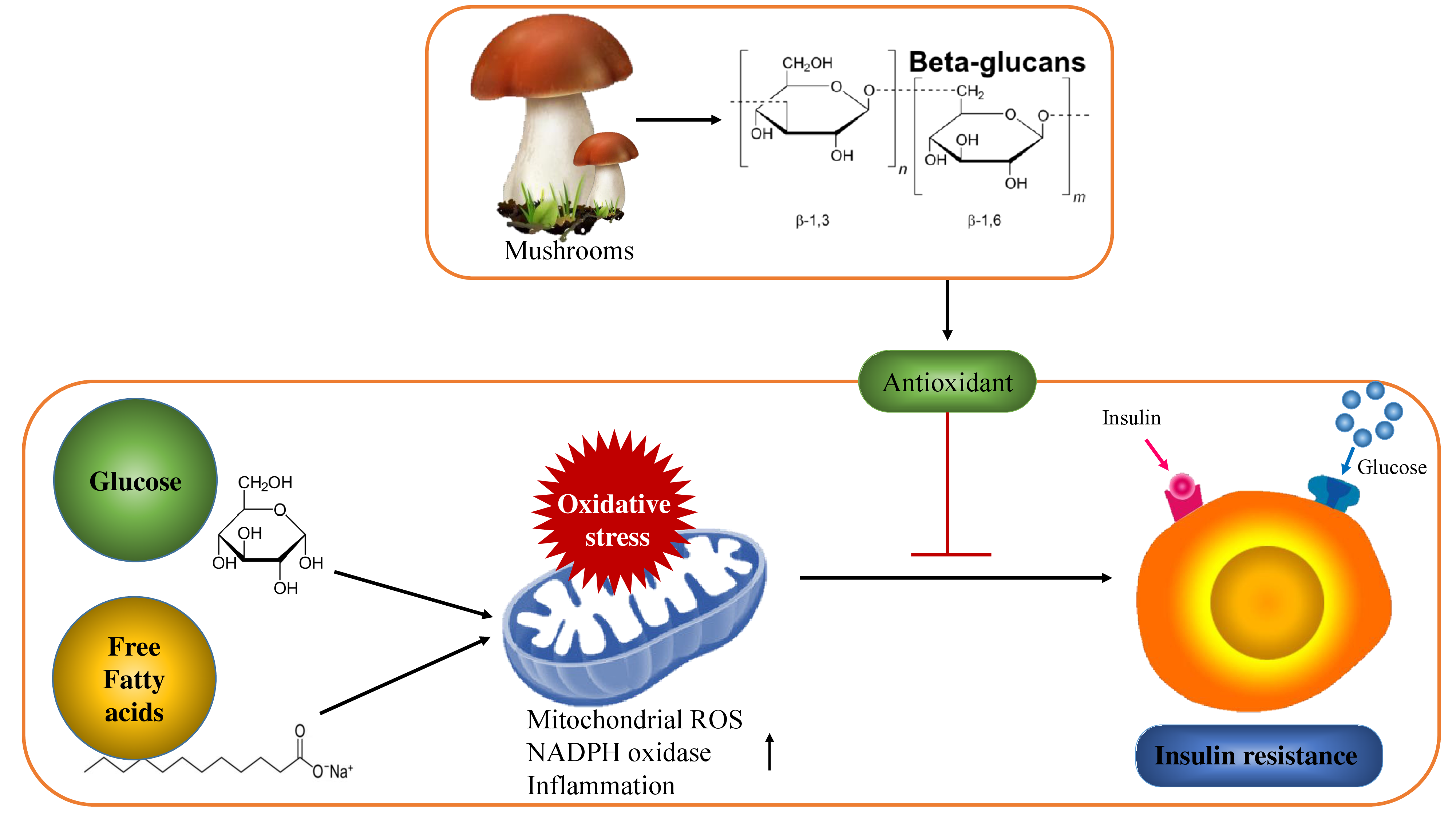

Supplement: Supplementary file 1 [file Image1.TIFF]
