# Supplementary material for: The Antioxidant Properties of Mushroom Polysaccharides can Potentially Mitigate Oxidative Stress, Beta-Cell Dysfunction and Insulin Resistance
Source: Front Pharmacol. 2022 May 5;13:874474. doi: 10.3389/fphar.2022.874474 (PMC9117613; doi:10.3389/fphar.2022.874474)
Supplement: Supplementary file 2 [file Table1.docx]

**Table 1: Antioxidant activity (in vitro and in vivo) of mushrooms and identified compounds**

| **Scientific names** | **Experiments** | **Dose/concentrations** | **Key results** | **Compounds** | **References** |
| --- | --- | --- | --- | --- | --- |
| *Agaricus bisporus* (J.E. Lange) Imbach | *In vitro:* RPA, O_2_^-^, ^•^OH and DPPH, and H_2_O_2_.  *In vivo:* SOD, CAT, GSH-Px, | *In vitro*: 0.05–0.2 mg/mL; *in vivo* : 300, 600 and 1200 mg/kg body weight per day, 30 consective days | - The antioxidant enzymatic activity in mice's serums, livers, and hearts significantly greatly boosted *in vivo* using ethanolic extract. - The phenolic content of the extract indicated 6.18 mg of gallic acid equivalents per gram dry weight. | Gallic acid, rotocatechuic acid, catechin, caffeic acid, ferulic acid and myricetin. Polysaccharides consisted mainly of (1 → 6)-β-D-glucans. | Kozarski et al., 2011; Kozarski et al., 2014; Reis et al., 2012; Liu et al., 2013 |
| *Agaricus brasiliensis* Fr. | DPPH, Chelating assay, TBARS, RPA | 0.1 to 20 mg/ml, 100 ml | - *G. lucidum, A. brasiliensis,* and *A. bisporus* polysaccharide demonstrated antioxidant activity with EC_50_ values of 7.07 mg/ml, 13.25 mg/ml, and >20 mg/ml, respectively. - In *G. lucidum*, ferrous ion chelating activity varied from 0.59 mg/ml to 7.80 mg/ml, whereas in A. bisporus, it was 0.59 mg/ml to 7.80 mg/ml. - The extracts' EC_50_ values varied from 0.47 to 14.83 mg/ml in the reducing power experiment. The EC50 values of the ion exchange and reducing power capabilities were found to be proportional to the quantity of total glucans with in extracts. | α- and β-glucans | Kozarski et al., 2011; Kozarski et al., 2014; Reis et al., 2012; |
| *Agrocybe aegerita* (V. Brig.) Singer “[*Cyclocybe aegerita* (V. Brig.) Vizzini]” | DPPH, inhibition of β-carotene bleaching assay, TBARS | 30-270 μL | - DPPH (EC_50_=7.23 mg/mL). - The carotene/linoleate (EC_50_ 6.11 mg/mL) - EC_50_ for the ferricyanide/Prussian blue assay (2.66 mg/ml ) - EC_50_ for the TBARS assays (0.39 mg/mL). | γ- tocopherol, β-tocopherol, δ-tocopherol and α-tocopherol | Petrović et al., 2015 |
| *Auricularia auricularis*  (Gray) G.W. Martin  “[*Auricularia auricula-judae* (Bull.) Quél.,]” | DPPH, TBARS, Conjugated Diene method, ^•^OH, Ferrous ion chelating assay | 5.0 mg/mL | - At 1.0-5.0 mg/mL, the TBARS test revealed 38.6 - 74.6%. - Methanolic extracts from red, jin, and snow ears mushrooms showed high antioxidant activity in conjugated diene reduction abilities and DPPH tests at 5.0 mg/mL. - Total antioxidant components in black, red, jin, snow, and silver ears are 15.69, 30.09, 27.83, 49.17, and 31.70 mg/g, respectively. | Ascorbic acid, tocopherols, total phenols, β -carotene | Mau et al., 2001 |
| *Auricularia cornea* Ehrenb. | DPPH, O_2_^-^, ^•^OH, nitrite assays |  | - *Auricularia cornea* var. Li polysaccharides exhibited a slightly greater hydroxyl radical scavenging capacity than *Auricularia auricula* polysaccharides, despite both having similar superoxide anion radical scavenging activity, which was stronger than Auricularia polytricha polysaccharides. - All three polysaccharides demonstrated significant DPPH radical scavenging action, with *Auricularia cornea* var. Li polysaccharides being the most effective, with an inhibition percentage of 80%. | polysaccharide | Luo et al., 2018 |
| *Auricularia polytricha* (Mont.) Sacc.  “[*Auricularia nigricans* (Sw.) Birkebak, Looney & Sánchez-García]” | ^•^OH, O_2_^-^, DPPH and nitrite assays |  | - *A. polytricha* demonstrated chemical free radical scavenging capacity in a dose-dependent approach in all scavenging assays, with lower EC_50_ values. Secondary metabolites including phenol and flavonoids were found in abundance in the extract. |  | Packialakshmi and Sudha, 2016 |
| *Auricularia mesenterica*  (Dicks.) Pers. | DPPH, β-carotene, ABTS, ascorbic acid, lycopene, TPC and TFC | 100 - 300 μg/mL | - The hydro alcoholic extract of *A. delicata* has the greatest phenol when compared to *A. mesenterica*. - Furthermore, the extract contained a high concentration of favonoids and ascorbic acids, two well-known chemical free radical scavenging components found in mushrooms. - The effectiveness of infusions, decoctions, and hydro alcoholic extracts to scavenge free radicals was dosage dependent. | Phenols, flavonoids and ascorbic acid | Ghosh et al., 2021 |
| *Auricularia fuscosuccinea* (Mont.) Henn. | *In vitro*: DPPH, RPA, TPC, TFC, ferrous ion chelating, total chelating assay.  *In vivo*: SOD, GPx | 0.1 to 3.5 mg/ml | - *A. polytricha* and *Tremella fuciformis*, the white variety of A. fuscosuccinea, had the greatest total phenolic [7.88 mg gallic acid equivalents (GAE)/g] as well as total flavonoid [1.60 mg quercetin equivalents (QE)/g]. - Every one of the methanol extracts investigated exhibited the lowest EC_50_ value on lowering g power (0.305 mg/ml) and scavenging action on DPPH radical (0.150 mg/ml), as well as the highest total sugars [44.73 mg dextrose equivalents (DEX)/g] and the lowest EC_50_ value on reducing g power (0.305 mg/ml). This white variety of *A. fuscosuccinea* methanolic extract showed the greatest antioxidant potential and superoxide dismutase activity (2.10 U/mg). |  | Lin et al., 2013 |
| *Agrocybe cylindracea* (DC.) Maire  “[*Cyclocybe parasitica (G. Stev.) Vizzini*]”*,* | RPA, DPPH, ^•^OH, ferrous ion chelating assays |  | - At 20 mg/ml^-1^, chemical free radical scavenging activity in hot-water extracts of fruit bodies, mycelia, and filtrate was 63.6%, 81.6 %, and 56.8%. - The EC_50_ values for reducing power for fruit bodies, mycelia, and filtrate were 2.72, 3.97, and 3.09 mg/ml^-1^, respectively, whereas the EC50 values for DPPH radical scavenging abilities were 0.62, 1.66, and 0.82 mg/ml^-1^. - Fruit bodies, mycelia, as well as filtrate had hydroxyl radical scavenging capabilities of 80.1 percent, 57.0 %, and 54.3 %, correspondingly, at 20 mg/ml^-1^. - In respect of EC_50_ values for chelating ferrous ions, the hot-water extract using filtrate exceeded the mycelia extract. Total phenols, which varied from 23.74 to 30.16 mg/g^-1^, were the most common found in nature antiradical component discovered in hot-water extracts. | Total phenols, ascorbic acid, β-carotene, and α-tocopherols | Tsai et al., 2006; Sevindik et al., 2018 |
| *Amanita rubescens*  Pers. | TPC, DPPH | 25 µL | - In a concentration-dependent manner, every one of the species proved capable of scavenging DPPH. The antiradical capacity of *B. edulis* was determined to be the highest. | Malic, Oxalic, Citric, Ketoglutaric, Quinic Succinic, Fumaric, Shikimic | Ribeiro et al., 2008 |
| *Agaricus arvensis*  Schaeff [*Agaricus osecanus* Pilát] and *A.bitorquis, A. campestris*  “[*Agaricus bisporus* (J.E. Lange) Imbach,]” and *A. silvaticus, A. blazei,* | DPPH, TPC assays | 0, 2, 4, 6, 8, 10 and 12 mg/mL | - The antiradical activity of the extracts against the DPPH radical was as follows: Brown > *A. bitorquis* > *A. arvensis* > *A. brasiliensis* White > *A. bisporus* > *A. campestris* > *A. silvaticus* with EC_50_ values for *A. bisporus* were determined to be the lowest. | lactic, malic, acetic, citric, formic, oxalic fumaric, malonic, and succinic acids | Gąsecka et al., 2018 |
| *Armillariella mellea*  (Vahl) P. Karst.  “[*Armillaria novae-zelandiae* (G. Stev.) Boesew.,]” | DPPH, antioxidant power assay, RPA, O_2_^-^, ferrous ion chelating assay | 0–20 mg/mL | - Low EC_50_ values (less than 10 mg/mL) reveal the antiradical properties of methanolic extracts using dried mycelia (MEM) and mycelia-free broth (MEB) and hot water extracts with dried mycelia (HWEM) generated by A. mellea submerged cultures. | Total phenolics, ascorbic acid, β-carotene, and α-tocopherols | Lung et al., 2011 |
| *Agaricus silvicola*  (Vittad.) Peck “[*Agaricus silvicolae-similis* Bohus & Locsmándi], *Agaricus silvaticus*  Schaeff*, Agaricus romagnesii*  Wasser “[*Agaricus bresadolanus* Bohus]” | DPPH, RPA, β-carotene bleaching and TBARS assays | 2.5 - 0.3 ml | - Antiradical activity was discovered in all of the species. | ascorbic and gallic acids | Barros et al., 2008 |
| *Antrodia camphorata*  (M. Zang & C.H. Su) Sheng H. Wu, Ryvarden & T.T. Chang  “[*Taiwanofungus camphoratus* (M. Zang & C.H. Su) Sheng H. Wu, Z.H. Yu, Y.C. Dai & C.H. Su]” | DPPH, stable nitrogen-centered free radical, plasma transaminase, GT, GPx, SOD, CAT, LPO | 100-µM | - An extract of *A. camphorata* decreased nonenzymatic iron-induced lipid peroxidation in rat brain homogenates with an IC_50_ value of around 3.1 mg/mL. - Also scavenged was the stable free radical DPPH. The dosage of *A. camphorata* extract required to achieve a 0.20 reduction in DPPH absorbance would have been about 31 (0.7 g/mL). - Furthermore, in a dose-dependent manner (250-1250 mg/kg), an A. camphorata extract improved the CCl_4_-induced rise in hepatic glutathione peroxidase, reductase, as well as superoxide dismutase activities. |  | Hsiao et al., 2003 |
| *Boletus edulis*  Bull. | ABTS, DPPH, FRAP |  | - Antiradical activity of processed for intake mushrooms was tested against ABTS (4.9–36.5 mmol TE), DPPH (7.8–21.3 mmol TE), and the FRAP assay (15.0–28.1 mmol Fe^2+^). | Total phenol, total flavonoid, Vitamin C and L-ascorbic acid, Tocopherols, β-carotene and lycopene contents, | Jaworska et al., 2015 |
| *Boletus badius*  (Fr.) Fr.  “[Picipes badius (Pers.) Zmitr. & Kovalenko]” | TAC, RPA, O_2_^-^, ferrous ion chelating assays | 25, 50, 100, 200, 400, 600 mg/mL | - Methanolic extracts of *Verpa conica*, *Boletus badius*, and *Russula delica* were shown to be more effective in scavenging DPPH as well as O_2_ chelating tests. | Total phenolics, β-carotene and α-tocopherol | Elmastas et al., 2007 |
| *Cantharellus lutescens* (Pers.) Fr. | Peroxidation of phospholiposomes, ^•^OH, H_2_O_2_, TAC, | 100 µl | - The linoleic acid test was performed to assess antiradical activity after 30 days of storage, and almost all of the samples demonstrated moderate or medium antioxidant activity, with the exception of canned Terfezia, Picoa, and Hydnum. | Trolox, α-tocopherol and propyl gallate | Murcia et al., 2002 |
| *Cantharellus clavatus* (Pers.) Fr. | RPA, DPPH, LPO | 5-25 µL | - *Cantharellus cibarius* was shown to have free radical scavenging action in testing. | β-carotene, ascorbic acid, and tannic acid, gallic acid, and α-tocopherols, protocatacheuic and gentisic acids | Puttaraju et al., 2006 |
| *Cantharellus cibarius* Fr. | DPPH, TAS, TOS, OSI | 50µL | - TAS, TOS, and OSI values were measured using extracts of C. cibarius mushroom in ethanol (EtOH), methanol (MeOH), and dichloromethane (DCM). The mushroom's TAS value was discovered to be 5.268 ± 0.059 mmol/L, its TOS value was 6.380 ± 0.256 mol/L, and its OSI value was 0.121 ± 0.005. - In a 1 mg/mL extract concentration of mushroom extract, DPPH free radical activity was found as EtOH extract 70.52 ± 0.50, MeOH extract 64.34 ± 1.54, and DCM extract 61.72 ± 0.59. |  | Tekeli et al., 2008; Sevindik, 2019 |
| *Cordyceps sinensis* (Berk.) Sacc.  “[*Ophiocordyceps sinensis* (Berk.) G.H. Sung, J.M. Sung, Hywel-Jones & Spatafora]” | SOD, ^•^OH, LPO | 0.1–5 mg/mL in macrophage culture | - The oxygen free radical scavenging activity of the extracts examined in SOD, HO, LPO assays found that greatest effect in the hot water extract. - The hot water extract also had a substantial inhibitory impact on lipid peroxidation in the medium and consequent cholesteryl ester buildup in macrophages when LDL has been incubated alongside macrophages in the presence of CuCl2 (1 mM). |  | Yamaguchi et al. 2000 |
| *Calvatia gigantea*  (Batsch) Lloyd | DPPH, TPC, total TFC, ascorbic acids |  | - The greatest total phenolic and flavonoid values were found in Leccinum scabrum (9.24 and 0.77 mg/g, correspondingly). - *Calvatia gigantea* exhibited the highest content of ascorbic acid as well as antiradical activity (108.11 mg/g). | Vanillic, Gallic, protocatechuic, Catechnin, t-Cynnamic, | Gąsecka et al., 2018 |
| *Cerrena unicolor*  (Bull.) Murrill | TAS, TOS, OSI, ABTS, DPPH | 6.25 to 800 μg/mL | - *C. unicolor* has been found to have antiradical properties. chemiluminometric study of these materials' chemical free radical scavenging properties and evaluation of the scavenging influence on ABTS and DPPH reduction rates were high. |  | Jaszek et al., 2013; Sevindik, 2019 |
| *Coprinus comatus*  (O.F. Müll.) Pers. | RPA, peroxidation of linoleic acid, DPPH, ^•^OH, O_2_^-^ | 1–10 mg/mL | - Ethanolic extract from stipe showed high total antioxidant activity (80.6%), superior DPPH radical scavenging capacity (57.9% at 1 mg/mL) and hydroxyl radical scavenging ability (57.4–61.3 percent at 5 mg/mL), and moderate superoxide radical scavenging ability (46.3–47.0%, at 20 mg/mL), and moderate superoxide radical scavenging ability (46.3–47.0 % at 20 mg/mL) | total phenols, total flavonoid, tocopherols, ascorbic acid and polysaccharide | Li et al., 2010 |
| *Dictyophora indusiata*  (Vent.) Desv.  “[*Dictyophora duplicata var. obliterata* Malençon]" | RPA, DPPH, ^•^OH, ferrous ion chelating, O_2_^-^ assays | 0.1 ml | - At a concentration of 1.0 mg/ml, water extract (WE) possesses a 97.35 % chemical free radical scavenging activity on DPPH radical. The reducing power of WE was modest (1.22 at 2 mg/ml). Conversely, at 2 mg/ml, the WE showed a 52.28 percent hydroxyl radical scavenging efficacy and a 48.64 percent superoxide anion scavenging activity. |  | O Oyetay et al., 2009 |
| *Flammulina velutipes*  (Curtis) Singer | DPPH, FRAP, TPC assay | 2-10 mg/ml | - The water extract had the greatest EC_50_ value (9.2 ± 0.18 mM Fe^2+^/g extract). Although the result was lower than that of ascorbic acid at 10 mg/ml (60.50 ± 1.32%), this extract had the maximum scavenging activity (57.53 ± 1.18%). | poly phenolics | Slawinska et al., 2013 |
| *Inonotus obliquus*  (Ach. ex Pers.) Pilát | DPPH, O_2_^-^, H_2_O_2_ | 50 mg/mL | - This study findings suggest that perhaps the polyphenolic extract preserved these cells from oxidative stress caused by hydrogen peroxide. | polyphenolics | Cui et al., 2005 |
| *Ganoderma lucidum*  (Curtis) P. Karst*.*  “[*Ganoderma orbiforme* (Fr.) Ryvarden]” | DPPH, TAC, H_2_O_2_, ABTS | 50, 100, 250, 500 and 1000µg | - H_2_O_2_ levels were observed to be greatest in malt medium (80.82 ± 2.17 %), suggesting higher activity of *G. lucidum* EPS extract. - In the present study, the radical scavenging activity of ABTS has been shown to be more active. On malt medium, *G. lucidum* EPS extracts have been reported to be the most abundant (63.75 ± 2.47 percent). | Polysaccharide, Proteins, Enzymes, Steroids, Sterols, Minerals | Mahendran et al., 2012 |
| *Ganoderma tsugae* Murrill | DPPH, RPA, ^•^OH, ferrous ion chelating assays | 1–20 mg/mL | - • Methanolic extracts from adult and newborn Ling chih showed significant chemical free radical scavenging activity (96.8% and 93.6%) at 20 mg/ ml-1, with EC50 values of 0.53 and 1.11 mg/ ml-1, respectively. In terms of reducing power, Ling chih, baby Ling chih, mycelia, and filtrate obtained EC_50_ values of 5.00, 2.28, 0.93, and 2.15 mg/ ml-1, respectively. Methanolic extracts from mature and baby Ling chih scavenged DPPH radicals by 88.4% and 93.84%, respectively, at 5 mg/ ml-1, while extracts from mycelia as well as filtrate scavenged by 85.7% and 79.3%. For iron ion chelating activity, Ling chih, baby Ling chih, mycelia, and filtrate obtained EC_50_ values of 4.82, 3.05, 1.10, and 3.41 mg/ ml^-1^, respectively. - Total phenols, which ranged from 24.0 to 35.6 mg/ml^-1^ in all methanolic extracts, have been the most prevalent found naturally antioxidants. | ascorbic acid, β-carotene, tocopherols and total phenols | Mau et al., 2005 |
| *Grifola frondosa*  (Dicks.) Gray | RPA, DPPH, ferrous ion chelating, O_2_^-^ | 1–20 mg/mL | - Based on EC_50_ values (20 mg/mL) obtained, the several extracts from *G. frondosa* examined in this study demonstrate powerful chemical free radical scavenging features in terms of reducing powers, DPPH radical, as well as superoxide anions. | total phenols, flavonoids, tocopherols, ascorbic acid and β-carotene |  |
| *Ganoderma applanatum*  (Pers.) Pat. | FRAP, DPPH | (0.5–4 mg.ml-1) | - *Ganoderma lucidium* extract has better chemical free radical scavenging activity versus DPPH, lowering power ability, and FRAP tests in this research. | total phenol, ascorbic acid, carotenoid, lycopene and ergosterol | Rajoriya et al., 2015 |
| *Geastrum saccatum*  *Fr.*  “[*Geastrum spegazzinianum* De Toni]” | O_2_^-^, ^•^OH, LPO, NO | (200–300 μg mL−1 ) | - At 0.27 mg/mL, this glucan inhibited lipid peroxidation by 59.1 %, and that may protect cells from oxidative stress by scavenging hydroxyl (77%) and superoxide (88.4%) radicals. | glucose and traces of galactose | Dore et al., 2007 |
| *Ganoderma atrum*  J.D. Zhao, L.W. Hsu & X.Q. Zhang | DPPH, 1,2,3 phentriol, SOD, CAT, GPx, MDA | 0.125–4 mg | - *In vitro* DPPH free radical scavenging tests and self-oxidation of 1,2,3-phentriol assays, the antiradical activity of the isolated polysaccharides was shown to be greater. - PSG-1's effects on immunodeficiency might be explained by the fact that it raised the activity of antioxidant enzymes (SOD, CAT, and GPx) in the spleen as well as thymus of mice given doses of 25, 50, and 100 mg/kg. | polysaccharide fractions | Chen et al., 2008; Li et al., 2017 |
| *Hericium erinaceus*  (Bull.) Pers. | DPPH, O_2_^-^ | 2 mg/mL | - The anhydrous ethanol extracts were found to have a high reducing power, high scavenging rates against DPPH and superoxide anion-free radicals (>90%), and high inhibitory rates on lipid peroxidation (>60%), as well as a significant amount of antiradical compounds. |  | Jiang et al., 2016 |
| *Hericium coralloides*  (Scop.) Pers. | DPPH, ^•^OH, ABTS, O_2_^-^, SOD, CAT, GPx, MDA |  | - *In vitro*, the petroleum ether and ethanol extracts exhibit stronger antiradical activity against DPPH, hydroxyl, ABTS^+^, and superoxide (O_2_-) radicals, respectively. - HcEAE increased the activity of superoxide dismutase (SOD), catalase (CAT), as well as glutathione peroxidase (GSH-Px) and lowered the levels of malondialdehyde (MDA) in the brains and sera of mice in a dose-dependent manner. |  | Zhang et al., 2019 |
| *Hydnum repandum*  L. | ^•^OH, H_2_O_2,_ Peroxidation of Phospholipid liposomes, linoleic acid assays | 100 µL | - All of the trufes and mushrooms investigated exhibited greater percentages of oxidation inhibition than the dietary antioxidants, according to tests based on lipid peroxidation (LOOx), deoxyribose (OHx), and peroxidase (H_2_O_2_) | protocatechuic acid and p-hydroxybenzoic acid | Murcia et al., 2002 |
| *Hygrophorus agathosmus*  (Fr.) Fr. | DPPH | 25 µL | - All of the species were able to scavenge DPPH in a concentration-dependent manner. The antiradical capacity of *B. edulis* was determined to be the highest. | Protocatechuic, p-hydroxybenzoic, p-coumaric and cinnamic acid | Ribeiro et al., 2008 |
| *Hypsizygus marmoreus*  (Peck) H.E. Bigelow | RPA, conjugated diene method, DPPH, ^•^OH, chelating of ferrous, cupric ions | 10 to 20 mg/mL | - The ethanolic, cold water, and hot water extracts exhibited moderate total antiradical activities (38.6–65.2%), while the cold water extract had a high reduction power of 0.99. The following extracts were efficient in scavenging DPPH radicals: ethanolic > hot water cold water extracts. - In terms of decreasing hydroxyl radicals, cold water extracts were more efficient than hot water extracts. The EC_50_ values for chelating activity on ferrous ions were 3.19, 0.37, and 0.40 mg/ml for ethanolic, cold water, and hot water extracts, respectively, but EC_50_ values for cupric ions were 4.42–6.63 mg/ml. | ascorbic acid, β-carotene, tocopherols and total phenols | Lee et al., 2007 |
| *Hypholoma fasciculare*  (Huds.) P. Kumm.  “[*Hypholoma acutum* (Sacc.) E. Horak,]” | ORP | 1.0mL | - *Hypholoma fasciculare* had the greatest ORP and phenolic concentration. | phenolic conten | Gevorgyan et al., 2017 |
| *Helvella crispa*  Bull. | DPPH, RPA, LPO | 5-25 µL | - This study looks at the antiradical activity of water/methanolic extracts of mushrooms in terms of phenolic components. | caffeic acid, tannic acid, gallic acid, protocatechuic acid, gentisic acid, vanillic acid, and syringic acid | Puttaraju et al., 2006 |
| *Lepista nuda*  (Bull.) Cooke | DPPH, β-carotene, TPC, TFC | 25 µL | - In this study used four complementary test systems, including DPPH free radical scavenging, β-carotene/linoleic acid systems, total phenolic compounds, and total flavonoid concentration, and discovered that *L. nuda* ethanolic extract had the highest -carotene/linoleic acid systems, total phenolic compounds, and total flavonoid concentration, as well as potential DPPH radical scavenging activity. | Total flavonoid and phenolic compounds | Mercan et al., 2006; Celal et al., 2019 |
| *Lentinus edodes*  (Berk.) Singer | DPPH, ABTS, TPC, TFC | 0.2 mL | - The radical scavenging capabilities of ABTS and DPPH were increased 2.0 and 2.2 times, respectively, as compared to the raw sample. | flavonoids and polyphenolics | Choi et al., 2006 |
| *Lactarius sanguifluus*  (Paulet) Fr. | FRAP, DPPH, β-carotene bleaching assay | 1 to 16 mg/mL | - ABTS and DPPH's radical scavenging activities were increased when compared to the control samples. | tannins, ascorbic acid, and phenolics and flavonoids | Erbiai et al., 2021 |
| *Lentinus squarrosulus*  Mont. | *In vivo:* CUPRAC, GPx, XO | 250 mg/kg (low-dose group) and 500 mg/kg (high-dose group) per day for 28 days. | - The serum CUPRAC level rose after administration with both doses, indicating that the extract was bioavailable as well as improved to overall antiradical activity. GPx activity in both serum and liver increased following treatment with 250 mg/kg of extract, and this was linked to LHP level; meanwhile, XO activity was significantly decreased after treatment with 500 mg/kg of extract. |  | Omar et al., 2015 |
| *Lactarius deliciosus*  (L.) Gray | DPPH, RPA, FRAP, NO, H_2_O_2_ | 0.0156 to 0.125 mg/mL | - At 5 mg/ml, *L. deliciosus* extract demonstrated significant antiradical activity, with DPPH, RP, H_2_O_2_, NO, and FRAP activities of 45.33 (%), 0,790 (Abs), 88.30 (%), 55.51 (%), 0.57 (%) respectively. | Content of total phenolic, flavonoid, β-carotene, and lycopene. | Bozdoğan et al., 2018 |
| *Lentinus sajor-caju* (Fr.) Fr. | ABTS, DPPH, H_2_O_2_ | 50 mg/mL | - The mushrooms solvent extracts (ethanol, methanol, and water) contain excellent antiradical properties (ABTS, DPPH, H_2_O_2_, and metal chelating activities), as well as phenol, flavonoid, and total antiradical capacity, with scavenging activity of up to 70%. |  | Singdevsachan et al., 2013 |
| *Leucopaxillus giganteus*  (Sowerby) Singer | DPPH, quandification of ascorbic acid, β-carotene, TPC, RAP, erythrocyte hemolysis mediated assay | 0.3 mL - 2.5 mL | - Using the β-carotene linoleate model system, methanolic extracts were tested for their reducing power, DPPH radicalscavenging capability, suppression of erythrocyte hemolysis, and antiradical activity. | Polyphenols, ascorbic acid, β-carotene and lycopene | Barros et al., 2007 |
| *Lactarius piperatus*  (L.) Pers. | DPPH, RPA, LPO, β-carotene, TPC, RAP, erythrocyte hemolysis mediated assay | 0.3 mL | - In reducing power, DPPH-radical scavenging capacity, suppression of erythrocyte hemolysis mediated by peroxyl radicals, and inhibition of lipid peroxidation assyas, mature spores had the greatest antioxidant contents and the lowest EC_50_ values, while immature spores had the lowest. | phenols, flavonoids, ascorbic acid,β-carotene and lycopene | Barros et al., 2007 |
| *Laetiporus sulphureus*  (Bull.) Murrill | DPPH, β-carotene, TPC, TFC | 100, 200, 400 and 800 µg/mL. | - The DPPH free radical-scavenging activity of *L. sulphureus* extract was reported to be inhibited by 14 %, 26 %, 55 %, and 86 %. - In the extract, total flavanoids were 14.2 ± 0.12 µg/mg^-1^ (quercetin equivalent), and phenolics were 63.8 ± 0.25 µg/mg^-1^ (pyrocatechol equivalent). | quercetin, α-tocopherol, pyrocatechol | Turkoglu et al., 2007; Sevindik et al., 2018 |
| *Lycoperdon molle* Pers. | DPPH, LPO, FRAP and TEAC | 100 µL | - The FRAP activity of the samples in different organic solvents, such as methanol, ethanol, acetone, and dimethyl sulfoxide (DMSO), varied from 44.00 to 89.60 %, whereas LPO activities ranged from 32.00 to 54.41% *in vitro*. - In comparison to the conventional water soluble vitamin E counterpart Trolox, the TEAC value was also shown to be greater (3.9 mM). | phosphoethanolamine, monomethyl arsenic acid, phosphatidylglycerol, phosphoionositol, phosphoserine | Singh et al., 2012 |
| *Lycoperdon perlatum*  Pers. | DPPH, FRAP | 125 μL | - Aqueous extract of *Lycoperdon perlatum* has the strongest anti-DPPH radical activity. - The less polar ethanol extract had somewhat stronger FRAP activity than the aqueous extract. |  | Novaković et al., 2015 |
| *Lactarius piperatus*  (L.) Pers. | TPC, TFC, β-carotene, lycopene, RPA, DPPH, FRAP, NO, H_2_O_2_ | 0.0156 to 0.125 mg/mL | - The antiradical activity of L. *piperatus* was showed moderate activity in DPPH, RPA, H_2_O_2_, NO and FRAP assays. |  | Bozdoğan et al., 2018 |
| *Morchella esculenta*  (L.) Pers. | RPA, DPPH, TBARS, β-carotene-linoleate assays | 30 μL | - Sample from Portugal (SP) gave higher radical scavenging activity and reducing power, while sample from Serbia (SS) showed higher lipid peroxidation inhibition | β-carotene, Lycopene, Oxalic acid, Quinic acid, Malic acid, Citric acid, Fumaric acid, Total organic acids, α-tocopherol, γ-tocopherol, δ- tocopherol, Protocatechuic acid, p-Hydroxybenzoic acid, p-Coumaric acid, Total phenolic compounds, Protocatechuic acid, p-Hydroxybenzoic acid, p-Coumaric acid | Heleno et al., 2013; Raslan et al., 2021 |
| *Morchella conica*  Pers. | DPPH, TBARS, β-carotene-linoleate assays and TPC, TFC | 160 µg/mL | - At a concentration of 160 µg/ml, inhibition values of *M. conica* ethanol extracts were determined to be 96.9% antiradical activity. | phenolics, flavonoids, tocopherol, | Turkoglu et al., 2006 |
| *Macrolepiota procera*  (Scop.) Singer | DPPH, β-carotene-linoleate and ferricyanide-prussian blue assays | 0.09 mg/mL | - Using DPPH free radical-scavenging activity radicals, suppression of -carotene bleaching radicals, and reducing power radicals, methanolic extracts demonstrated a good antiradical activity | Total phenolic, Total flavonoid, Tannin, Ascorbic acid, β-carotene, Lycopene vanillic acid , cinnamic acid, protocatechuic acid, Ferulic acid, Gallic Acid, Paraben, p-Hydroxybenzoic acid, Syringic acid, | Aytar et al., 2020; Erbiai et al., 2021 |
| *Morchella angusticeps*  Peck | ^•^OH, NO, DPPH, ABTS, LPO | 25 μL | - The extract successfully scavenged all of these radicals, preventing lipid peroxidation as well. The FRAP test was used to measure the extract's hydrogen donating ability. According to pulse radiolysis experiments using ABTS and carbonate radical, the extract significantly accelerated the decay of these radicals in a concentration-dependent manner. |  | Nitha et al., 2010 |
| *Pleurotus ostreatus*  (Jacq.) P. Kumm.  “[*Pleurotus cornucopiae* (Paulet) Quél.,]” | DPPH, TBARS, β-carotene-linoleate and ferricyanide-prussian blue assays, TPC, TFC | 1. mg/mL | - The mushroom with the greatest antiradical activity was *Pleurotus ostreatus.* | Protocatechuic acid, p-Hydroxybenzoic acid, p-Coumaric acid, Cinnamic acid, Gallic acid, | Reis et al., 2012 |
| *Pleurotus eryngii*  (DC.) Quél*.* | DPPH, TBARS, β-carotene-linoleate and ferricyanide-prussian blue assays, TPC, TFC | 20 mg/mL | - *Pleurotus eryngii* was the mushroom species with the greatest chemical free radical scavenging potential. | Protocatechuic acid, p-Hydroxybenzoic acid, p-Coumaric acid, Cinnamic acid, Gallic acid, | Reis et al., 2012 |
| *Pleurotus citrinopileatus*  Singer | CAT, SOD, GPx, RPA, ^•^OH, O_2_^-^ and ferrous ion chelating assays | 50 -200 μL | - The three species have both enzymatic and non-enzymatic antioxidant properties. *P. florida* outperformed *P. pulmonarius* and *P. citrinopileatus* in terms of reducing power, Fe^2+^ chelating activity, and total phenol content. *P. florida* showed the greatest antioxidative enzyme activity in terms of peroxidase and superoxide dismutase, whereas *P. pulmonarius* had the highest catalase activity. - The antioxidative activity of *P. florida* was higher than that of *P. pulmonarius* and *P. citrinopileatus*, emphasizing its nutraceutical and nutritional value. | Gallic acid, vanillic acid, ferulic acid, chlorogenic acid, catechin, rutin, quercetin | Yin et al., 2020 |
| *Pleurotus djamor*  (Rumph. ex Fr.) Boedijn | DPPH, DMPD, FRAP, CUPRAC, Metal chelating, LPO, phosphomolybdenum | 1-5 mg/mL | - Methanol and aqueous extracts had effective radical scavenging activities against the DPPH and DMPD radicals, as well as ferrous (Fe3+) and cupric (Cu2+) ion lowering abilities, metal chelating activities, and lipid peroxidation inhibition. Methanol and aqueous extracts had total phenolic levels of 2.79 and 5.95 mg of GAE/g, respectively. | phenolics, flavonoids, | Sudha et al., 2016 |
| *Pleurotus sajor-caju* (Fr.) Singer | DPPH, TPC | 100 µL | - The total phenolic content of mushroom powder was found to be 2.210.03 mg GAE/g of dried mushroom powder in a hot water extract, and it may inhibit 89.29 percent of DPPH radicals at a concentration of 0.1µg/ml. |  | Rashidi et al., 2016 |
| *Pleurotus cystidiosus*  O.K. Mill. | NO, DPPH, | 1.0, 2.0, 5.0, 10.0, 20.0, 30.0 and 40.0 mg/mL) | - The EC_50_ of DPPH in fractions A4-2 and A4-3 was 0.81 and 0.82 mg/mL, respectively, while the EC_50_ of NO in fractions A4-2 and A4-3 was 0.87 and 0.61 mg/mL, respectively. |  | Menikpurage et al., 2012 |
| *Pleurotus australis*  Sacc. | DPPH | 30 μL | - *P. australis* had the greatest antioxidant activity (EC_50_ of 4.03 mg/mL), whereas other polysaccharides had DPPH radical scavenging activity. |  | Ren et al., 2014 |
| *Pleurotus tuber-regium* (Fr.) Singer | ABTS, DPPH, ORAC, β-carotene bleaching assays | 0.0–20.0 µg/mL ^-1^ | - DPPH and ABTS radical scavenging capability were used to study antioxidant capabilities, with *P. ostreatus* exhibiting the best antiradical activity in the ORAC experiment. | total phenolic content, β-carotene-linoleic | Adebayo et al., 2018 |
| *Phellinus linteus*  (Berk. & M.A. Curtis) Teng | DPPH, LPO, XO assays | 300 µg/mL | - *Phellinus linteus* was as effective as vitamin C in scavenging the stable free radical DPPH at varying doses, it also inhibited LPO. |  | Song et al., 2003 |
| *Phellinus rimosus*  (Berk.) Pilát | LPO, O_2_^-^, HO, No assays |  | - The findings demonstrated that the *P. rimosus* ethyl acetate extract exhibits significant antioxidant activity *in vitro*. | Catechin, Quercetin | Ajith and Janardhanan, 2002 |
| *Phellinus merrillii*  (Murrill) Ryvarden | GPx, SOD, CAT | 0.5 g/kg, 1.0 g/kg, 2.0 g/kg | - *Phellinus merrillii* increases the activity of antioxidant enzymes such as superoxide dismutase (SOD) (86.6%), catalase (58.8%), and glutathione peroxidase (GPx) in the liver tissues (64.7%). | hispolon | Chang et al., 2007 |
| *Polyporus squamosus*  (Huds.) Fr.  “[*Polyporus septosporus* P.K. Buchanan & Ryvarden]” | DPPH, TBARS, β-carotene bleaching assays | 30 µL | - The maximal measured antiradical activity of *P. squamosus* extract (EC_50_ = 0.22 mg mL1) was established using the TBARS inhibition assay. | Oxalic acid , Quinic acid, Malic acid, Fumaric Acid, p-Hydroxybenzoic acid, p-Coumaric acid, Cinnamic acid | Mocan et al., 2018 |
| *Picoa juniperi*  Vittad. | DPPH, TBARS, β-carotene bleaching, TPC, TFC | 0.36 to 3.1 mg/mL | - The methanolic extract, which included the most phenolics and flavonoids, had the highest DPPH radical-scavenging activity and lipid peroxidation inhibitory activity. | Hexadecanoic acid, methyl ester , 9,12-Octadecadienoic acid, methyl ester, 9-Octadecenoic acid, methyl ester, Octadecanoic acid, methyl ester, Hexanal, Naphthalene, Thymol, Tetradecanoic acid , Hexadecanoic acid, 9,12-Octadecadienoic acid, Ethyl linoleate, Octadecanoic acid , vitamin A, vitamin C, vitamin E, carotenoids, anthocyanins,flavonoids and phenolic compound |  |
| *Pleurotus floridanus*  Singer | FRAP, DPPH, ^•^OH, NO, O_2_^-^, LPO, metal chelating, phosphomolybdenum and haemolytic activity assays | 200-1000 µg/mL | - At 1000 µg/ml, DPPH radical scavenging activity of 37.04 ± 0.15 and 28.04 ± 0.41 %. - In a NO test, various concentrations of *Pleurotus florida* and Calocybe indica (200-1000 µg/ml) demonstrated 21.90 ± 0.88% and 23.13 ± 1.32% inhibition at 1000 µg/ml, respectively. - FRAP 59.65 ± 0.46% - Metal chelating activity 0.77 ± 0.08% - Phosphomolybdenum 197.26 ± 1.19% |  | Prabu et al., 2016 |
| *Pleurotus pulmonarius*  (Fr.) Quél.  “[*Pleurotus ostreatus* (Jacq.) P. Kumm.,]” | DPPH, CUPRAC, metal chelating, LPO | 100 µg/mL | - All of the experiments demonstrated that the crude aqueous extract of *P. pulmonarius* had a greater impact. | Ergothioneine, ergosterol, flavonoid, and phenolic compounds are some of the components found | Abidin et al., 2016 |
| *Paecilomyces japonica* | TBARS, DPPH, LPO, α-tocopherol, SOD, GPx, CAT, MDA | 0.25 - 100 µg/mL | - In the TBARS reactant experiment in rats, water and methanol extracts resulted in large increases in rat liver cytosolic SOD, catalase, and GSH-px activity, as well as a significant decrease in MDA generation. | Ergosterol, D-Mannitol, Ascorbate, Glutathione, α-Tocopherol | Shin et al., 2001 |
| *Piptoporus betulinus*  (Bull.) P. Karst. | DPPH, TPC | 180 μL | - Polish mushroom extracts have the strongest antiradical activity against the DPPH free radical. | gentisic, gallic, protocatechuic, protocatechuic 4-OH-benzoic, vanillic, syringic, ferulic, salicylic, veratric, synapic, 4-OH-benzoic, caffeic, p-coumaric, ferulic, salicylic, veratric, synapic Rosmarinic acid with 3-OH-cinnamic acid | Nowacka et al., 2015 |
| *Russula brevipes*  Peck | TPC, TFC, β-carotene, ascorbic acid, ABTS, DPPH, ferrous ion chelating, TAC, phosphomolebdenum | 50–400 μg/mL | - The radical scavenging and metal ion chelating activities of the decoction and infusion were both higher than that of the hydro-methanol formulation. |  | Sharma et al., 2019 |
| *Russula cyanoxantha*  (Schaeff.) Fr. | RPA, DPPH, TPC, TFC | 0.05 mg/mL^-1^ | - The antioxidant activity of *Russula cyanoxantha* acetone extracts was higher than that of other mushroom extracts. Furthermore, the extracts examined demonstrated excellent decreasing power. |  | Kosanic et al., 2013 |
| *Russula delica*  Fr. | DPPH, ferrous ion chelating | 0.1–10 mg/mL | - The main component of *R. delica* ethanolic extract had a 26 % DPPH radical scavenging and chelating capacity on ferrous ions at 10 mg/ml, and a 58 % chelating ability on ferrous ions at 5 mg/ml. | Total phenolic content, Ascorbic acid, β-Carotene | Yaltirak et al., 2009 |
| *Ramaria botrytis*  (Pers.) Ricken | DPPH, ^•^OH, RPA | 0.5, 1.0, 1.5, 2.0, 2.5 and 3.0 mg/ mL^−1^ | - *Ramaria botrytis* polysaccharides (RBP) were isolated and four fractions were tested for antioxidant activity *in vitro*. On the DPPH radical, RBP-4 had a strong reducing power and high scavenging activity, but RBP-3 had a stronger capacity to scavenge hydroxyl radicals. |  | Li , 2017 |
| *Russula vinosa*  Lindblad | ABTS, ^•^OH, LPO |  | - All polysaccharides were found to have significant antioxidant capacities (EC_50_ ranged from 1.70 ± 0.42 to 65.98 ± 1.74 M trolox equivalent/g crude polysaccharide inhibition of ABTS+, EC_50_ ranged from 5.06 ± 0.12 to 127.38 ± 1.58 mg VCE/g CP OH• scavenging, and EC_50_ ranged from 0.70 ± 0.04 to 33.54 ± 0.49 mg VCE/g CP inhibition of LPO). The ABTS+ scavenging activity of *Russula vinosa* Lindblad acid extracts was the highest. |  | Li et al., 2012 |
| *Sparassis crispa*  (Wulfen) Fr. | RPA, DPPH, ferrous ion chelating assays | 50 mg/mL | - Extracts from two mycelia and filtrate were shown to have antioxidant activity, reducing power, and chemical free radical scavenging capacities, as well as antioxidant activity, reducing power, and chemical free radical scavenging capacities. | Ascorbic acid, β-Carotene, α-tocopherols, | Liang et al., 2010 |
| *Suillus bellinii*  (Inzenga) Kuntze | RPA, DPPH | 25 µL | - The capacity to scavenge DPPH was concentration-dependent in all of the species. *T. rutilans* has the highest antioxidant capacity. | Ascorbic acid, succinic acid, p-hydroxybenzoic acid, quercetin, shikimic acid, fumaric acid, oxalic acid, aconitric acid, citric acid, ketoglutaric acid, mailic + quinic acids. | Ribeiro et al., 2006 |
| *Suillus luteus*  (L.) Roussel | DPPH, ABTS, FRAP |  | - In ABTS, DPPH, and FRAP experiments, unblanched and blanched *Suillus luteus* (L.) Roussel mushrooms had the best antioxidant activity (70 – 77%). | gallic acid and (þ)-catechin | Jaworska et al., 2014 |
| *Suillus granulatus*  (L.) Roussel | CUPRAC, DPPH, ABTS, metal chelating, β-carotene-linoleic acid assays | 25 μL - 160 μL | - At 400 g/mL concentration, the ethyl acetate extract of *S. granulatus* had the highest activity in DPPH• scavenging, ABTS•+ scavenging, and CUPRAC assays, with activity of 91.52 ± 0.97%, 89.67 ± 0.15%, and 3.90 ± 0.09%, respectively. |  | Tel et al., 2014; Mushtaq et al., 2020 |
| *Sarcodon imbricatus*  (L.) P. Karst.  “[*Sarcodon squamosus* (Schaeff.) Quél.,]” | TPC, TFC, TAC, DPPH, β-carotene-linoleic acid, RPA, ferrous ion chelating assays | 0.5 mL | - Assays for free radical scavenging like DPPH, reduction power (836.0, 89.0, and 267.0 mg TEs/g extract, respectively). A metal chelating test indicated the superiority of A. impudicus (1282.0 mg TEs/g). When compared to other extracts, the relative antiradical activity index (RACI) of S. imbricatus was fairly high (0.90) | β-carotene, Toal phenolics, Total flavonoids | Tepe, 2021 |
| *Schizophyllum commune* Fr. | TPC, TFC, DPPH, ABTS,FRAP | 20-200 µg/mL | - Against DPPH and ABTS radicals, the extract from *S. commune* culture filtrate demonstrated the greatest chemical free radical scavenging activity respectively. In the FRAP experiment, the extract exhibits a high activity |  | Tangjitjaroenkun and Tangchitcharoenkhul, 2020 |
| *Tricholoma acerbum*  (Bull.) Quél. | DPPH, RPA, β-carotene bleaching, TBARS | 0.3 mL | - In the mushrooms analyzed, phytochemicals such as phenolics, tocopherols, ascorbic acid, and carotenoids are abundant. antiradical activity was discovered in all species. | phenols , flavonoids, ascorbic acid, β-carotene, lycopene | Barros et al., 2008 |
| *Tricholoma equestre*  (L.) P. Kumm*.* | TPC, TFC, β-carotene bleaching, lycopene, TEAC, RPA | 25 μL | - Total phenolics and their reducing power/scavenging effects were found in both aqueous and methanolic extracts, whereas flavonoids and their reducing power/scavenging effects were found to be modest. | polyphenols, flavonoids, β-carotene, and lycopene | Robaszkiewicz et al., 2010 |
| *Tricholoma giganteum*  Massee | DPPH | 1 mL | - The DPPH scavenging activity of methanol extracts of *Tricholoma giganteum* basidiocarps and mycelial mat was about 60%. *T. giganteum* fruiting body and mycelium have EC_50_ values of 0.7 mg/ml and 2.0 mg/ml, respectively. |  | Pushpa et al., 2014 |
| *Tricholomopsis rutilans*  (Schaeff.) Singer | DPPH |  | - *T. rutilans* has the highest antioxidant capacity in a concentration-dependent manner. | oxalic, quinic, citric, malic, and fumaric acids | Ribeiro et al., 2006 |
| *Termitomyces microcarpus*  (Berk. & Broome) R. Heim | DPPH | 0 - 10 mg/mL | - *T. microcarpus* and its related AEAC were found to have strong radical scavenging properties of 74.92 % and 0.29 mg/g, respectively, which is predicted given the high quantity of antiradical components such as phenolics (1.02 ± 0.16 mg/g) and carotenoids. | Phenolics, carotenoids | Rajoriya and Gupta, 2015 |
| *Termitomyces schimperi*  (Pat.) R. Heim | RPA, DPPH | 0 - 10 mg/mL | - The scavenging activities of TS and TSK extracts in aqueous and ethanolic form were concentration dependent. The ethanolic extract of TS has a greater chemical free radical scavenging activity than the aqueous extract (p < 0.001), with EC_50_ values of 1.28 ± 0.03 and 1.97 ± 0.23 mg/ml, respectively. |  | Owusu, 2017 |
| *Termitomyces mummiformis* and *Termitomyces heimii*  Natarajan | RPA, DPPH, LPO | 5-25 µL | - The water extract RPA inhibition effect, whereas the methanolic extract had 11.2 mg phenolics/g, 275 units of RPA/g. - *Termitomyces mummiformis* had an AI of 86%, following *T. heimii* in the "very high" category. Potent inhibitions of lipid peroxidation of 100 and 69 % were discovered in *T. heimii* and *T. mummiformis*, respectively. - Water extracts were from 34 to 49 % of the dry weight of the mushroom fruiting body, whereas methanolic extracts were 20 to 32 % of the dry weight. | tannic acid, gallic acid, protocatacheuic acid, and gentisic acid. | Puttaraju et al., 2006 |
| *Termitomyces albuminosus*  (Berk.) R. Heim | RPA, DPPH, ^•^OH, ferrous ion chelation | 1. µL | - Methanolic extracts from the three mycelia demonstrated substantial antiradical activity (85.4–94.7 %) and reducing powers of 0.97–1.02 at a concentration of 25 mg/ml. The scavenging actions on DPPH radicals were 78.8–94.1 % at 10 mg/ ml^-1^. - Chelating effects on ferrous ions were strong (90.3–94.4%) at 10 mg/ ml^-1^. | ascorbic acid, β-carotene, tocopherols and total phenols | Mau et al., 2004 |
| *Termitomyces robustus*  (Beeli) R. Heim | DPPH, ferrous ion chelation, ^•^OH, O_2_^-^ | 2 mg/mL | - It has high scavenging activity against 2, DPPH, and ferrous ion radicals at a dose of 2 mg/mL. TRE and TCE, on the other hand, showed a higher superoxide anion scavenging activity at 2 mg/mL. - All extracts (TCE, TRE, LSE, and LZE) had a scavenging effect on hydroxyl radicals that was equivalent to butylated Hydroxytoluene (BHT). |  | Oyetayo, 2009 |
| *Terfezia claveryi*  Chatin | ABTS, FRAP, DPPH | 2.5, 25, 50, 100, 200 µg/mL. | - Strong chemical free radical scavenging activity found in the ethanol extract in DPPH, FRAP, and ABTS assays respectively. | Hexadecanoic acid, Octadecadienoic acid, Oleic acid, Ascorbic acid, Squalene, Campestanol, Stigmasterol, beta-Sitosterol, Lupeol | Dahham et al., 2018 |
| *Tremella fuciformis*  Berk. | DPPH, ABTS, LDL oxidation | 25, 50, 100, 200 μg/mL | - Total phenolic content (66.31 g CAE/mg extract) and flavonoids content (5.12 g QE/mg extract) were greatest in the chloroform subfraction, suggesting the maximum antiradical activity. - With 7.89 mol trolox/mg extract, the chloroform subfraction showed the most ABTS+ radical scavenging activity of all the subfractions. The DPPH radical scavenging and LDL oxidation inhibitory effectiveness of this subfraction were likewise the best. | Total phenolic, Total flavonoid, 4-hydroxybenzoic acid, gentisic acid, 4-coumaric acid | Li et al., 2014 |
| *Trametes versicolor*  (L.) Lloyd | DPPH, ABTS, metal chelating activity | 0.0 - 3.0 mg/mL | - 80 U/mL -1,3-glucanase increased the antiradical activity of enzymatic hydrolysates produced from *T. versicolor* polysaccharopeptides (PSPs-EH80). - Polysaccharides showed metal chelating, ABTS, and DPPH radical scavenging activities respectively. |  | Jhan et al., 2016 |
| *Trametes orientalis*  (Yasuda) Imazeki | GPx, SOD, CAT, MDA | 50, 100 and 200 mg kg^−1^ | - *Trametes orientalis* inhibits SOD, CAT, and GSH-Px activity in mouse lung tissue (p < 0.05 or p < 0.01), suggesting that PM2.5 exposure might compromise the enzymatic antioxidant system. |  | Zheng et al., 2019 |
| *Verpa conica*  (O.F. Müll.) Sw. | DPPH, O_2_^-^ | 100 - 400 µg/mL | - The methanolic extract of *Verpa conica* was shown to be more efficient in scavenging O_2_ and DPPH radicals than other mushroom species. | total phenolic, α-tocopherol, β-carotene | Elmastas et al., 2007 |
| *Volvariella volvacea*  (Bull.) Singer | GSH, TPC, TFC, DPPH | 50 - 100 μL | - The greatest total phenolic content was found in rice bran broth, with 23.19 mg AAE/g sample for *V. volvacea* mycelia, while the highest total phenolic content was found in coconut water, with 25.52 mg AAE/g sample for *S. commune* mycelia. - With 21.19 and 19.45 %, respectively, the radical scavenging activity of Mycelia of *V. volvacea* grown in coconut water and S. commune grown in rice bran broth was highest. | total phenol, flavonoids, lycopene, total carotenoids, vitamin C, vitamin A | Sudha et al., 2008; Dulay et al., 2016 |
| *Wolfiporia cocos*  (F.A. Wolf) Ryvarden & Gilb. | DPPH |  | - The antiradical activity *in vitro* was preserved up to 80% in the DPPH experiment. | Methy anthranilate, linalool and 2-phenylethanol | Rigling et al., 2021 |
| *Wolfiporia extensa*  (Peck) Ginns | FRAP, ABTS, DPPH | 10 - 100 µL | - In DPPH and ABTS tests for antiradical activity, *Wofiporia extensa* had strong chemical free radical scavenging activity. In the FRAP experiment, *Wofiporia extensa* showed strong ferric reducing capacity. | 9,12-, 2-Furaldehyde, Octadecadienoic acid, 9-Octadecenoic acid, hexadecanoic acid, 5-ydroxymethylfurfural, 13-Docosenamide | Choi et al., 2016 |

ABTS: 2,2′-azino-bis[3-ethylbenzothiazoline-6-sulphonic acid]; CAT: Catalase; CUPRAC: Cupric-reducing antioxidant capacity; DMPD: N,N-dimethyl-p-phenylenediamine; DPPH: 2,2-diphenyl-1-picrylhydrazyl; FRAP: Ferric Antioxidant Reducing Power; FRAP: Ferric reducing antioxidant power assay; GSH-Px: Glutathione peroxidase; H_2_O_2_: Hydrogen peroxide; ^•^OH: Hydroxyl radical; LPO: Lipid peroxidation; MDA: Malondialdehyde; NO: Nitric oxide; O_2_-: Superoxide radical; ORAC: Oxygen radical absorbance capacity; ORP: Oxidation reduction potential; OSI: Oxidative stress index; RPA: Reduced power assay; SOD: Superoxide dismutase; TAC: Total antioxidant capacity; TAS: Total antioxidant status; TBARS: Thiobarbituric acid reactive substances; TEAC: Trolox equivalent antioxidative activity; TFC: Total flavonoid contents; TOS: Total oxidant status; TPC: Total phenolic contents; XO: Xanthine oxidase.

**References**

Abidin, M. H. Z., Abdullah, N., and Abidin, N. Z. (2016). Protective effect of antioxidant extracts from grey oyster mushroom, Pleurotus pulmonarius (Agaricomycetes), against human low-density lipoprotein oxidation and aortic endothelial cell damage. *Int. J. Med. Mushrooms*. 18(2):109-21.

Adebayo, E. A., Martínez-Carrera, D., Morales, P., Sobal, M., Escudero, H., Meneses, M. E., et al. (2018). Comparative study of antioxidant and antibacterial properties of the edible mushrooms Pleurotus levis, P. ostreatus, P. pulmonarius and P. tuber-regium. *Int. J. Food Sci. Technol.* 53, 1316–1330.

Ajith, T. A., and Janardhanan, K. K. (2002). Antioxidant and antihepatotoxic activities of Phellinus rimosus (Berk) Pilat. *J. Ethnopharmacol*. 81, 387–391.

Aytar, E. C., Akata, İ., and Leyla, A. (2020). Antioxidant and Antimicrobial Activities of Armillaria Mellea and Macrolepiota Procera Extracts. *Mantar Derg*. 11, 121–128.

Barros, L., Baptista, P., and Ferreira, I. C. (2007a). Effect of Lactarius piperatus fruiting body maturity stage on antioxidant activity measured by several biochemical assays. *Food Chem. Toxicol.* 45, 1731–1737.

Barros, L., Venturini, B. A., Baptista, P., Estevinho, L. M., and Ferreira, I. C. (2008). Chemical composition and biological properties of Portuguese wild mushrooms: a comprehensive study. *J. Agric. Food Chem.* 56, 3856–3862.

Bozdoğan, A., Ulukanlı, Z., Bozok, F., Eker, T., Doğan, H. H., and Büyükalaca, S. (2018). Antioxidant potential of Lactarius deliciosus and Pleurotus ostreatus from Amanos Mountains. *Adv. Life Sci.* 5(3), 113-120.

Celal, B. A. L., Sevindik, M., Akgul, H., and Selamoglu, Z. (2019). Oxidative stress index and antioxidant capacity of Lepista nuda collected from Gaziantep/Turkey. *Sigma J. Eng. Nat. Sci.* 37(1), 1-5.

Chang, H.-Y., Peng, W.-H., Sheu, M.-J., Huang, G.-J., Tseng, M.-C., Lai, M.-T., et al. (2007). Hepatoprotective and Antioxidant Effects of Ethanol Extract from Phellinus merrillii on Carbon Tetrachloride-Induced Liver Damage. *Am. J. Chin. Med*. 35, 793–804. doi:10.1142/S0192415X07005272.

Chen, Y., Xie, M.-Y., Nie, S.-P., Li, C., and Wang, Y.-X. (2008). Purification, composition analysis and antioxidant activity of a polysaccharide from the fruiting bodies of Ganoderma atrum. *Food Chem*. 107, 231–241.

Choi, S.-H., Lee, S.-J., Jo, W.-S., Choi, J.-W., and Park, S.-C. (2016b). Comparison of ingredients and antioxidant activity of the domestic regional Wolfiporia extensa. *Korean J. Mycol*. 44, 23–30.

Choi, Y., Lee, S. M., Chun, J., Lee, H. B., and Lee, J. (2006). Influence of heat treatment on the antioxidant activities and polyphenolic compounds of Shiitake (Lentinus edodes) mushroom. *Food Chem*. 99, 381–387.

Cui, Y., Kim, D.-S., and Park, K.-C. (2005). Antioxidant effect of Inonotus obliquus. *J. Ethnopharmacol.* 96, 79–85.

Dahham, S. S., Al-Rawi, S. S., Ibrahim, A. H., Majid, A. S. A., and Majid, A. M. S. A. (2018). Antioxidant, anticancer, apoptosis properties and chemical composition of black truffle Terfezia claveryi. *Saudi J. Biol. Sci.* 25, 1524–1534.

Dore, C. M. G., Azevedo, T. C., de Souza, M. C., Rego, L. A., de Dantas, J. C., Silva, F. R., et al. (2007). Antiinflammatory, antioxidant and cytotoxic actions of β-glucan-rich extract from Geastrum saccatum mushroom. *Int. Immunopharmacol.* 7, 1160–1169.

Dulay, R. M. R., Vicente, J. J. A., Cruz, A. D., Gagarin, J. M., Fernando, W., Kalaw, S. P., et al. (2016). Antioxidant activity and total phenolic content of Volvariella volvacea and Schizophyllum commune mycelia cultured in indigenous liquid media. *Mycosphere* 7, 131–138.

Elmastas, M., Isildak, O., Turkekul, I., and Temur, N. (2007). Determination of antioxidant activity and antioxidant compounds in wild edible mushrooms. *J. Food Compos. Anal*. 20, 337–345.

Erbiai, E. H., Bouchra, B., da Silva, L. P., Lamrani, Z., Pinto, E., da Silva, J. C., et al. (2021). Chemical composition and antioxidant and antimicrobial activities of Lactarius sanguifluus, a wild edible mushroom from northern Morocco. *Euro-Mediterr. J. Environ. Integr*. 6, 1–12.

Gąsecka, M., Magdziak, Z., Siwulski, M., and Mleczek, M. (2018a). Profile of phenolic and organic acids, antioxidant properties and ergosterol content in cultivated and wild growing species of Agaricus. *Eur. Food Res. Technol.* 244, 259–268.

Gąsecka, M., Siwulski, M., and Mleczek, M. (2018b). Evaluation of bioactive compounds content and antioxidant properties of soil-growing and wood-growing edible mushrooms. *J. Food Process. Preserv.* 42, e13386.

Gevorgyan, V. S., Nanagulyan, S. G., Chantikyan, А. А., and Seferyan, T. Y. (2017). Assessment of antioxidant activities of some medicinal fungal extracts. *ԵՊՀ Գիտական Տեղեկագիր-Քիմիա ԵՒ Կենսաբանություն* 51, 163–165.

Ghosh, S., Sett, S., Saha, R., Roy, A., and Acharya, K. (2021). Comparative phytochemical screening and antioxidant properties of infusion, decoction and hydroalcoholic extracts of wood ear mushrooms; Auricularia delicata and Auricularia mesenterica. *Indian Phytopathol*. 74, 113–121.

Heleno, S. A., Stojković, D., Barros, L., Glamočlija, J., Soković, M., Martins, A., et al. (2013). A comparative study of chemical composition, antioxidant and antimicrobial properties of Morchella esculenta (L.) Pers. from Portugal and Serbia. *Food Res. Int*. 51, 236–243.

Hsiao, G., Shen, M.-Y., Lin, K.-H., Lan, M.-H., Wu, L.-Y., Chou, D.-S., et al. (2003). Antioxidative and hepatoprotective effects of Antrodia camphorata extract. *J. Agric. Food Chem.* 51, 3302–3308.

Jaszek, M., Osińska-Jaroszuk, M., Janusz, G., Matuszewska, A., Stefaniuk, D., Sulej, J., et al. (2013). New bioactive fungal molecules with high antioxidant and antimicrobial capacity isolated from Cerrena unicolor idiophasic cultures. *BioMed Res. Int.* 2013.

Jaworska, G., Pogoń, K., Bernaś, E., Skrzypczak, A., and Kapusta, I. (2014). Vitamins, phenolics and antioxidant activity of culinary prepared Suillus luteus (L.) Roussel mushroom. *LWT-Food Sci. Technol.* 59, 701–706.

Jaworska, G., Pogoń, K., Skrzypczak, A., and Bernaś, E. (2015). Composition and antioxidant properties of wild mushrooms Boletus edulis and Xerocomus badius prepared for consumption. *J. Food Sci. Technol.* 52, 7944–7953.

Jhan, M.-H., Yeh, C.-H., Tsai, C.-C., Kao, C.-T., Chang, C.-K., and Hsieh, C.-W. (2016). Enhancing the antioxidant ability of Trametes versicolor polysaccharopeptides by an enzymatic hydrolysis process. *Molecules* 21, 1215.

Jiang, S., Wang, Y., and Zhang, X. (2016). Comparative studies on extracts from Hericium erinaceus by different polarity reagents to gain higher antioxidant activities. *Exp. Ther. Med.* 12, 513–517.

Kosanic, M., Rankovic, B., and Dasic, M. (2013). Antioxidant and antimicrobial properties of mushrooms. *Bulg. J. Agric. Sci*. 19, 1040–1046.

Kozarski, M., Klaus, A., Niksic, M., Jakovljevic, D., Helsper, J. P., and Van Griensven, L. J. (2011). Antioxidative and immunomodulating activities of polysaccharide extracts of the medicinal mushrooms Agaricus bisporus, Agaricus brasiliensis, Ganoderma lucidum and Phellinus linteus. *Food Chem.* 129, 1667–1675.

Kozarski, M., Klaus, A., Nikšić, M., Vrvić, M.M., Todorović, N., Jakovljević, D. and Van Griensven, L.J., (2012). Antioxidative activities and chemical characterization of polysaccharide extracts from the widely used mushrooms Ganoderma applanatum, Ganoderma lucidum, Lentinus edodes and Trametes versicolor. *J. Food Compos. Anal*., 26(1-2), pp.144-153.

Lee, Y.-L., Yen, M.-T., and Mau, J.-L. (2007). Antioxidant properties of various extracts from Hypsizigus marmoreus. *Food Chem*. 104, 1–9.

Li, B., Lu, F., Suo, X., Nan, H., and Li, B. (2010). Antioxidant properties of cap and stipe from Coprinus comatus. *Molecules* 15, 1473–1486.

Li, H., Lee, H.-S., Kim, S.-H., Moon, B., and Lee, C. (2014a). Antioxidant and anti-inflammatory activities of methanol extracts of Tremella fuciformis and its major phenolic acids. *J. Food Sci.* 79, C460–C468.

Li, W.-J., Li, L., Zhen, W.-Y., Wang, L.-F., Pan, M., Lv, J.-Q., et al. (2017). Ganoderma atrum polysaccharide ameliorates ROS generation and apoptosis in spleen and thymus of immunosuppressed mice. *Food Chem. Toxicol*. 99, 199–208.

Li, X., Wang, Z., Wang, L., Walid, E., and Zhang, H. (2012). In vitro antioxidant and anti-proliferation activities of polysaccharides from various extracts of different mushrooms. Int. *J. Mol. Sci.* 13, 5801–5817.

Liang, C.-H., Tsai, S.-Y., Huang, S.-J., Liang, Z.-C., and Mau, J.-L. (2010). Taste quality and antioxidant properties of medicinal mushrooms Phellinus linteus and Sparassis crispa mycelia. *Int. J. Med. Mushrooms*,12(2), 141–150

Lin, W.-Y., Yang, M. J., Hung, L.-T., and Lin, L.-C. (2013). Antioxidant properties of methanol extract of a new commercial gelatinous mushrooms (white variety of Auricularia fuscosuccinea) of Taiwan. *Afr. J. Biotechnol.* 12, 6210–6221.

Liu, J., Jia, L., Kan, J., and Jin, C. (2013). In vitro and in vivo antioxidant activity of ethanolic extract of white button mushroom (Agaricus bisporus). *Food Chem. Toxicol*. 51, 310–316.

Lung, M.-Y., and Chang, Y.-C. (2011). Antioxidant properties of the edible basidiomycete Armillaria mellea in submerged cultures. *Int. J. Mol. Sci*. 12, 6367–6384.

Luo, Jingwen., Fengling, S.I., Zixuan, G.U., Wang, Linling., & Meng, Xiuxiu. (2018). Antioxidant and Antimicrobial Activities of Polysaccharides from Three Species of Auricularia Available at: https://www.spkx.net.cn/EN/10.7506/spkx1002-6630-201819011 [Accessed February 24, 2022].

Mahendran, S., Anandapandian, K. T. K., Shankar, T., Chellaram, C., and Vijayabaskar, P. (2012). Antioxidant properties of Ganoderma lucidum crude exopolysaccharide. *Indian J* *Innov Dev* 1, 1–6.

Mau, J. L., Chao, G. R., & Wu, K. T. (2001). Antioxidant properties of methanolic extracts from several ear mushrooms. J. Agr. Food Chem. 49(11), 5461-5467.

Mau, J.-L., Chang, C.-N., Huang, S.-J., and Chen, C.-C. (2004). Antioxidant properties of methanolic extracts from Grifola frondosa, Morchella esculenta and Termitomyces albuminosus mycelia. *Food Chem*. 87, 111–118.

Mau, J.-L., Tsai, S.-Y., Tseng, Y.-H., and Huang, S.-J. (2005). Antioxidant properties of methanolic extracts from Ganoderma tsugae. *Food Chem*. 93, 641–649.

Menikpurage, I. P., Soysa, S., and Abeytunga, D. T. U. (2012). Antioxidant activity and cytotoxicity of the edible mushroom, Pleurotus cystidiosus against Hep-2 carcinoma cells. *J. Natl. Sci. Found. Sri Lanka* 40, 107–114.

Mercan, N., Duru, M. E., Turkoglu, A., Gezer, K., Kivrak, I., and Turkoglu, H. (2006). Antioxidant and antimicrobial properties of ethanolic extract fromLepista nuda (Bull.) Cooke. *Ann. Microbiol*. 56, 339–344.

Mocan, A., Fernandes, Â., Barros, L., Crişan, G., Smiljković, M., Soković, M., et al. (2018). Chemical composition and bioactive properties of the wild mushroom Polyporus squamosus (Huds.) Fr: A study with samples from Romania. *Food Funct*. 9, 160–170.

Murcia, M. A., Martinez-Tome, M., Jiménez, A. M., Vera, A. M., Honrubia, M., and Parras, P. (2002). Antioxidant activity of edible fungi (truffles and mushrooms): losses during industrial processing. *J. Food Prot*. 65, 1614–1622.

Mushtaq, W., Hayri, B. A. B. A., Akata, I., and Sevindik, M. (2020). Antioxidant potential and element contents of wild edible mushroom Suillus granulatus. *Kahramanmaraş Sütçü İmam Üniversitesi Tarım ve Doğa Dergisi,* 23(3), 592-595.

Nitha, B., De, S., Adhikari, S. K., Devasagayam, T. P. A., and Janardhanan, K. K. (2010). Evaluation of free radical scavenging activity of morel mushroom, Morchella esculenta mycelia: a potential source of therapeutically useful antioxidants. *Pharm. Biol.* 48, 453–460.

Novaković, A. R., Karaman, M. A., Matavulj, M. N., Pejin, B. M., Belović, M. M., Radusin, T. I., et al. (2015). An insight into in vitro bioactivity of wild-growing puffball species Lycoperdon perlatum (Pers) 1796. *Food Feed Res*. 42, 51–58.

Nowacka, N., Nowak, R., Drozd, M., Olech, M., Los, R., and Malm, A. (2015). Antibacterial, antiradical potential and phenolic compounds of thirty-one polish mushrooms. *PloS One* 10, e0140355.

O Oyetayo, V., Dong, C.-H., and Yao, Y.-J. (2009b). Antioxidant and antimicrobial properties of aqueous extract from Dictyophora indusiata. *Open Mycol*. *J.* 3.

Omar, N. A. M., Abdullah, S., Abdullah, N., Kuppusamy, U. R., Abdulla, M. A., and Sabaratnam, V. (2015). Lentinus squarrosulus (Mont.) mycelium enhanced antioxidant status in rat model. *Drug Des. Devel. Ther*. 9, 5957.

Owusu, B. E. (2017). Antioxidant And Selective Cytotoxic Activities On Cancer Cell Lines By Bioactivatable Compounds In Extracts Of Termitomyces Schimperi (Lyophyllaceae) (Doctoral dissertation, University of Ghana). http://ugspace.ug.edu.gh/handle/123456789/23028

Oyetayo, V. O. (2009). Free radical scavenging and antimicrobial properties of extracts of wild mushrooms. *Braz. J. Microbiol*. 40, 380–386.

Packialakshmi, B., & Sudha, G. (2016). Charumathy. Bioactive constituents and antioxidant efficacy of Auricularia polytricha. Asian J. Pharm. Clin. Res, 91, 125-129.

Petrović, J., Glamočlija, J., Stojković, D., Ćirić, A., Barros, L., Ferreira, I. C., & Soković, M. (2015). Nutritional value, chemical composition, antioxidant activity and enrichment of cream cheese with chestnut mushroom Agrocybe aegerita (Brig.) Sing. J. Food Sci. Technol. 52(10), 6711-6718.

Prabu, M., and Kumuthakalavallia, R. (2016). Antioxidant activity of oyster mushroom (Pleurotus florida [Mont.] singer) and milky mushroom (Calocybe indica P and C). *Int J Curr Pharm. Res* 8, 1–4.

Pushpa, H., Anand, M., Kasimaiah, P., Pradeep, P., and Purushothama, K. B. (2014). Antioxidant and anticancer activity of Tricholoma giganteum Massee an edible wild mushroom. *Acad J Can Res* 7, 146–151.

Puttaraju, N. G., Venkateshaiah, S. U., Dharmesh, S. M., Urs, S. M. N., and Somasundaram, R. (2006). Antioxidant activity of indigenous edible mushrooms. *J. Agric. Food Chem*. 54, 9764–9772.

Rajoriya, A., and Gupta, N. (2015). Proximate and Antioxidant Activity of Mycelia of Termitomyces microcarpus and Amanita loosii. *Agric. Res. Technol*. Open Access J. 1, 13–16.

Rajoriya, A., Tripathy, S. S., and Gupta, N. (2015). In vitro antioxidant activity of selected Ganoderma species found in Odisha, India. *Trop. Plant Res*. 2, 72–77.

Rashidi, A. M., and Yang, T. A. (2016). Nutritional and antioxidant values of oyster mushroom (P. sajor-caju) cultivated on rubber sawdust. *Int. J. Adv. Sci. Eng. Inf. Technol*. 6, 161–164.

Raslan, E. C., Altuntas, D., Hayri, B. A. B. A., Celal, B. A. L., Akgül, H., Akata, I., & Sevindik, M. (2021). Some biological activities and element contents of ethanol extract of wild edible mushroom morchella esculenta. *Sigma J. Eng. Nat. Sci*. 39(1), 24-28.

Reis, F. S., Stojković, D., Soković, M., Glamočlija, J., Ćirić, A., Barros, L., et al. (2012). Chemical characterization of Agaricus bohusii, antioxidant potential and antifungal preserving properties when incorporated in cream cheese. *Food Res. Int*. 48, 620–626.

Ren, L., Hemar, Y., Perera, C. O., Lewis, G., Krissansen, G. W., & Buchanan, P. K. (2014). Antibacterial and antioxidant activities of aqueous extracts of eight edible mushrooms. *Bioact. Carbohydr. Diet. Fibre*, 3(2), 41-51.

Ribeiro, B., Lopes, R., Andrade, P. B., Seabra, R. M., Gonçalves, R. F., Baptista, P., et al. (2008). Comparative study of phytochemicals and antioxidant potential of wild edible mushroom caps and stipes. *Food Chem*. 110, 47–56.

Ribeiro, B., Rangel, J., Valentao, P., Baptista, P., Seabra, R. M., and Andrade, P. B. (2006). Contents of carboxylic acids and two phenolics and antioxidant activity of dried Portuguese wild edible mushrooms. *J. Agric. Food Chem*. 54, 8530–8537.

Rigling, M., Liu, Z., Hofele, M., Prozmann, J., Zhang, C., Ni, L., et al. (2021). Aroma and catechin profile and in vitro antioxidant activity of green tea infusion as affected by submerged fermentation with Wolfiporia cocos (Fu Ling). *Food Chem*. 361, 130065.

Robaszkiewicz, A., Bartosz, G., \Lawrynowicz, M., and Soszyński, M. (2010). The Role of Polyphenols, 𝜷-Carotene, and Lycopene in the Antioxidative Action of the Extracts of Dried, Edible Mushrooms. *J. Nutr. Metab*. 2010, 173274. doi: 10.1155/2010/173274.

Sevindik, M. (2019). Wild edible mushroom Cantharellus cibarius as a natural antioxidant food. *Turk. J. Agric.-Food Sci. Technol*. 7, 1377–1381.

Sevindik, M., Akgul, H., Bal, C., & Selamoglu, Z. (2018). Phenolic contents, oxidant/antioxidant potential and heavy metal levels in Cyclocybe cylindracea. *Indian J. Pharm. Educat. Res*. 52(3), 437-441.

Sevindik, M., Akgul, H., Dogan, M., Akata, I., & Selamoglu, Z. (2018). Determination of antioxidant, antimicrobial, DNA protective activity and heavy metals content of Laetiporus sulphureus. *Fresenius Environ. Bull*. 27(3), 1946-1952.

Sharma, Y. P., Sharma, R., Khatua, S., and Acharya, K. (2019). Morphotaxonomy and comparative mycochemical study and antioxidant activity of hydromethanol, infusion and decoction extracts from Russula brevipes Peck. *Indian Phytopathol*. 72, 445–452.

Shin, K. H., Lim, S. S., Lee, S. H., Lee, Y. S., and Cho, S. Y. (2001). Antioxidant and immunostimulating activities of the fruiting bodies of Paecilomyces japonica, a new type of Cordyceps sp. *Ann. N. Y. Acad. Sci.* 928, 261–273.

Singdevsachan, S. K., Patra, J. K., and Thatoi, H. (2013). Nutritional and bioactive potential of two wild edible mushrooms (Lentinus sajor-caju and Lentinus torulosus) from Similipal Biosphere Reserve, *India. Food Sci. Biotechnol*. 22, 137–145.

Singh, P., Singh, A., D’Souza, L., Roy, U., and Singh, S. (2012). Chemical constituents and antioxidant activity of the Arctic mushroom Lycoperdon molle Pers. *Polar Res.* 31, 17329.

Slawinska, A., Radzki, W., & Kalbarczyk, J. (2013). Antioxidant activities and polyphenolics content of Flammulina velutipes mushroom extracts. Herba Polonica, 59(3).

Song, Y. S., Kim, S.-H., Sa, J.-H., Jin, C., Lim, C.-J., and Park, E.-H. (2003). Anti-angiogenic, antioxidant and xanthine oxidase inhibition activities of the mushroom Phellinus linteus. *J. Ethnopharmacol*. 88, 113–116.

Sudha, A., Lakshmanan, P., and Kalaiselvan, B. (2008). Antioxidant properties of paddy straw mushroom (Volvariella volvacea (Bull. ex Fr.)) Sing. *Int. J. Appl. Agri. Res.* 3, 9–16.

Sudha, G., Janardhanan, A., Moorthy, A., Chinnasamy, M., Gunasekaran, S., Thimmaraju, A., et al. (2016). Comparative study on the antioxidant activity of methanolic and aqueous extracts from the fruiting bodies of an edible mushroom Pleurotus djamor. *Food Sci. Biotechnol.* 25, 371–377.

Tangjitjaroenkun, J., and Tangchitcharoenkhul, R. (2020). Antioxidant properties of the extract from culture filtrate of Schizophyllum commune. *Res. J. Pharm. Technol*. 13, 3365–3371.

Tekeli, Y., Dogan, H. H., and Uslu, U. (2008). Determination of antioxidant activity of Cantharellus cibarius Fr. *Asian J. Chem*. 20, 2381.

Tel, G., Deveci, E., Küçükaydın, S., Özler, M. A., Duru, M. E., & Harmandar, M. (2014). Evaluation of antioxidant activity of Armillaria tabescens, Leucopaxillus gentianeus and Suillus granulatus: The mushroom species from Anatolia. *Eurasian J. Anal. Chem.* 8(3), 136-147.

Tepe, A. Ş. (2021). Chemical compositions and antioxidant activities of four different mushroom species collected from Turkey. *Int. J. Second. Metab*. 8, 214–226.

Tsai, S.-Y., Huang, S.-J., and Mau, J.-L. (2006). Antioxidant properties of hot water extracts from Agrocybe cylindracea. *Food Chem.* 98, 670–677.

Turkoglu, A., Duru, M. E., Mercan, N., Kivrak, I., and Gezer, K. (2007). Antioxidant and antimicrobial activities of Laetiporus sulphureus (Bull.) Murrill. *Food Chem*. 101, 267–273.

Turkoglu, A., Kivrak, I., Mercan, N., Duru, M. E., Gezer, K., and Turkoglu, H. (2006). Antioxidant and antimicrobial activities of Morchella conica Pers. *Afr. J. Biotechnol*. 5 (11), 1146-1150,

Yaltirak, T., Aslim, B., Ozturk, S., and Alli, H. (2009). Antimicrobial and antioxidant activities of Russula delica Fr. *Food Chem. Toxicol*. 47, 2052–2056.

Yamaguchi, Y., Kagota, S., Nakamura, K., Shinozuka, K., and Kunitomo, M. (2000). Antioxidant activity of the extracts from fruiting bodies of cultured Cordyceps sinensis. Phytother. Res. *Int. J. Devoted Pharmacol. Toxicol. Eval. Nat. Prod. Deriv.* 14, 647–649.

Yin, C., Noratto, G.D., Fan, X., Chen, Z., Yao, F., Shi, D. and Gao, H., 2020. The impact of mushroom polysaccharides on gut microbiota and its beneficial effects to host: A review. *Carbohydr. Polym*. 250, p.116942.

Zhang, J., Zhang, J., Zhao, L., Shui, X., Wang, L., and Wu, Y. (2019). Antioxidant and anti-aging activities of ethyl acetate extract of the coral tooth mushroom, Hericium coralloides (Agaricomycetes). *Int. J. Med. Mushrooms*, 21(6), 561-570. doi: 10.1615/IntJMedMushrooms.2019030840.

Zheng, Y., Bai, L., Zhou, Y., Tong, R., Zeng, M., Li, X., et al. (2019). Polysaccharides from Chinese herbal medicine for anti-diabetes recent advances. *Int. J. Biol. Macromol*. 121, 1240–1253.
