# Supplementary material for: The Antioxidant Properties of Mushroom Polysaccharides can Potentially Mitigate Oxidative Stress, Beta-Cell Dysfunction and Insulin Resistance
Source: Front Pharmacol. 2022 May 5;13:874474. doi: 10.3389/fphar.2022.874474 (PMC9117613; doi:10.3389/fphar.2022.874474)
Supplement: Supplementary file 3 [file Table2.docx]

**Table 2:** Mushroom polysaccharides effects on hyperglycemia and other diabetic complications (*in vitro* and *in vivo* experimental study reports).

| **Scientific name** | **Polysaccharides name** | **Experiments models** | **Doses** | **Key results** | **References** |
| --- | --- | --- | --- | --- | --- |
| *Agaricus bisporus*  (J.E. Lange) Imbach and *Pleurotus ostreatus*  (Jacq.) P. Kumm*.*  “[*Pleurotus cornucopiae* (Paulet) Quél.,]” | Polysaccharide | Wistar rats were given alloxan. | 15 every day for four weeks | - The levels of ALP, AST, ALT, hyperlipidemia, HDL-C, and total protein were all lowered, while HDL-C and total protein levels were all elevated. | Nweze et al., 2020 |
| *Agaricus bisporus*  (J.E. Lange) | Polysaccharide | Wistar rats were given alloxan | 30 days of oral treatment at a dosage of 200 mg/kg | - Lipid metabolism has improved, as have antioxidant levels. | Ekowati et al., 2018 |
|  | Polysaccharide | Wistar rats were given alloxan | Oral therapy for 2 weeks at a dosage of 500 mg/kg | - MDA and BGL concentrations have been reduced. SOD activity has risen. | Balakrishnan et al., 2018 |
|  | Polysaccharide | C57BL/6 mice | Oral therapy with 75–100 g dosages for 2–7 weeks | - Bacteria from Dorea, Clostridium, and Peptostreptococcaceae were reduced, while Helicobacter, Bacilli, Lactobacillus, and Coprobacillus were raised and Bacteria from Dorea, Clostridium, and Peptostreptococcaceae were decreased. | Tian et al., 2018 |
|  | Polysaccharide, dietary fiber, vitamins C,B12, folate, ergothioneine, poly-phenols, Lectin | Induced by streptozotocin (STZ) in SD rats | For 4 weeks, 200 mg/kg was administered orally. | - Plasma glucose levels are lower, triglyceride levels are lower, plasma total cholesterol is lower, and low density cholesterol is lower. ALT, AST, TGs, and TC levels are all lower. - Increase islet cell insulin and glucagon secretion, as well as glucose uptake. - High fiber inhibits the activity of digestive enzymes, whereas lectin-like molecules induce the production of insulin and glucagon. | Jeong et al., 2010 |
| *Agaricus blazei* Murrill | ABMP | For 20 weeks, Wistar rats were given either a control diet (CD) or a high-fat diet (HF) with or without ABM supplementation. | 25 mg/kg | - The HF diet resulted in more body weight increase and fat mass than the CD diet. In HF-fed rats, hyperleptinemia and insulinemia, as well as insulin resistance and glucose intolerance, were all seen. Inflammatory markers were also seen in the visceral adipose tissue of HF-fed rats. - ABM supplementation prevented HF rats against body weight gain and all other diseases associated with the trial. This was not due to reduced food intake; in fact, whether supplemented with ABM or not, HF rats consumed much more food than controls. The gut microbiota composition did not alter when HF was supplemented with ABM. - Surprisingly, ABM supplementation boosted energy expenditure as well as locomotor activity, which might explain why it protect against diet-induced obesity.In the jejunum of ABM-treated rats, there is also a decrease in pancreatic lipase activity, implying a reduction in fat absorption. | Vincent et al., 2013 |
|  | β-glucan | In Sprague–Dawley rats, streptozotocin (STZ) was induced | 6 weeks of treatment with 2% Agaricus β-glucans and 2% oligosaccharides (AO). | - Compared to control mice, diabetic mice given a 20 mg ml-1 dose for 5 weeks lost weight and had higher pancreatic insulin production. - Both β-glucans and AO had anti-hyperglycemic, anti-hypertriglyceridemic, anti-hypercholesterolemic, and anti-arteriosclerotic action in diabetic rats, showing that they had anti-diabetic effect generally. | Kim et al., 2005 |
|  | β-glucan | Treatment of male Sprague-Dawley rats with streptozotocin (STZ). | 7-day oral therapy with dosages ranging from 50–200 mg/kg/body weight | - When compared to control mice, a 20 mg ml-1 dose for 5 weeks lowered body weight and boosted pancreatic insulin production in diabetic mice. | Yang et al., 2014 |
| *Agaricus brasiliensis*  Fr. and *Ganoderma lucidum* (Curtis) P. Karst.  “[*Ganoderma orbiforme* (Fr.) Ryvarden]” |  | Male SD rats were stimulated with streptozotocin (35 mg/kg, i.v.). | Doses of 1.0 and 2.0 g/kg/body weight were given orally for four weeks. | - Antioxidant enzymes including GSH, CAT, SOD, GSH-Px, as well as GSH-R were increased, whereas blood glucose was reduced. The levels of LPO and TBARS were both reduced. | Vitak et al., 2017 |
|  | Mycelia powder | In rats, diabetic streptozotocin (STZ, 50 mg/kg) was induced. | For 14 days, a dosage of 1 g/kg was taken orally | - The physiological balance of sialylation and desialylation processes was influenced, the ion charge on red blood cell surface membranes had been controlled, the aggregation qualities were normalized, and the structural recovery of oligosaccharide chains of erythrocyte membrane surface glycoconjugates was increased. | Vitak et al., 2015 |
|  | Powdered mushrooms | In regular and experimental diabetes circumstances, the L-arginine/nitric oxide (NO) system and rat leukocyte apoptosis | A dosage of 1 g/kg/body weight should be taken orally. | - Reduced NO synthase activity, and also positive corrections to the L-arginine/NO system and the ratio of p53 and Bcl-2 proteins in white blood cells, and even a reduction in the apoptotic index. | Yurkiv et al., 2015 |
| *Agaricus campestris*  L.  “[*Agaricus bisporus* (J.E. Lange) Imbach,]” | Aqueous extract of fruiting body, Boiling water | Streptozotocin-induced hyperglycemia in diabetic mice | dosages of 62.5 g/kg | - Hyperglycemia was lower, and glucose transport was better. In an *in vitro* investigation, insulin release from glucose-responsive BRIN and BD11 cells increased when the extraction dosage was raised. | Gray and Flatt 1998 |
| *Agaricus subrufescens*  Peck | β-glucans and enzymatically produced oligosaccharides | diabetic rats provoked by streptozotocin (STZ) | Oral treatment of 1 g kg^−1^/d^−1^ | - In type 2 diabetes mellitus, higher adiponectin levels reduced insulin resistance. | Niwa et al. 2011 |
| *Agrocybe chaixingu* | Polysaccharide | Male ICR mice were made diabetic using streptozotocin (STZ, 120 mg/kg). | 7-day oral therapy at a dosage of 10 mg/kg | - Experimental model DNA fragmentation is prevented by sodium nitropruside. - In a mouse model, improve pancreatic β-cell resistance to STZ destruction. - *Agrocybe chaxingu* polysaccharide decreased NO production and iNOS expression in RINm5F cells in a dose-dependent manner. - *Agrocybe chaxingu* polysaccharide lowered iNOS expression and blood glucose levels significantly in STZ-induced diabetic mice. | Lee et al., 2010 |
| *Agrocybe cylindracea*  (DC.) Maire  “[*Cyclocybe parasitica* (G. Stev.) Vizzini,]” | From a hot-water extract of the fruiting bodies, a glucan (AG-HN1) and a heteroglycan (AG-HN2) were recovered. | Diabetes was generated in mice using streptozotocin (STZ, 100 mg/kg). | In experimental mice, a dosage of 50 mg/kg was given orally for three weeks. | - AG-HN1 was shown to have a greater hypoglycemic effect than AG-HN2. | Kiho et al. 1994 |
| *Armillaria mellea*  (Vahl) P. Kumm*.*  “[*Armillaria novae-zelandiae* (G. Stev.) Boesew.,]” | At 80°C, polysaccharide was extracted using 0.5 M NaOH/NaBH4. | HFD (Research Diets, D12492) is a 60 percent kcal percent fat diet. Sprague Dawley rats, males | For 35 days, oral dosages of 50, 100, and 200 mg/kg/day were given. | - AAMP reduced serum triglyceride levels by increasing LPL levels and the expression of two important lipases, ATGL and HSL. AAMP also decreased fat accumulation in the liver by reducing SREBP-1c expression specifically. - The advantage of AAMP in HFD/DEX-induced insulin resistance was connected to its lipid metabolism regulation, according to these findings. | Yang et al., 2019 |
| *Antrodia camphorata*  (M. Zang & C.H. Su) Sheng H. Wu, Ryvarden & T.T. Chang | Dehydroeburicoic acid | Male C57BL/6J mice were given the drug streptozotocin (55 mg/kg) to develop diabetes. | 28-day oral therapy with dosages of 10, 20, and 40 mg/kg | - Increase the level of GLUT 4, AMPK, PPAR-α and decrease glucose levels, Fatty acid synthase, m-RNA levels of hepatic adipocyte fatty acid binding protein, Glycerol-3 phosphate acyl transferase (GPAT). | Kuo et al., 2015 |
| *Antrodia cinnamomea*  T.T. Chang & W.N. Chou | ACP | Male C57BL/6J mice were fed a high fat diet for 8 weeks. | Per day, dosages of 0.009 and 0.09 g/kg 1 were administered. | - Firmicutes/Bacteroidetes ratio has been improved abd reduced fat accumulation TC, IL-1β, IL-6, | Chang et al., 2018 |
|  | Polysaccharide | α-glucosidase inhibitory activity | 50 µL | - 25R-antcin K, dehydrosulphurenic acid, 25S-antcin B, 25R-antcin B, dehydroeburicoic acid, and eburicoic acid were identified as the eight chemicals isolated from ACFB. Acarbose had a smaller impact than ACFB extract and its recognized components. | Huang et al. 2018 |
|  | 95 % ethanol | Diabetes was developed in Sprague Dawley rats using streptozotocin (STZ, 65 mg/kg/body weight). | For 5 weeks, dosages of 385, 770, and 1540 mg/kg/body weight were administered orally. | - Increased glucose tolerance lowered total cholesterol and triglyceride levels in diabetic mice. Reduce plasma membrane LPO and RAGE expression while improving SOD performance. | Johnson et al., 2019 |
| *Antrodiella albocinnamomea*  Y.C. Dai & Niemelä | AntroalbolH | cellular glucose uptake in 3T3-L1 adipocytes and L6 myotubes in mice |  | - According to the results, this impact is connected to LKB1-mediated AMPK Thr-172 phosphorylation. LKB1 is phosphorylated specifically at Thr-189 by antroalbol H, which changes its subcellular location. - Finally, antroalbol H treatment enhances glucose transporter type 4 (GLUT4) translocation to the plasma membrane. We conclude that antroalbol H increases LKB1 phosphorylation at Thr-189, which leads to AMPK activation, indicating this residue as a potential target for improving glucose absorption and implying that antroalbol H might be efficacious in the management of hyperglycemia. | Wang et al., 2019 |
| *Lentinus edodes*  (Berk.) Singer | Polysaccharide | Male SD rats had their pancreas removed using 1 g dextrin/kg/body weight. | 0.5-1 g/kg/body weight oral therapy | - Serum glucose, food consumption, body weight, and OGTT all decreased. | Yang et al., 2018 |
| *Astraeus hygrometricus*  (Pers.) Morgan | Fruiting body ethanolic extract and polysaccharide | Alloxan monohydrate (150 mg/kg/body weight) was used to cause diabetes in Swiss albino mice. | For 28 days, oral dosages of 250, 500, and 1000 mg/kg were administered. | - Blood glucose, plasma glucose, total cholesterol, as well as triglyceride levels were all lowered. | Biswas and Acharya (2013); Kim et al., 2007 |
| *Auricularia auricula-*judae (Bull.) Quél*.* “[*Auricularia cornea* Ehrenb.,]”  and *Auricularia polytricha*  (Mont.) Sacc. | Polysaccharide that dissolves in water | Male mice were given a 1g/kg glucose/body weight dosage to create elevated glucose levels. | For 28 days, an FA diet (30 g FA/kg) was administered orally. | - Increase hepatic glycogen levels while lowering plasma and urine glucose levels. The total body weight, urine glucose, blood glucose, serum insulin, and hemoglobin A1c levels were all lowered. | Yuan et al., 1998 |
|  | crude polysaccharide and AAP | Streptozotocin (STZ, 30 mg/kg, i.v.) was used to cause diabetes in male SD rats for three days. | 4 weeks of oral therapy with dosages of 100 and 400 mg/kg | - HbA1c was lowered by 80.1 percent due to NF-κB-related signaling and antioxidative system regulation. - When IFN-γ levels were elevated, TNF-α, IL-2, and NF-κB levels were reduced. - Plasma glucose, as well as blood urea nitrogen (BUN) and uric acid (UA) in serum and protein content in urine, were drastically lowered by altering the anti-oxidative system and nuclear factor kappa B-related proteins. | Hu et al., 2017 |
|  | Polysaccharide | Mice with type 2 diabetes mellitus caused by streptozotocin | 100 mg/kg/day | - The oral administration of AAP at a dose of 200 mg/kg lowered fasting blood glucose and fasting serum insulin levels in an oral glucose tolerance test. TC, TG, HDL-C, and LDL-C were lowered in a homeostatic model assessment of Insulin resistance index, and histological examination revealed significant hypoglycemic activity. | Chen et al., 2021 |
|  | Polysaccharides from un-smashed or smashing and sieving (through a 10-mesh sieve) AA and AP (termed as AAP/AAP-10 and APP/APP-10) via scalable processes (water extraction, ethanolic precipitation and deproteinization). | streptozotocin-induced oxidative stress and diabetes-related changes in Sprague-Dawley mice | 100, 300 mg/kg body for 4 week | - The ability of AAP and APP to prevent streptozotocin-induced oxidative stress and diabetes-related changes in body weight, fasting blood glucose, serum insulin, proinflammatory mediators and cytokines, oxidative stress-related products, and antioxidant enzymes in mice. Different molecular weights and monosaccharide molar ratios of APP and AAP might be used to treat diabetes, with a low dosage (100 mg/kg/day) being most beneficial. - APP outperformed AAP at the same dosage, and AAP-10/APP-10 looked to be somewhat more helpful than AAP/APP. One reason for these anti-diabetic effects might be the NF-κB and associated signaling pathways. AP is a useful source of functional polysaccharides since it is less costly than AA. | Xiang et al., 2021 |
|  | Dried mycelia powder |  |  | - The levels of glucose, total cholesterol, and triglycerides in the blood were all much lower. | Kim et al. 2007 |
| *Calvatia gigantea*  (Batsch) Lloyd | Butanol-Aqueous, Dichloro-methane, Ethyl-acetate | The activity of α-amylase was measured *in vitro*, and alloxan monohydrate (120 mg/kg body weight) was utilized to induce diabetes in Wistar Albino rats *in vivo*. | Oral therapy for 7 days with dosages of 200 and 400 mg/kg | - The enzymes α- amylase and glucosidase were both inhibited. Blood glucose levels were lowered by 28% in an *in-vivo* test. All fractions have inhibitory effect against -amylase *in vitro*. | Ogbole et al., 2019 |
| *Catathelasma ventricosum*  (Peck) Singer | Selenium polysaccharide | Streptozotocin (STZ, 150 mg/kg, i.p.) was used to cause diabetes in male ICR mice. | For four weeks, a 0.2 g/kg oral therapy was administered. | - The levels of blood glucose, TC, TG, LDL-C, as well as HDL-C were all lowered. In diabetic rats, it protect the liver, kidneys, and pancreas while also demonstrating hypolipidemic activity. | Liu et al., 2017 |
| *Cerrena unicolor*  (Bull.) Murrill | Extracellular polysaccharide | In Sprague-Dawley rats, diabetes was produced by injecting streptozotocin (STZ, 50 mg/kg body weight) intramuscularly. | 7 days of oral administration of 100 mg/kg body weight | - Polysacchrides have drastically reduced blood glucose levels. | Yamac et al. 2009 |
| *Chroogomphus rutilus*  (Schaeff.) O.K. Mill. | Polysaccharide | Streptozotocin (40 mg/kg, i.p.) was used to cause diabetes in male SD rats. | For four weeks, patients were given dosages of 1.0 and 2.0 g/kg/body weight. | - Treatment with polysaccharides lowered -glucosidase and blood glucose levels while raising SOD and GSH-Px. The levels of MDA, TC, TG, LDL-C, HDL-C, and MTT all decreased. | Zhang et al., 2017 |
| *Coprinus comatus*  (O.F. Müll.) Pers. | mycelium polysaccharides | In mice, streptozotocin (STZ, 30 mg/kg) was used to cause diabetes. | For 90 days, oral dosages of 100, 200, and 400 mg/kg/body weight were provided. | - CMP improved insulin resistance as well as energy metabolism in DN mice, and also significantly reducing kidney dysfunction and alleviating renal oxidative stress and inflammation, according to the results. According with western blot results, CMP also reversed kidney damage through altering the PTEN/PI3K/Akt and Wnt-1/-catenin pathways. - According to the structural analysis, the anti-diabetic nephropathic actions of CMP could have been attributed to the major monosaccharide-compositions of galactose, α-pyranose conformation, and acceptable molecular weights of 495.8 kDa. | Gao et al., 2021 |
|  | Polysaccharide | Alloxan was used to cause diabetes in Rattus norvegicus rats. | 14-day oral therapy at a dosage of 500 mg/kg/day | - Oral polysaccharide therapy produced greater effects, such as a 12.33 percent reduction in BGL (32.6%), reduction in MDA (6.35%), reduction in HbA1c (10.57%), reduction in plasma insulin and increased SOD levels. | Ratnaningtyas et al., 2019 |
|  | CCPF and CCPP | Diabetes induced in ICR mice | For 17 days, an oral dosage of 500 mg/kg was administered. | - Treatments with CCPF and CCPP lowered blood glucose levels. | Liu et al., 2013 |
|  | 4,5- Dihydroxy-2-methoxybenzaldehyde | *In vitro* inhibition of non-enzymatic glycosylation for *in vivo* alloxan-induced diabetes in Male Kunmin mice. | For 28 days, an oral dosage of 80 mg/kg was administered. | - Inhibitors of the non-enzymatic glycosylation (NEG) process. The levels of fructosamine, triglycerides, and total cholesterol are lowered. Improves glucose tolerance while keeping blood glucose low. | Ding et al. 2010 |
|  | Polysaccharide fractions (ethanol based) | Male ICR mice developed diabetes after being exposed to alloxan (65 mg/kg). | Oral administration of dosages of 500 and 1000 mg/kg in 12 minutes and injection of the same dosage for 21 days | - Immune activation might play a role in hypoglycemic activity. | Zhou et al., 2015 |
| *Cordyceps militaris*  (L.) Fr. | polysaccharide (α and β) | Streptozotocin (25 mg/kg) was used to cause diabetes in Sprague Dawley rats. | For four weeks, animals were given oral treatments of 0.5 and 1 g/kg/day. | - In people with type 2 diabetes, increase insulin resistance whilst still preserving the liver, kidneys, and pancreas. Dyslipidemia is now less of an issue. - The levels of FBG, TG, PK, TC, BUN, CRE, UA, urine protein, NAG, and MDA changed. The concentrations of SOD and GSH-Px have risen. | Liu et al., 2016 |
|  | Polysaccharide | Streptozotocin (STZ, 60 mg/kg, i.p.) was used to cause diabetes in Wistar mice. | Doses of 100 and 400 mg/kg were given orally for four weeks. | - FBG, serum insulin, AST, OGTT, ALT, CRE, BUN, TC, LDL-C, HDL-C, renal, hepatic, and pancreatic GSH-Px, SOD, CAT, and LPO levels were all changed after polysaccharide administration. | Zhao et al., 2018 |
|  | Polysaccharide | HFD + STZ (60 mg/kg, i.p.) with nicotinamide (180 mg/kg, i.p.) were used to develop diabetes and hyperglycemia in C57BL/6J mice. | For 8 weeks, a 360 mg/kg/body weight dosage was administered orally. | - Oral polysaccharide delivery was reported to lower OGTT, FBG, CRE, IPITT, TGF-1, AGEs, TG, TC, LDL-C, and HDL-C levels. | Yu et al., 2016 |
|  | Lipopolysaccharide and Cordycepin | RAW264.7 cells (2×10^5^  cells/well) | 100 μl | - Cordycepin has been found to inhibit NO production caused by LPS when taken orally. Cordycepin has the capacity to influence genes involved in type 2 diabetes (RANTES, PPAR-γ, and 11 HSD1). Cordycepin therapy inhibited LPS-induced NF-κB p65 in RAW 264.7 cells in an *in vitro* research. | Shin et al., 2009 |
|  | From the fruiting body, a polysaccharide-rich fraction was isolated. | Streptozotocin (STZ, 40 mg/kg/body weight) was used to cause diabetes in rats. | 10 mg/kg/body weight orally administered | - This function promotes both insulin-like and insulin-releasing actions. When blood glucose levels rise at a slower pace, hypoglycemia occurs. | Zhang et al. 2006 |
| *Cordyceps sinensis*  (Berk.) Sacc. | CSP-1 polysaccharide produced from Cordyceps mycelia. | Streptozotocin (60 mg/kg/body weight, i.p.) was used to cause diabetes in male Sprague Dawley rats. | Doses of 200 and 400 mg/kg/body weight were administered orally for 7 days. | - The basal glucose levels of normal mice were also not significantly different. CSP-1 reduced blood glucose levels by 12.073.2 % and 22.574.7 % in normal mice, correspondingly. - When administered at a dosage of more than 200 mg/kg body weight daily for 7 days, CSP-1 dramatically lowered blood glucose levels in STZ-induced diabetic rats and alloxan-induced diabetic mice, as well as increased serum insulin levels in diabetic rats. CSP-1 combined hypoglycemic properties increased the amount of circulation insulin in diabetic rats. | Li et al., 2006 |
| *Cordyceps sinensis*  (Berk.) Sacc., *Omphalia lapidescens*  *(Horan.)* Cohn & J. Schröt., and *Tricholoma mongolicum*  S. Imai | Polysaccharide | Streptozotocin (STZ, 40 mg/kg, i.p.) was used to cause diabetes in Wistar rats. | Doses of 10 and 100 mg/kg were given orally for four weeks. | - FBG and PBG levels were reduced after oral polysaccharide treatment. | Zhang et al., 2006 |
|  | Polysaccharide | Streptozotocin (STZ, 40 mg/kg, i.p.) was used to cause diabetes in Wistar male rats. | Doses of 500 and 2000 mg/kg/body weight were administered orally for three weeks. | - Polysaccharide treatment lowered PK, FBG, TC, TG, UA, BUN, CRE, and urine protein levels. In the meanwhile, GSH-Px and SOD levels have increased. | Dong et al., 2014 |
| *Paecilomyces tenuipes*  (Peck) Samson  (*Cordyceps takaomantana*) | Fruiting body extracts of 4-β- acetoxyscirpendiol (ASD), Paecilomyces tenuipes, and 4-βbeta acetoxyscirpendiol (ASD) | In Xenopus oocytes, the Na+/glucose transporter-1 (SGLT-1) was expressed. |  | - Treatment of polysaccharides reduced blood sugar in the circulatory system, researchers used specific inhibitors of Na+/glucose transporter-1 (SGLT-1) | Yoo et al., 2005 |
| *Coriolus versicolor*  (L.) Quél. | Ternatin | male KK-Ay mice | Treatment concentration of 8.5 or 17 nmol/day for four weeks were used. | - In KK() mice and the 3T3L1 cell line, ternatin administration changed the regulation of PPAR-γ, SERBP-1C, FAS, and ACC, suppressing hyperglycemia and fatty acid synthesis. | Kobayashi et al., 2012 |
|  | Polysaccharide | In the *in vitro* investigation, myoblasts (L6 cells) were employed; in the *in vivo* study, rats were given high-fat diets and STZ to induce diabetes and hyperglycemia. | Oral treatment of doses at 25, 50, 100 mg/kg | - Polysaccharide treatments had outstanding antiinsulin resistance effects. The alteration in gene and protein expression levels in the PI3K/Akt and p38 MAPK pathways revealed that LY29004, a PI3K inhibitor, and SB203580, a p38 MAPK inhibitor, added to the evidence. | Xian et al. 2018 |
| *Phellinus rimosus*  (Berk.) Pilát | Polypore medicinal mushroom with 70% ethanol (v/v) | Alloxan (140 mg/kg/body weight) was used to cause diabetes in male Wistar albino rats. | Doses of 50 and 250 mg/kg/body weight/day were administered orally. | - Giving alloxan-induced diabetic rats 50 and 250 mg/kg body weight/day for 10 days resulted in a significant dose-dependent hypoglycemic effect. When compared to the control group, *P. rimosus* (250 mg/kg) had a substantial hypoglycemic effect, which peaked 90 minutes after the glucose challenge in the oral glucose tolerance test. - Extract was used to examine the antioxidant status of the pancreas, liver, as well as kidney. The activities of GPx, , CAT, SOD, as well as GSH were increased in pancreatic, hepatic, and renal tissues of diabetic rats administered the extract. The high amount of lipid peroxidation in diabetic rats has been shown to revert to near-normal levels in groups given the extract. | Rony et al., 2013 |
|  | Extract | In diabetic rats, streptozotocin (STZ) was used to produce diabetes. | For 30 days, oral dosages of 50 and 250 mg/kg were administered. | - Improved antioxidant status and a reduction in TBARS were seen in pancreas, liver, and kidney tissues, indicating probable insulinogenic and antioxidant mechanisms. | Rony et al., 2015 |
| *Flammulina velutipes*  (Curtis) Singer | Monosaccharide; Al-RPS, Ac-RPS, and En-RPS | Streptozotocin (STZ, 80 mg/kg) was administered three times intraperitoneally to male Kunming mice to develop diabetes. | Doses of 200, 400, and 800 mg/kg were given orally for 15 days. | - Oral monosaccharide treatment lowered blood CRE, BUN, ALB, GLU, and renal MDA levels; renal CAT, SOD, and GSH-Px levels were raised, alleviating kidney damage. | Lin et al., 2016 |
| *Ganoderma pfeifferi*  *Bres.* and *Ganoderma resinaceum* Boud. | ethanolic and water extracts | Alloxan (100 mg/kg/body weight, i.p.) was used to cause diabetes in Wistar rats. | 5 days of oral therapy at a dosage of 65 mg/kg/body weight | - In the oral glucose tolerance test (OGTT) and histological assessment of the pancreas and liver, G. pfeifferi extracts lowered blood glucose levels. Extracts of G. pfeifferi have been shown to protect pancreatic tissue. | Rašeta et al., 2020 |
| *Ganoderma lucidum*  (Curtis) P. Karst.  “[*Ganoderma orbiforme* (Fr.) Ryvarden]” | F31 | In Kunming mice, diabetes was produced using streptozotocin and a high-fat diet. | For 7 days, 50 mg/kg/body weight was administered orally. | - AMPK, glucose transporter 4 (GLUT4), fructose-1,6-bisphosphatase, glycogen phosphorylase, and phosphoenol-pyruvate carboxykinase enzymes were all raised after oral administration of F31 polysaccharide, whereas phosphoenol-pyruvate carboxykinase was decreased. | Xiao et al., 2017 |
|  | GLP peptides | Diabete was induced by streptozotocin in  Wistar rats | Oral treatment of dose at 150 mg/kg/body weight for 8 weeks | - LDL-C, TG, TC, and free fatty acids were reduced by the GLPP therapy, but Allobaculum, Psychrobacter, Blautia, and Roseburia were increased. - However, the GLPP therapy reduced hepatic lipid metabolism-related mRNA expression and increased total bile acid excretion in the feces (BAs). These results suggested that GLPP may help with lipid metabolic diseases by regulating genes involved in hepatic lipid and cholesterol metabolism and changing gut microbiota composition. | Lv et al., 2019 |
|  | Polysaccharides (Gl-PS) | Diabetes was induced by the induction of streptozotocin (STZ, 65 mg/kg/bw.) in Sprague-Dawley rats | Orally administred dose of 200 mg/kg/body weight for 7 days | - After eight weeks of oral therapy with Gl-PS at dose of 200 mg/kg, plasma concentrations of fasting glucose, triacylglyceride, total cholesterol, and NO were significantly lower. Pancreatic SOD, CAT, and GPx were all considerably increased in the Gl-PS. - According to histopathology studies, Gl-PS exhibited a protective effect on β-cells. When compared to the diabetic control group, Bcl-2 and PDX-1 mRNA expression in the pancreas was up-regulated, while Bax, iNOS, and Casp-3 mRNA expression was down-regulated. | Zheng et al., 2012 |
|  | GLP, Ganoderans A, Ganoderans B, F31 , Gl-PS, PSG-1 | Diabetes was induced by the induction of streptozotocin (STZ, 65 mg/kg/bw.) in Sprague-Dawley rats | Oral treatment of dose at 100 mg/kg/body weight for ten days | - After GLP therapy, MAPK pathway modulator, Bcl-2 (anti-apoptotic protein), and PDX-1 pathway inhibitors prevent apoptosis in β-cells while increasing their numbers. | Zheng et al., 2019 |
|  | Gl-PS | Diabetes was induced in albino Swiss mice | Oral treatment of doses at 50, 100 and 200 mg/kg/body weight | - *In vitro* and *in vivo*, Gl-PS therapy scavenges free radicals while reducing NF-κB activity protect against alloxan-induced pancreatic islet damage. | Zhang et al., 2003 |
|  | GLPs | Diabetes was induced in streptozotocin (60 mg/kg/body weight) in Kunming mice | Oral treatment of doses at 50, 100 mg/kg/body weight for 7 days | - Many key enzymes involved in gluconeogenesis and/or glycogenolysis had their mRNA expression reduced by Gl-PS therapy (GP, FBPase, PEPCK, and G6Pase). | Xiao et al., 2012 |
|  | FYGLn | Diabetes was induced in streptozotocin (60 mg/kg/body weight) in C57BL/6 mice and C57BL/6 db/db mice | Oral treatment of doses at 75, 250, 450 mg/kg | - Improves insulin sensitivity and reduce hepatic glucose production while boosting adipocyte and skeletal muscle glucose excretion in late-stage diabetes. | Pan et al., 2013 |
|  | GLP1 | Diabetes was induced by the induction of Alloxan in mice | Oral administration of dose at 200 mg/kg/body weight for 21 days | - Oral treatment of GLP1 reduced BG levels, meanwhile increased GPx and SOD activity, lower MDA levels, and reduce pancreatic pathologic lesions. | Liao et al., 2015 |
|  | G1-PS | In SD rats, diabetes was induced by an intravenous dose of streptozotocin (65 mg/kg). | For 8 weeks, take a dosage of 20 mg/kg/body weight orally. | - Apoptosis of pancreatic β-cells is avoided by oral administration of G1-PS, and β -cell regeneration is enhanced. | Zheng et al., 2012 |
|  | G1-PS | Male C57BL/6 mice were given an intraperitoneal administration of streptozotocin (STZ, 60 mg/kg) for 5 days to develop diabetes. | Doses of 10, 50, and 250 mg/kg were given orally. | - G1-PS decreased mitochondrial oxidative stress, p66Shc, as well as cutaneous MnSOD nitration when given orally. | Tie et al., 2012 |
|  | GLP-1 and GLP-II | In SD rats, diabetes was induced by an intravenous dose of streptozotocin (30 mg/kg) for 3 days | Doses of 200, 400, and 800 mg/kg were given orally for 16 weeks | - As a consequence of decreased AGE levels and improved antioxidant enzyme activity, cardiac collagen crosslinking was reduced after orally administered with GLP-1 and GLP-II and increase SOD, CAT, as well as GSH-Px activity, as well as lower BG and HbA1c levels and increase blood insulin levels. - GLP-1 and GLP-II dosing resulted in Bcl-2 (anti-apoptotic protein) and PDX-1 pathway modification, hypoglycaemic and hypolipidaemic effects, inhibiting β-cell apoptosis and increasing β-cell numbers, and MAPK pathway modification. | Meng et al., 2011 |
|  | GL-PS | Streptozotocin (STZ, 100 mg/kg) was injected intraperitoneally in C57BL/6J mice with diabetes for two days. | Doses of 125 and 250 mg/kg were given orally for 8 weeks | - Metabolic disorders, oxidative stress, and renal dysfunction connected to renal lesions were improved after treated GL-PS orally. | He et al., 2006 |
|  | GL-PS | In SD rats, diabetes was induced by an intravenous dose of streptozotocin (35 mg/kg) for 3 days | Doses of 200, 400, and 800 mg/kg were given orally for 16 weeks | - The effects of GL-PS on oxidative stress and AGE levels, as well as cardiac fibrosis, were modified after oral treatment. | Li, 2011 |
|  | GL-PS | SD mice were given an intraperitoneal administration of streptozotocin (STZ, 30 mg/kg) for a week to develop diabetes. | SD mice were given an intraperitoneal administration of streptozotocin (STZ, 30 mg/kg) for a week to develop diabetes. | - The antioxidant enzyme activity of GL-PS was increased, while myocardial CTGF was decreased after oral treatment. | Qiao et al., 2014 |
|  | Polysaccharides Glc, Gal and Man | For four weeks, male Sprague-Dawley rats were given an induction HFD and STZ (35 mg/kg). | 4 weeks of oral therapy at 400 mg/kg/day | - Ruminococcus, Coprococcus, and Aerococcus reduced, whereas Blautia, Dehalobacterium, and Lactococcus increased. FBG, FINS, HOMA-IR, serum TC, TG, and LDL-C all decreased, whereas HDL-C, liver SOD, CAT, GSH-Px, and butyrate all increased. The words MDA, serum 1, IL-6, and CRP all refer to the same phenomenon. The restoration of normal gut flora decreases systemic inflammation and improves serum lipids and oxidative stress. | Chen et al., 2019 |
|  | 3β,24E)-lanosta-7,9(11),24- trien-3,26-diol (ganoderol B) |  |  | - Showed strong α-glucosidase inhibition activity. | Fatmawati et al. 2011 |
|  | Aqueous extraction | kk mice |  | - Oral therapy lowers blood glucose levels and has anti-diabetic properties. | Manohar et al., 2002 |
|  | GLP | STZ induction caused diabetes in Wistar Albino rats. |  | - Corynebactrium, Ruminococcus, and Proteus were all up, while Parabacteroides, Blautia, and Bacteroides were down. Insulin and FBG levels lower. | Chen et al., 2019 |
| *Ganoderma resinaceum*  Boud. | Polysaccharide | Alloxan induction caused diabetes in Wistar Albino rats for 5 days |  | - Elevated the antioxidant activity | Rašeta et al., 2020 |
| *Grateloupia lithophila* | crude polysaccharide and rhamnose-enriched polysaccharide | Diabetes was induced by the induction of streptozotocin (STZ)-in Wistar rats | Oral treatment of dose at 100 mg/kg/day for one month | - Total cholesterol (TC), triglycerides (TGs), low-density lipoprotein (LDL), and very-low-density lipoprotein (VLDL) contents in the blood of crude polysaccharide- and rhamnose-enriched polysaccharide-treated rats decreased, but HDL increased. - Superoxide dismutase (SOD) and glutathione peroxidase (GPx) levels increased in the livers, kidneys, and pancreases of crude polysaccharide- and rhamnose-enriched polysaccharide-treated rats, whereas malondialdehyde (MDA) levels decreased. - According to immunohistochemistry, the restoration of normal cellular size of the islets of Langerhans and the rebirth of β-cells was larger in the body area of the pancreas than in the head and tail regions. Diabetic rats given crude polysaccharide or rhamnose-enriched polysaccharide produced normal blood glucose and insulin, as well as reversed cholesterol levels and enzyme activity. | Seedevi et al.,2020 |
| *Grifola frondosa*  (Dicks.) Gray | Polysaccharide | Diabetic caused by diet streptozotocin 200 mg/kg for 4 weeks | Oral treatment of 100 mg/kg for 4 weeks | - In diabetic rats, it lowers P-NF-κBp65 and P-IκB expression in the kidneys, as well as BUN, Scr, and NAG levels in the blood and excessive albumin in the urine. Normalize inflammation-regulating factors in diabetic rats and have an influence on insulin production. - Inhibited IL-2 and TNF-α; reduced ROS; raised SOD, Gpx, CAT, and MDA; and inhibited IL-2 | Kou etal., 2019 |
|  | Polysaccharides derived from extract or mycelia |  | Oral administration of 1 g/kg- for two weeks | - Polysaccharide therapy decreased inflammation by downregulating IFN-γ, IL-4, and IL-6 IL-4 production while drastically boosting TNF-α production. | Chen et al., 2015 |
|  | Novel polysaccharide from *Grifola frondosa* | Streptozotocin/high fat diet-induced (type 2) diabetes was produced in mice. | 28-day oral therapy with 75 and 150 mg/kg/body weight | - GFP-N activates insulin receptor substrate 1, phosphatidylinositol-3-kinase, and glucose transporter 4 in diabetic mice livers whereas inhibiting c-Jun N-terminal kinase 1/2, causing hypoglycemic effects. | Chen et al., 2019 |
|  | Ethanol extract derived polysaccharides | C57BL6/J mice | Diet comprising 0.2% for 15 weeks | - Because the PPAR-γ signaling pathway was active in diabetic mice, oral polysaccharide therapy lowered TC and enhanced glucose absorption. | Aoki et al., 2018 |
|  | GFP | Diabetes was induced with high fat diet (HFD, 45% calories from fat, MD12032) in Wistar rats | Oral administration of dose at 400 mg/kg/day for 8 weeks | - Clostridium-XVIII and Turicibacter were found to be more common, whereas Helicobater, Barnesiella, Parasutterella, and Flavonifracter were found to be less common. Lipid accumulation in the liver was decreased when TG, TC, and free fatty acids were reduced. | Li et al., 2019 |
|  | α-glucan |  | Oral administration of 100 and 300 mg/kg | - Oral polysaccharide therapy reduced inflammation and lowered triglycerides, cholesterol, and free fatty acids. | Lei et al., 2012 |
|  | Glc, Ara and Man |  | Oral treatment of 75 and 150 mg/kg/day, 4 weeks | - Elevated glucose tolerance, insulin signaling pathways (IRS1/PI3K/GLUT4), as well as reduced FBG, HbA1c, and HOMAIR; liver and kidney damage after oral polysaccharide therapy. | Chen et al., 2019 |
|  | Glc, Man and Gal | Diabetes was produced by combining a 4-week HFD with STZ (90 mg/kg). Male mice from Kunming | 4 weeks of orally administered of 300 and 900 mg/kg/day | - Alloprevotella, Jeotgalicoccus, Sporosarcina, Anaerovorax, Bacteroides, and Jeotgalicoccus were decreased. The amount of LPS and gram-negative bacteria in the body was lowered. - Enhanced glucose tolerance, reduced FBG, decreased serum and liver lipids (TG, FFA, TC). Also reduction of hepatic glucose metabolism (G6Pase, FBPase, PEPCK, GP); hepatic lipid synthesis (SREBP-1c, Cd36, ACC-1HMGCR). Additionaly increased CYP7A1, acetate, BSEP, propionate, and butyrate, BSEP and CYP7A1, - Polysaccharides seem to alter the composition of the gut microbiota, resulting in improved intestinal barrier integrity, gut immunological homeostasis, increased gut hormone production, and altered bile acid profiles, all of which contribute to diabetes improvement. | Guo et al., 2020 |
|  | Glucose, mannose galactose, xylose,arabinose, ribose, arabinose, xylose |  |  | - Polysaccharides seem to regulate fasting serum insulin and glucose levels, as well as the PI3-AKT pathway. | Xiao et al., 2015 |
|  | Polysaccharide |  |  | - Polysaccharides are perhaps crucial in managing blood sugar, renal function, and diabetic nephropathy. A high fibre content in the intestines may help to prevent blood glucose spikes by reducing glucose absorption. | Lo et al., 2008 |
|  | MT-α glucan | KK mice |  | - Increase the number of low affinity insulin receptors on the liver membrane in Kk mice to lower blood lipid levels. Improve insulin sensitivity and insulin resistance in the periphery. | Kim et al., 2005 |
|  | n-hexane derived polysaccharides |  |  | - Although glucosidase activity is severely decreased, α-amylase activity only moderately hindered. | Su et al., 2013 |
|  | Mushroom extracts rich in vanadium | Diabetes was induced by Alloxan with adrenalin in female Kunming mice | Oral treatment of doses at 0.18 mg/kg/d for 45 days | - Oral polysaccharide therapy lowers HbA1c levels in the blood. | Cui et al. 2009 |
|  | Alpha-glucan (MT-alpha-glucan) |  |  | - Insulin sensitivity and resistance have both been proven to be improved by α-glucan. | Hong et al., 2007 |
|  | Polysaccharides F2 and F3 | High-fat diet induced diabetic mice |  | - Enhanced insulin receptor phosphorylation, reduced insulin receptor substrate-1 (IRS-1) expression, including F3 activation of such PI3K/Akt pathway, with F2 suppression of protein tyrosine phosphatase 1B. | Xiao et al. 2015 |
|  | Glycoprotein extract derived SX fraction | For at least 3 weeks, 20 % w/w sucrose (Suc) | Oral administration of doses at 0.87, 1.75, 3.5 g/kg | - Improved glucose tolerance and increased sensitivity to exogenous insulin | Preuss et al. 2007 |
| *Grifola gargal*  Singer | Aqueous extract (hot) | Diabetes was induced by streptozotocin (STZ) in ICR mice |  | - On blood glucose for 5 weeks, G. gargal powder (GGP) in a high-molecular weight aqueous extracts (GGH) as well as a low-molecular weight hot water extract (GGL). A low molecular mass hot water extract may lower blood glucose and body fat levels in diabetic mice. | Harada et al., 2017 |
| *Hericium erinaceus*  (Bull.) Pers. | separated fractions of intracellular polysaccharide (HIPS) (HIPS1 and HIPS2) | Diabetes was induced by an induction of streptozotocin (STZ, 120 mg/kg) | Oral administration of doses at 200, 400, 600 mg/kg for two weeks | - Supplementing with HIPS1 and HIPS2 significantly lowered blood glucose (GLU) levels. Serum levels of aminotransferase (ALT), alkaline phosphatase (ALP), aspartate aminotransferase (AST), urea nitrogen (BUN), alanine, and creatinine (CRE) all fell. SOD, GSH-Px, and CAT enzyme activity were all enhanced. - HIPS1 beat HIPS2 in terms of antihyperglycemia and organic protection, perhaps because to HIPS1's presence of functional groups (-NH2, -COOH, and S=O), suggesting that H. erinaceus SG-02 might be used as a functional food and natural treatment to prevent diabetes and its consequences. | Zhang et al., 2017 |
|  | Methanol extract | male Kunming mice | 1 mg/kg | - Hypoglycemic effects, with blood glucose levels rising at a slower pace. | Wang et al. 2005 |
|  | Polysaccharides |  |  | - It was able to lower blood glucose levels, boost insulin production, and reduce the amount of glycogen stored in the liver. | Chaiyasut and Sivamaruthi, 2017 |
| *Hirsutella sinensis*  X.J. Liu, Y.L. Guo, Y.X. Yu & W. Zeng | HSP | Hyperglycemic was induced by an induction of High-fat diet (HFD)-fed mice. | Oral treatment of 20 mg/kg for 12 weeks | - Reduced inflammation and IR; increased *Parabacteroides goldsteinii* | Wu et al., 2019 |
| *Hypsizygus marmoreus*  (Peck) H.E. Bigelow | polysaccharides | Diabetes was induced by an intraperitonial induction of streptozotocin (STZ, 80 mg/kg) in Kunming strain mice | Oral administration of doses at 200, 400, 800 mg/kg, for 15 days | - Enzymatic (SOD, GSH-Px) and non-enzymatic (T-AOC)) activities were significantly increased in diabetic mice treated with these three polysaccharides, contents of LPO and MDA were remarkably reduced, and clinical parameters were observably mitigated. - The recovery was also confirmed by histological results. These results revealed that SCP, as well as its hydrolysates ESCP and ASCP, have substantial antioxidant effects and might be utilized as a potentially useful meal to avoid diabetes and the complications that come with it. | Liu et al., 2017 |
| *Hypsizygus ulmarius*  (Bull.) Redhead | Aqueous extract | Diabetes was induced by an intraperitonial induction of streptozotocin (STZ, 65mg/kg/ body weight) in rats | 50 mg/kg/body wt twice a day | - The gluconeogenic enzyme (glucose-6-phosphate, fructose 1,6 bis phosphate) activity in diabetic mice treated with STZ is decreased. | Meera et al., 2011 |
| *Inonotus obliquus*  (Ach. ex Pers.) Pilát | 3 β hydroxyl lanosta 8, 24-diene 21 al, Inotodiol, Trametenolic acid and Inotodiol | Culture broth |  | - It has anti α-amylase and chemical free radical scavenging activities. - Blood glucose levels that are lower have anti-hyperglycemic and anti-lipidperoxidative properties. | Lu et al., 2010 |
|  | Hexane, chloro- form,ethyl acetate, |  |  | - Anti-diabetic effects may exist in a restricted number of drugs. | Geng et al., 2013 |
|  |  | HepG2 cells and insulin-resistant HepG2 cells | Treatment concentration as 10, 20, 40, 80, and 160 µg/mL, for 24 and 48 h. | - Glucose levels are lower, and insulin absorption is higher. | Xue et al., 2018 |
|  | Inonotus obliquus polysaccharide |  |  | - The inhibition of activity by α-glucosidase and α-amylase was seen after polysaccharide treatment. | Wang et al., 2018 |
|  | Hot aqueous extraction |  |  | - Enhanced GLUT 4 expression in 3T3-L1 pre adipocyte cell | Joo et al., 2010 |
|  | Polysaccharides and polyphenols | Diabetes was induced by an intraperitonial induction of streptozotocin (STZ, 60 mg/kg) in Male Kunming mice | Oral administration of dose at 900 mg/kg for 4 weeks | - Increased hepatic glycogen levels and lowered insulin resistance; increased HDL-C; decreased TC, TG, and LDL-C levels; and decreased PI3K-p85, p-Akt, and GLUT4 levels. Polysaccharide inhibits dipeptidyl peptidase 4 (DPP4) enzymes and causes GLUT4 receptor translocation, according to studies.. | Wang et al., 2017 |
| *Lactarius deterrimus*  Gröger | Ethanolic extract |  |  | - Activating the CXCL12/Akt pathway, which preserves pancreatic cells and β-cell mass, improves hyperglycemic conditions in STZ-induced mice. | Mihailović et al., 2015 |
| *Laetiporus sulphureus*  (Bull.) Murrill | Crude extracellular polysaccharides (EPS) | Diabetes was induced by streptozotocin (STZ) in rats |  | - Insulin antigenesity was elevated by diabetes islet cell proliferation or regeneration. Reduce plasma glucose and lipid levels, as well as SOD, CAT, and GSH-Px activities, iNOS activity, and cell degeneration. | Hwang and Yun 2010 |
| *Lentinula edodes*  (Berk.) Pegler  “[*Lentinula novae-zelandiae* (G. Stev.) Pegler]” | Exo-polymer |  |  | - Reduces plasma glucose, cholesterol, and triglycerides by improving plasma insulin levels, repairing pancreatic β-cell damage, increasing insulin production, and decreasing plasma glucose, cholesterol, and triglycerides. | Yang et al. 2002 |
|  | Provitamin D2 (ergosterol), glycogen-like polysaccharides, (1-4)-, (1-6)-D-glucans and exopolysaccharide |  |  | - Exopolysaccharide has been demonstrated to reduce blood glucose while raising blood insulin levels. | Bisen et al., 2010 |
|  | Lyophilized and reconstituted |  | Oral treatment of doses of 100 or 200 mg/kg/d | - Improved liver function and a more stable insulin response | Maschio et al., 2017 |
| *Lentinus strigosus*  Fr. | Exopolysaccharides (EPS) from mycelial culture | Diabetes was induced by streptozotocin (STZ, 50 mg/kg/ body weight) in Sprague-Dawley rats | Oral administration of doses 50, 100, and 150 mg/kg/body weight for 7 days | - The plasma glucose level reduced by 21.1 percent at a dose of 150 mg/kg bw. Microvascular pancreatic islet destruction is reversed, and pancreatic islet regeneration is promoted. | Yamac et al. 2008 |
| *Lentinus tigrinus*  (Bull.) Fr. | Lyophilized hot water extraction |  |  | - In mice, it lowers blood glucose levels. | Dulay et al., 2014 |
| *Lignosus rhinocerotis*  (Cooke) Ryvarden | Polysaccharide | Diabetes was induced by streptozotocin (STZ, 35 mg/kg, i.v.) in Male SD rats | Oral treatment of doses at 0.5, 1.0, and 2.0 g/kg bw, for 8 weeks | - In rats, lowers blood glucose, LPO and increased GSH, CAT and SOD | Nyam et al., 2017 |
| *Ophiocordyceps sinensis*  (Berk.) G.H. Sung, J.M. Sung, Hywel-Jones & Spatafora | Polysaccharide | Diabetes was induced by streptozotocin-Wistar rats | Oral treatment of 1 g/day for 4 weeks | - Reduce FBG and oxidative stress | Wu et al., 2020 |
| *Ophiocordyceps sinensis*  (Berk.) G.H. Sung, J.M. Sung, Hywel-Jones & Spatafora and *Cordyceps militaris* (L.) Fr. | Polysaccharide | Diabetes was induced by streptozotocin (40 mg/kg/body weight) in rats | Oral treatment of 10 mg/kg/ body weight for 4 days | - Decrease blood glucose levels by 60-70 % in STZ-induced diabetic mice. | Zhang et al., 2006 |
| *Ophiocordyceps sinensis*  (Berk.) G.H. Sung, J.M. Sung, Hywel-Jones & Spatafora | Polysaccharide fraction CSP-1 | Diabetes was induced by Alloxan-diabetic mice and streptozotocin (STZ)-diabetic rats | Oral treatment of 200 and 400mg/kg body weight/day for 7 days | - As a consequence of this medication, blood glucose levels drop dramatically. Insulin levels in the blood are raised. Reduces insulin metabolism and/or induces the release of insulin from the body's remaining pancreatic cells. | Li et al., 2006 |
| *Pleurotus ostreatus*  (Jacq.) P. Kumm.  “[*Pleurotus cornucopiae* (Paulet) Quél.,]” |  | Diabetes was induced by STZ in Long-Evans rats | Oral treatment of 5% in feed for 8 weeks | - GLUT 4 expression and p-AMPK levels have also increased. | Asrafuzzaman et al., 2018 |
| *Paecilomyces hepiali* |  | In Sprague-Dawley male rats, diabetes was produced by intraperitoneal injection of HFD + STZ (25 mg/kg) + nicotinamide (180 mg/kg). | Oral treatment of 0.08, 0.4, and 2.0 g/kg/p.o. for 4 weeks | - Reduced MDA, IL-2, IL-6, IL-10, and TNF-α; increased SOD, GSH-Px; decreased blood glucose, TC, LDL-C, insulin, PK, glycogen | Wang et al., 2016 |
| *Phallus impudicus*  L. | Mycelium polysaccharide (PIP) | Full-thickness skin flap excision model in Wistar rats | Ointment containing 10% Phallus impudicus polysaccharides | - Epithelialization, granulation tissue contraction, and growth have all improved. | Vyacheslav et al., 2019 |
| *Phellinus badius*  (Cooke) G. Cunn. | Crude exopolysaccharides | Diabetes have been induced by streptozotocin (STZ, 50 mg/kg)-induction in diabetic rats | Oral administration of doses at 50,100,200 mg/kg for two weeks | - The bioactivity assays were carried out using EPS derived from a submerged *P. badius* mycelial culture. When diabetic rats were given different doses of EPS, the 400 mg/kg dosage showed significant hypoglycemic effects, including a drop in blood glucose (37.2%) and an increase in body weight (47.4%), compared with control group. - Triglycerides (23.4%), cholesterol (23.4%), and aspartate aminotransferase (33.8%) and alanine aminotransferase (31.9%) activities all decreased, as did triglycerides (23.4%), cholesterol (23.4%), and aspartate aminotransferase (33.8%) and alanine aminotransferase (31.9%) activities (31.9%). Crude EPS delayed lens opacification and reduced the likelihood of cataract development in sugar-treated goat eye lenses. | Sonawane et al., 2020 |
| *Phellinus baumii*  Pilát | Exopolysaccharides (heteropolysaccharides and proteoglycans) | Streptozotocin (STZ) was used to cause diabetes in male Sprague-Dawley rats. | Oral administration of 50 mg/kg body weight | - After two weeks of oral treatment, STZ-induced diabetic rats had lower AST, ALT, and serum tri-glyceride levels. Blood glucose levels were lowered by 32% in a diabetic mouse model. | Hwang et al., 2005 |
|  | α-linked glycosyl residues | Diabetes was induced by the alloxan-induced diabetic mice | Oral treatment of 100 mg/kg body weight/day | - It decreases blood glucose levels and inhibits H_2_O_2_-induced apoptosis in diabetic mice. | Zhao et al., 2014 |
|  | Hispidin | RINm5F cells |  | - In RINm5F cells treated with H_2_O_2_, cytoprotection against oxidative damage improves β-cell condition and insulin secretion. | Jang et al., 2010 |
|  | Hot aqueous extraction | streptozotocin (STZ)-induced diabetic rats |  | - Nuclear division, ductal stem cell differentiation, and islet cell regeneration all increased in this study. Langerhans islets have become more numerous. | Yamaç et al., 2016 |
|  | Polysaccharides |  |  | - It inhibits the development of autoimmune diabetes by regulating cytokine expression. | Kim et al. 2010 a,b |
|  | Glc and Ara, PLP | Diabetes was established in male Sprague-Dawley diabetic rats by intraperitoneal induction of HFD and STZ (45 mg/kg once). | Oral administration of 600 mg/kg/day, 8 weeks | - Increased Glucose tolerance, insulin tolerance; decreased serum LPS, CRP, TNF-α, IL-6; increased hepatic expression of TLR4 and JNK intestinal tight junction (occludin, claudin-1, ZO1); reverse disruption of intestinal barrier and ameliorate systemic inflammation via enhancing abundance of SCFA-produce bacteria and SCFAs content, thereby helping to reverse insulin resistance | Liu et al., 2020 |
|  | Polysaccharide enriched powder | Diabetes was established in mice with high-fat diet (HFD) and streptozotocin (STZ) induction | Oral treatment of 300 and 600 mg/kg/body weight | - Fasting blood glucose levels were considerably lowered in diabetic rats given dosages of 300 and 600 mg/kg body weight for eight weeks. | Zhao et al., 2018 |
| *Phellinus merrillii*  (Murrill) Ryvarden | Ethanol extracts | α-glucosidase inhibitor |  | - Inhibitory actions against α-glucosidase and aldose reductase | Huang et al. 2011 |
| *Phellinus ribis*  (Schumach.) Quél. | Polychlorinated compounds |  | 100 µM | - Enhancing PPAR-γ agonistic activity has therapeutic implications. | Lee et al. 2008 a |
| *Pleurotus cystidiosus*  O.K. Mill. | [→6)-α-D- Glcp-(1→]n. | Male Kunming mice were given injections of tetraoxypyrimidine (90 mg/kg) to produce hyperglycemia. | 1 mg/kg | - In diabetic mice, polysaccharide administration decreases blood glucose levels. | Wang et al., 2011 |
| *Pleurotus albidus*  (Berk.) Pegler | 70 % ethanol | mitochondrial dysfunction induced by hyperglycaemia in EA.hy926 endothelial cells | 0.1 µg/mL, 0.5 µg/mL, and 1.0 µg/mL | - Reduce ROS generation and lipid and protein oxidative damage by modulating complex 1 activity through SIRT3 regulation. | Gambato et al., 2018 |
| *Pleurotus citrinopileatus*  Singer | Extracellular polysaccharide | *In vitro* inhibitory activity on α-amylase, α-glucosidase |  | - Pancreatic α -amylase, intestinal- α glucosidase, and ACE enzymes were strongly inhibited. | Chen et al., 2017 |
|  | Methanolic extract | Diabetes was induced by streptozotocin (STZ, 70 mg/kg) in Sprague–Dawley rats | Oral administration of dose 500 and 1000 mg/kg | - Control blood glucose levels, insulin, catalase, and pancreatic insulin production in STZ-induced rats. | Rushita et al., 2013 |
|  | Water soluble polysaccharides | Diabetes was induced by streptozotocin (STZ) |  | - Fasting blood glucose levels in diabetic mice were lower. | Hu et al., 2006 |
| *Pleurotus djamor*  (Rumph. ex Fr.) Boedijn | Acidic, alkalic, and enzymatically extractable mycelium zinc polysaccharides (Ac-MZPS, MZPS, Al-, and En-MZPS) | Kunming mice were given 120 mg/kg STZ intraperitoneally for 5 days. | Doses of 200, 400, and 800 mg/kg were given orally for two weeks. | - Increased levels of SOD, GSH-PX, and CAT in STZ-induced diabetic mice were followed by lower levels of ALT, AST, CRE, BUN, TC, MDA, LDL-C, and HDL-C in the kidney, liver, and blood. When mice were compared to a control group, their blood glucose levels were lower. | Zhang et al., 2015 |
| *Pleurotus eryngii*  (DC.) Quél. | PEP | Diabetes was induced in C57BL/6J KKAy mice fed high-fat diet (HF) | Oral administration of 1% or 5% (LP or HP) for 16 weeks | - A rise in anaerostipes and Clostridium was followed by a decrease in body weight, LDL-C, and TC. | Nakahara et al., 2019 |
|  | Extracellular polysaccharide | Diabetes was induced by induction of strepotozotocin (STZ, 150mg/kg, i.p.) | 1 g/kg for 6 weeks | - Blood glucose, control insulin, TC, TG, FBS, OGTT, LDL-C, HDL-C, and liver glycogen were all lowered after oral polysaccharide treatment. | Chen et al., 2016 |
|  | Extracellular polysaccharide | Inhibition of α-amylase and α-glucosidase activities *in vitro*. *In vivo*: diabetic nephropathy (DN) in Kunming strain mice produced by streptozotocin (100 mg/kg). | 600 and 300 mg/kg for 16 days | - The activity of α-amylase and α-glucosidase was higher than that of EPS2. - Polysaccharide administration decreases the activities of -amylase and glucosidase in mice. Blood glucose levels are reduced by EPS 1 and EPS 2. Reduce the blood's high levels of CRE, BUN, ALB, and UA. Also lowered were LDL-C, TGs, TC, and VLDL-C levels, while antioxidant status improved. | Zhang et al., 2018 |
|  | Aqueous extract | Diabetes was induced by induction of strepotozotocin (STZ) |  | - Diabetic mice had considerably decreased blood glucose and HbAlc levels after 5 weeks of therapy. | Li et al., 2014 |
|  | Diet rich with mushroom |  |  | - Insulin sensitivity increased, and it has anti-hyperglycemic and anti-hyperlipidemic properties. | Kim et al. 2010a, b |
| *Pleurotus eryngii* var. *tuoliensis* C.J. Mou.,  “[*Pleurotus tuoliensis* (C.J. Mou) M.R. Zhao & Jin X. Zhang]” | mycelium polysaccharides | Hyperglycemic and hyperlipidemic was induced in Kunming strain mice, high-fat emulsion induced | Oral treatment of doses at 100, 200, 400 mg/kg/body weight for 40 days | - Ac-MPS has to have the potential to eliminate hypertriglyceridemia and inhibit oxidative stress besides reducing TC, TG and LDL-C levels in the blood, raising HDL-C levels in the blood, rising GSH-Px, SOD, CAT, as well as T-AOC actions, and reducing MDA and LPO levels in the liver, heart, kidney, and spleen, according to the findings. As per histopathology, Ac-MPS may also help to prevent organ damage. | Gao et al., 2018 |
| *Pleurotus ferulae* | Polysaccharide | Diabetes was induced by induction of strepotozotocin (STZ) in SD rats | Oral treatment of dose at 250 mg/kg/body weight for 42 days | - HbA1c, TGs, and LDL-C levels were reduced | Wang et al., 2014 |
|  | Polysaccharide | Diabetes was induced by induction of streptozotocin (STZ)-induced diabetic rats | Oral treatment of dose at 250mg/kg/body weight | - Increased enzyme activity of AST, ALT, and ALP, meanwhile HDL and decreased enzyme activity of LDL were discovered. | Choi et al., 2016 |
| *Pleurotus floridanus*  Singer | Polysaccharide fraction of Pleurotus florida. | Diabetes was induced by induction of streptozotocin (STZ, 50 mg/kg/body weight/i.p). | Oral treatment of doses at 200- and 400-mg/kg | - PFP dosages of 200 and 400 mg/kg were used in this investigation. Blood glucose, triglycerides, serum cholesterol, urine glucose and ketones, and glycosylated haemoglobin were among the biological indicators measured. - The amounts of MDA and NO were dramatically reduced, whereas SOD, CAT, and GSH were recovered. | Ganeshpurkar et al., 2014 |
|  | α-glucan |  | Oral administration of 100 and 300 mg/kg | - The polysaccharide doses Lowering triglycerides, cholesterol, and free fatty acids reduces inflammation. | Ganeshpurkar et al., 2014 |
| *Pleurotus giganteus*  (Berk.) S.C. Karunarathna & K.D. Hyde | Methanol |  |  | - Improved GLUT 1 and GLUT 4 expression in 3T3 L1 cells increases adipogenic growth and glucose uptake at different polysaccharide dosages. | Paravamsivam et al., 2016 |
| *Pleurotus ostreatus*  (Jacq.) P. Kumm.  “[*Pleurotus cornucopiae* (Paulet) Quél.,]” |  | Diabetes was induced by induction of streptozotocin (STZ, 35 mg/kg, i.v.) in KK-Ay Mice | Oral treatment of doses at 1.0 and 2.0 g/kg bw, for 4 weeks | - Polysaccharide doses lower blood glucose while promoting AMPK, GLUT-4, Akt, and PKC. | Xiong et al., 2018 |
|  |  | Diabetes was induced by induction of Alloxan (120 mg/kg), Rabbits | Oral treatment of doses at 100, 200, and 300 mg/kg for 4 weeks | - Blood glucose, ALT, ALP, AST, GT, bilirubin, urea, CRE, BUN, Na, and K levels are all reduced by polysaccharide dosages. | Ikewuchi et al., 2017 |
|  | Total polysaccharides extract | Diabetes was induced by induction of high-fat diet and streptozotocin (STZ) in rats | Oral treatment of doses at 100, 200, and 400 mg/kg/body weight | - GSK3 phosphorylation and GLUT4 translocation both increase after dosage administration. Endogenous antioxidants (SOD, CAT, and GSH) activity was increased, and MDA was reduced. Hyperlipidemia and hyperglycemia were decreased. | Zhang et al., 2016 |
|  | Ethanol | Diabetes was induced by induction of Alloxan (150mg/kg/body weight, i.p.) induced in Inbred BALB/C | Oral administration of dose at 250 mg/kg. i.p. | - Diabetic mice showed decreased blood sugar, triglycerides, LDL and VLDL cholesterol, serum cholesterol, SGPT and SGOT, serum creatinine, as well as greater HDL cholesterol following Fifteen days of treatment. | Ravi et al., 2012 |
| *Pleurotus ostreatus*  *(*Jacq.) P. Kumm. “[*Pleurotus cornucopiae* (Paulet) Quél.,]” and *Pleurotus cystidiosus*  O.K. Mill. | Freeze-dried and powdered | Diabetes was induced by induction of streptozotocin (STZ) in rats | Oral treatment of various doses 250, 500, 750, 1000, and 1250 mg/kg body weight, | - Freeze-dried and powdered polysaccharides showed potential hypoglycaemic action after treatment, with a dose-dependent effect. | Jayasuriya et al., 2012 |
| *Pleurotus ostreatus*  (Jacq.) P. Kumm*,* “[*Pleurotus cornucopiae* (Paulet) Quél.,]”, *Calocybe indica*  Purkayastha & A. Chandra and *Volvariella volvacea*  (Bull.) Singer | Polysaccharides | Diabetes was induced by induction of streptozotocin (STZ, 150 mg/kg, i.p.), in Male ICR mice | Oral treatment of various doses 200 and 400 mg/kg/body weight for 6 weeks | - Glucose absorption, glucose adsorption capacity, and blood glucose levels were measured in α-amylase inhibition experiment. | Singh et al., 2017 |
| *Pleurotus pulmonarius*  (Fr.) Quél.  “[*Pleurotus ostreatus* (Jacq.) P. Kumm.,]” | Glyceraldehyde-3-phosphate dehydrogenase, profilin-1, and guanide | Diabetes was induced by induction of streptozotocin (STZ) in Wistar rats |  | - MALDI-TOF/TOF MS revealed the presence of four distinct proteins that could be involved in blood glucose regulation via a variety of methods. Wistar rats given STZ-Nicotinamide - High inhibitory action against -glucosidase and -amylase - Guanide has been shown to cause exocytosis of insulin-containing granules and reduce fasting plasma glucose levels. | Wahab et al., 2014 |
|  |  |  | Oral treatment of doses 200 and 400 mg/kg/bw for 4 weeks | - The antioxidant status as well as the lipid profile were both enhanced (lower TG, TC, LDL-C, and increased HDL-C). The damage to the kidneys, liver, and pancreas reduced. | Balaji et al., 2020 |
|  | Aqueous extract of the mushroom | Diabetes was induced by induction of Alloxan induced diabetic mice | Oral treatment of dose 500 mg/kg, p.o. for 28 days | - Diabetic mice given alloxan exhibited decreased blood glucose levels and better glucose tolerance. | Badole et al. 2006 |
| *Pleurotus tuber-regium*  (Fr.) Singer | HP, MP and LP; polysaccharides | Diabetes was induced by the intraperitoneal injection of streptozotocin 10 mg/kg + 30 mg/kg nicotinamide every other day for 6 weeks | Oral treatment of dose 20 mg/kg for 8 weeks | - Increase PPAR-γ mRNA and protein levels to prevent obesity, hyperglycemia, hyperlipidemia, and diabetes. | Huang etal., 2014 |
|  | Aqueous extract |  |  | - It decreases blood glucose, triglycerides, and improves lipid profile in diabetic rabbits. | Onuekwuzu et al., 2019 |
| *Pleurotus sajor-caju*  (Fr.) Singer | β-glucan rich polysaccharide polysaccharide | lipogenesis, lipolysis and oxidative stress in 3T3-L1 cells |  | - Reduce the expression of IL-6, TNF-α, SAA2, CRP, and MCP 1 by upregulating GLUT 4 and adiponectin. In 3T3-L1 cells, activate the AMPK unit, which controls lipid and glucose metabolism. Adiponectin, leptin, hormone-sensitive triglyceride lipase, ATL, and GLUT4 are all upregulated. | Kanagasabapathy et al., 2012 |
|  | Aqueous extraction | Diabetes was induced by the intraperitoneal injection of Streptozotocin (STZ) (40 mg/kg BW) in Male Sprague-Dawley rats | Oral treatment of 750  mg/kg for 21 days | - Mice with larger body weight and lower blood glucose levels - The 750 mg/kg dosage resulted in a maximal BG decline of 41.3 percent in normal rats and 36.5 % in diabetic rats 3 hours after glucose delivery in an OGTT investigation. | Ng et al., 2015 |
| *Pleurotus djamor*  (Rumph. ex Fr.) Boedijn | Ac-MZPS, AlMZPS and EnMZPS; acidic-, alkalicand enzymaticextractable mycelium zinc polysaccharides | Diabetes was induced by the intraperitoneal injection of Streptozotocin (STZ, 120 mg/kg) in Kunming mice for 5 days | Oral treatment of 200, 400 and 800 mg/kg for 2 weeks | - SOD, GSH-Px, and CAT levels up; MDA, CRE, ALT, BUN, AST, HDL-C, TC, LDL-C, and levels decreased; liver and kidney damage alleviated | Zhang et al., 2015 |
| *Poria cocos* F.A. Wolf | Chloroform fractions, Dehydrotrametenolic acid, Dehydrotumulosic acid, Pachymic acid | Diabetes was induced by an intraperitoneal injection of STZ (200 mg/kg) in Male C57BL/KsJ-db/db mice | Oral treatment of 50 mg/kg body | - Lower postprandial blood glucose in diabetic mice by ignoring PPAR-γ. An insulin sensitizer's activity. STZ-induced diabetes in mice | Li et al., 2010 |
| *Sparassis crispa*  (Wulfen) Fr. | β-glucan component | Diabetes was induced by streptozotocin (STZ)-induced diabetic rats | Oral treatment of 1,000 mg/kg/ body weight for 4 weeks | - It's a potent wound-healing stimulator in diabetics. Enhanced macrophage and fibroblast migration, as well as increased type I collagen synthesis. | Kwon et al. 2008 |
| *Stropharia rugosoannulata*  Farl. ex Murrill | EPS | Diabetes was induced by streptozotocin (STZ, 50 mg/kg/ body weight) induced in male Kunming mice | Oral treatment of 100 and  200 mg/kg | - Reduced plasma glucose, total cholesterol, and triacylglycerol levels; reduced aspartate aminotransferase activity; PPAR-γ mediated lipid metabolism | He et al. 2012; Ma et al., 2013 |
| *Suillellus luridus*  (Schaeff.) Murrill | polysaccharides | Diabetes was induced by streptozotocin (STZ)-Male mice | Oral treatment of 150 mg/kg/body weight for 30 days | - The polysaccharides have anti-diabetic, anti-hyperlipidemic, and antioxidant properties (improved SOD, GPx, MDA and CAT enzymes). | Zhang et al., 2018 |
| *Trametes pubescens*  (Schumach.) Pilát | Hot water and methanolic extract | α-amylase and α-glucosidase inhibitory activities | 2-40 μg/mL | - On the RAW 264.7 cell line, inhibition of α-amylase and α-glucosidase, as well as inhibition of inflammation. | Im et al., 2016 |
| *Trametes versicolor*  (L.) Lloyd |  | Insulin-resistant HepG2 cells | Oral treatment of 50, 100, and 1000 μg/ml | - Upregulation of glucose kinase, membrane GLUT1, and glucose-6-phosphatase, and downregulation of glycogen synthase kinase 3. | Teng et al., 2018 |
|  | TVP LH-1ePSP; monosaccharide composition analysis | Diabetes was induced by Injection of streptozotocin (STZ, 65 mg/kg) in male Wistar rats for 5 days | Oral treatment of 100 mg/kg for 28 days | - The maintenance of bone characteristics is aided by improved hyperglycemic control. | Chen et al., 2015 |
| *Tremella aurantia*  Schwein. | Acidic heteropolysaccharide and various carbohydrates, including glucose, are found in the fruiting bodies. | Diabetes was induced by streptozotocin and  nicotinamide and on days 0, 7, and 14 in Male Wistar rats | Oral treatment of 1 g/kg/day, day 1 to day 14) | - Increased blood glucose, total cholesterol, triglyceride, and lipoperoxide levels were also reduced. - Improves insulin sensitivity, potentially via modulating PPAR- γ, and lower plasma glucose levels by 52% compared to control mice. | Lo et al., 2006 |
|  | Acidic polysaccharide | Diabetes was induced by Male 4-week-old KK-Ay  -TA (type 2  diabetes) | TAP-H - degradation product of TAP 1.5 g/l) | - Serum glucose, total cholesterol, and triglyceride levels were all reduced. The level of plasma lipoperoxide has significantly decreased. | Kiho et al. 2001 |
| *Tremella aurantialba*  Bandoni & M. Zang | TMP; crude polysaccharide | Diabetes was induced by Intraperitoneal injection of 180 mg/kg alloxan in Wistar rats for 5 days | Oral treatment of 100 mg/kg for 28 days | - Regulation of lipid metabolism and mitigation of oxidative stress | Zhang et al., 2009 |
| *Tremella fuciformis*  Berk. | Exopolysaccharides | Diabetes was induced by male C57BL/6J ob/ob  mice | Oral treatment of 200 mg/kg body weight for 52 days | - Fasting blood glucose levels were lower, glucose tolerance was better, and systemic insulin sensitivity was better. The peroxisome profilerator-activated receptor (PPAR-γ) regulates lipid metabolism. - Exopolysaccharides identified in the mushroom increased insulin sensitivity, whereas gluronoxylomannan was connected to hypoglycemic activity. | Cho et al. 2007 |
| *Tremella mesenterica*  Retz. | Fruiting body extract |  |  | - Type 1 diabetic rats and pre-diabetic impaired glucose tolerant rats with streptozocin showed reduced fasting blood glucose levels. | Lo et al. 2006 |
|  | Acidic polysaccharide Glucuronoxylo mannan | Diabetes was induced by streptozotocin (STZ) in KK-Ay mice | Oral treatment of 450 or 150 mg kg (-1) | - Glucuronoxylomannan Only streptozocin or both STZ and nicotinamide reduced high blood glucose levels in diabetic rats. | Hong et al., 2007 |
| *Tuber melanosporum*  Vittad. |  | Diabetes was induced by streptozotocin (STZ) in Wistar Albino rats | Oral treatment of 400 mg/kg for 45 days | - The levels of HbA1c and oxidative stress were lowered, whereas antioxidant status was improved. | Zhang et al., 2018 |
| *Wolfiporia cocos*  (F.A. Wolf) Ryvarden & Gilb*.* | Dehydrotumulosic acid, polyporenic acid C, pachymic acid, dehydrotrametenolic acid, and dehydroeburicoic acid are some of the acids found in dehydrotumulosic acid. |  |  | - It decreases hyperglycemia and works as an insulin sensitizer in glucose tolerance tests. | Fu et al., 2018 |
| *Wolfiporia extensa*  (Peck) Ginns | Triterpenoids like pachymic acid and polysaccharides. |  |  | - Insulin sensitivity is improved, and postprandial blood glucose levels are decreased. | Li et al., 2016 |

Alanine transferase (ALT); Albumin (ALB); Alkaline phosphatase (ALP); AMP-activated protein kinase (AMPK); Aspartic aminotransferase (AST); Blood glucose (GLU); Catalase (CAT); Very low density lipoprotein (VLDL); Creatinine (CRE); High density lipoprotein (HDL); Glucose transporter 4 (GLUT4); Glutathione (GSH); Glutathione peroxidase (GSH-Px); High-density lipoprotein cholesterol (HDL-C); Lipid peroxidation (LPO); Low-density lipoprotein cholesterol (LDL-C); Malondialdehyde (MDA); Protein kinase C (PKC)*;* Reactive oxygen species (ROS); Low density lipoprotein (LDL); Superoxide dismutase (SOD); Total antioxidative capacity (T-AOC); Total cholesterol (TC); Triglycerides (TGs); Blood urea nitrogen (BUN); uric acid (UA).

**References**

Aoki, H., Hanayama, M., Mori, K., and Sato, R. (2018). Grifola frondosa (Maitake) extract activates PPARδ and improves glucose intolerance in high-fat diet-induced obese mice. *Biosci. Biotechnol. Biochem*. 82, 1550–1559.

Asrafuzzaman, M., Rahman, M. M., Mandal, M., Marjuque, M., Bhowmik, A., Rokeya, B., et al. (2018). Oyster mushroom functions as an anti-hyperglycaemic through phosphorylation of AMPK and increased expression of GLUT4 in type 2 diabetic model rats. *J. Taibah Univ*. Med. Sci. 13, 465–471.

Badole, S. L., Patel, N. M., Thakurdesai, P. A., and Bodhankar, S. L. (2008). Interaction of Aqueous Extract of Pleurotus pulmonarius (Fr.) Quel-Champ. with Glyburide in Alloxan Induced Diabetic Mice. *Evid. Based Complement. Alternat. Med*. 5, 159–164. doi:10.1093/ecam/nem010.

Balaji, P., Madhanraj, R., Rameshkumar, K., Veeramanikandan, V., Eyini, M., Arun, A., ... & Kim, H. J. (2020). Evaluation of antidiabetic activity of Pleurotus pulmonarius against streptozotocin-nicotinamide induced diabetic wistar albino rats. *Saudi J. Biol. Sci*. 27(3), 913-924.

Balakrishnan, P., and Loganayagi, C. T. (2018). Antihyperglycemic activity of Agaricus bisporus mushroom extracts on alloxan induced diabetic rats. *Int. J. Pharma. Res. Health Sci.* 6, 2475–79.

Bisen, P. S., Baghel, R. K., Sanodiya, B. S., Thakur, G. S., and Prasad, G. (2010). Lentinus edodes: a macrofungus with pharmacological activities. *Curr. Med. Chem.* 17, 2419–2430.

Chaiyasut, C., and Sivamaruthi, B. S. (2017). Anti-hyperglycemic property of Hericium erinaceus–A mini review. *Asian Pac. J. Trop. Biomed*. 7, 1036–1040.

Chen, C.-H., Kang, L., Lo, H.-C., Hsu, T.-H., Lin, F.-Y., Lin, Y.-S., et al. (2015). Polysaccharides of Trametes versicolor improve bone properties in diabetic rats. J. Agric. *Food Chem.* 63, 9232–9238.

Chen, F., Grimm, A., Eilertsen, L., Martín, C., Arshadi, M., & Xiong, S. (2021). Integrated production of edible mushroom (Auricularia auricular-judae), fermentable sugar and solid biofuel. Renewable Energy, 170, 172-180.

Chen, H., Tian, T., Miao, H., and Zhao, Y.-Y. (2016a). Traditional uses, fermentation, phytochemistry and pharmacology of Phellinus linteus: A review. *Fitoterapia* 113, 6–26.

Chen, J., Gao, J., Yu, Y., and Yang, S. (2019). A hyaluronan-based polysaccharide peptide generated by a genetically modified Streptococcus zooepidemicus. *Carbohydr. Res*. 478, 25–32.

Chen, L., Zhang, Y., Sha, O., Xu, W., and Wang, S. (2016b). Hypolipidaemic and hypoglycaemic activities of polysaccharide from Pleurotus eryngii in Kunming mice. *Int. J. Biol. Macromol.* 93, 1206–1209.

Chen, P.-H., Weng, Y.-M., Lin, S.-M., Yu, Z.-R., and Wang, B.-J. (2017). Molecular weight affected antioxidant, hypoglycemic and hypotensive activities of cold water extract from Pleurotus citrinopileatus. *J. Food Sci*. 82, 2456–2461.

Chen, Y.-H., Lee, C.-H., Hsu, T.-H., and Lo, H.-C. (2015). Submerged-culture mycelia and broth of the maitake medicinal mushroom Grifola frondosa (higher basidiomycetes) alleviate type 2 diabetes-induced alterations in immunocytic function. *Int. J. Med. Mushrooms* 17.

Cho, E. J., Hwang, H. J., Kim, S. W., Oh, J. Y., Baek, Y. M., Choi, J. W., et al. (2007). Hypoglycemic effects of exopolysaccharides produced by mycelial cultures of two different mushrooms Tremella fuciformis and Phellinus baumii in ob/ob mice. *Appl. Microbiol. Biotechnol.* 75, 1257–1265.

Choi, D., Piao, Y., Yu, S.-J., Lee, Y.-W., Lim, D.-H., Chang, Y.-C., et al. (2016). Antihyperglycemic and antioxidant activities of polysaccharide produced from Pleurotus ferulae in streptozotocin-induced diabetic rats. *Korean J. Chem. Eng*. 33, 1872–1882.

Cui, B., Han, L., Qu, J., and Lv, Y. (2009). Hypoglycemic activity of Grifola frondosa rich in vanadium. *Biol. Trace Elem. Res.* 131, 186–191.

Ding, Z., Lu, Y., Lu, Z., Lv, F., Wang, Y., Bie, X., et al. (2010). Hypoglycaemic effect of comatin, an antidiabetic substance separated from Coprinus comatus broth, on alloxan-induced-diabetic rats. *Food Chem.* 121, 39–43.

Dong, Y., Jing, T., Meng, Q., Liu, C., Hu, S., Ma, Y., et al. (2014). Studies on the antidiabetic activities of Cordyceps militaris extract in diet-streptozotocin-induced diabetic Sprague-Dawley rats. *BioMed Res. Int*. 2014.

Dulay, R. M. R., Arenas, M. C., Kalaw, S. P., Reyes, R. G., and Cabrera, E. C. (2014). Proximate composition and functionality of the culinary-medicinal tiger sawgill mushroom, Lentinus tigrinus (higher Basidiomycetes), from the Philippines. *Int. J. Med. Mushrooms* 16.

Ekowati, N., Yuniati, N. I., Hernayanti, H., and Ratnaningtyas, N. I. (2018). Antidiabetic potentials of button mushroom (Agaricus bisporus) on alloxan-induced diabetic rats. *Biosaintifika J. Biol. Biol. Educ*. 10, 655–662.

Fatmawati, S., Shimizu, K., and Kondo, R. (2011). Ganoderol B: a potent α-glucosidase inhibitor isolated from the fruiting body of Ganoderma lucidum. *Phytomedicine* 18, 1053–1055.

Fu, M., Wang, L., Wang, X., Deng, B., Hu, X., and Zou, J. (2018). Determination of the five main terpenoids in different tissues of Wolfiporia cocos. *Molecules* 23, 1839.

Gambato, G., Pavão, E. M., Chilanti, G., Fontana, R. C., Salvador, M., and Camassola, M. (2018). Pleurotus albidus modulates mitochondrial metabolism disrupted by hyperglycaemia in EA. hy926 endothelial cells. *BioMed Res. Int.* 2018.

Ganeshpurkar, A., Kohli, S., and Rai, G. (2014). Antidiabetic potential of polysaccharides from the white oyster culinary-medicinal mushroom Pleurotus florida (higher Basidiomycetes). *Int. J. Med. Mushrooms*,16(3), 207-217.

Gao, Z., Kong, D., Cai, W., Zhang, J., and Jia, L. (2021). Characterization and anti-diabetic nephropathic ability of mycelium polysaccharides from Coprinus comatus. *Carbohydr. Polym.* 251, 117081.

Gao, Z., Lai, Q., Yang, Q., Xu, N., Liu, W., Zhao, F., et al. (2018). The characteristic, antioxidative and multiple organ protective of acidic-extractable mycelium polysaccharides by Pleurotus eryngii var. tuoliensis on high-fat emulsion induced-hypertriglyceridemic mice. *Sci. Rep.* 8, 1–12.

Gargiulo, G., 2018. Next-Generation in vivo Modeling of Human Cancers. Frontiers in Oncology, 8. https://www.ncbi.nlm.nih.gov/pmc/articles/PMC6192385/

Geng, Y., Lu, Z.-M., Huang, W., Xu, H.-Y., Shi, J.-S., and Xu, Z.-H. (2013). Bioassay-guided isolation of DPP-4 inhibitory fractions from extracts of submerged cultured of Inonotus obliquus. *Molecules* 18, 1150–1161.

Guo, W.-L., Deng, J.-C., Pan, Y.-Y., Xu, J.-X., Hong, J.-L., Shi, F.-F., et al. (2020). Hypoglycemic and hypolipidemic activities of Grifola frondosa polysaccharides and their relationships with the modulation of intestinal microflora in diabetic mice induced by high-fat diet and streptozotocin. *Int. J. Biol. Macromol*. 153, 1231–1240.

Harada, E., Morizono, T., and Saito, M. (2017). Blood glucose–reducing and fat-reducing effects of a novel medicinal mushroom, Grifola gargal (Agaricomycetes). *Int. J. Med. Mushrooms*.19(12),1071-1081

He, C.-Y., Li, W.-D., Guo, S.-X., Lin, S.-Q., and Lin, Z.-B. (2006). Effect of polysaccharides from Ganoderma lucidum on streptozotocin-induced diabetic nephropathy in mice. *J. Asian Nat. Prod. Res.* 8, 705–711.

He, P., Geng, L., Wang, J., and Xu, C. (2012). Production, purfication, molecular characterization and bioactivities of exopolysaccharides produced by the wine cap culinary-medicinal mushroom, Stropharia rugosoannulata 2#(higher basidiomycetes). *Int. J. Med. Mushrooms*, 14(4), 365-76.

Højelse, F., 2000. Preclinical Safety Assessment: In vitro - in vivo Testing. Pharmacol. Toxicol, 86, pp.6-7. https://pubmed.ncbi.nlm.nih.gov/10905745/

Hong, L., Xun, M., and Wutong, W. (2007). Anti-diabetic effect of an α-glucan from fruit body of maitake (Grifola frondosa) on KK-Ay mice. J. Pharm. Pharmacol. 59, 575–582.

Hu, S.-H., Wang, J.-C., Lien, J.-L., Liaw, E.-T., and Lee, M.-Y. (2006). Antihyperglycemic effect of polysaccharide from fermented broth of Pleurotus citrinopileatus. *Appl. Microbiol. Biotechnol.* 70, 107–113.

Huang, G.-J., Hsieh, W.-T., Chang, H.-Y., Huang, S.-S., Lin, Y.-C., and Kuo, Y.-H. (2011). α-Glucosidase and aldose reductase inhibitory activities from the fruiting body of Phellinus merrillii. *J. Agric. Food Chem.* 59, 5702–5706.

Huang, H.-Y., Korivi, M., Yang, H.-T., Huang, C.-C., Chaing, Y.-Y., and Tsai, Y.-C. (2014). Effect of Pleurotus tuber-regium polysaccharides supplementation on the progression of diabetes complications in obese-diabetic rats. *Chin. J. Physiol*. 57, 198–208.

Hwang, H. S., & Yun, J. W. (2010). Hypoglycemic effect of polysaccharides produced by submerged mycelial culture of Laetiporus sulphureus on streptozotocininduced diabetic rats. Biotechnol. Biop. Eng. 15(1), 173-181.

Hwang, H.-J., Kim, S.-W., Lim, J.-M., Joo, J.-H., Kim, H.-O., Kim, H.-M., et al. (2005). Hypoglycemic effect of crude exopolysaccharides produced by a medicinal mushroom Phellinus baumii in streptozotocin-induced diabetic rats. *Life Sci.* 76, 3069–3080.

Ikewuchi, C. C., Ikewuchi, J. C., and Ifeanacho, M. O. (2017). Restoration of plasma markers of liver and kidney functions/integrity in alloxan-induced diabetic rabbits by aqueous extract of Pleurotus tuberregium sclerotia. *Biomed. Pharmacother.* 95, 1809–1814.

Im, K. H., Nguyen, T. K., Choi, J., and Lee, T. S. (2016). In vitro antioxidant, anti-diabetes, anti-dementia, and inflammation inhibitory effect of Trametes pubescens fruiting body extracts. *Molecules* 21, 639.

Jang, J. S., Lee, J. S., Lee, J. H., Kwon, D. S., Lee, K. E., Lee, S. Y., et al. (2010). Hispidin produced from Phellinus linteus protects pancreatic β-cells from damage by hydrogen peroxide. *Arch. Pharm. Res.* 33, 853–861.

Jayasuriya, W., Suresh, T. S., Abeytunga, D. T. U., Fernando, G. H., and Wanigatunga, C. A. (2012). Oral hypoglycemic activity of culinary-medicinal mushrooms Pleurotus ostreatus and P. cystidiosus (higher basidiomycetes) in normal and alloxan-induced diabetic Wistar rats. *Int. J. Med. Mushrooms*, 14(4), 347-355.

Jeong, S. C., Jeong, Y. T., Yang, B. K., Islam, R., Koyyalamudi, S. R., Pang, G., et al. (2010). White button mushroom (Agaricus bisporus) lowers blood glucose and cholesterol levels in diabetic and hypercholesterolemic rats. *Nutr. Res.* 30, 49–56.

Joo, J. I., Kim, D. H., and Yun, J. W. (2010). Extract of Chaga mushroom (Inonotus obliquus) stimulates 3t3-l1 adipocyte differentiation. *Phytother. Res*. 24, 1592–1599.

Kanagasabapathy, G., Kuppusamy, U. R., Abd Malek, S. N., Abdulla, M. A., Chua, K.-H., and Sabaratnam, V. (2012). Glucan-rich polysaccharides from Pleurotus sajor-caju (Fr.) Singer prevents glucose intolerance, insulin resistance and inflammation in C57BL/6J mice fed a high-fat diet. *BMC Complement. Altern. Med*. 12, 1–9.

Kiho, T., Kochi, M., Usui, S., Hirano, K., AizawA, K., and Inakuma, T. (2001). Antidiabetic effect of an acidic polysaccharide (TAP) from Tremella aurantia and its degradation product (TAP-H). *Biol. Pharm. Bull.* 24, 1400–1403.

Kim, H. M., Kang, J. S., Kim, J. Y., Park, S.-K., Kim, H. S., Lee, Y. J., et al. (2010a). Evaluation of antidiabetic activity of polysaccharide isolated from Phellinus linteus in non-obese diabetic mouse. *Int. Immunopharmacol*. 10, 72–78.

Kim, J.-I., Kang, M.-J., Im, J., Seo, Y.-J., Lee, Y.-M., Song, J.-H., et al. (2010b). Effect of king oyster mushroom (Pleurotus eryngii) on insulin resistance and dyslipidemia in db/db mice. *Food Sci. Biotechnol*. 19, 239–242.

Kim, S.-K., Hong, U.-P., Kim, J.-S., Kim, C.-H., Lee, K.-W., Choi, S.-E., et al. (2007). Antidiabetic effect of Auricularia auricula mycelia in streptozotocin-induced diabetic rats. *Nat. Prod. Sci*. 13, 390–393.

Kim, Y. O., Han, S. B., Lee, H. W., Ahn, H. J., Yoon, Y. D., Jung, J. K., et al. (2005). Immuno-stimulating effect of the endo-polysaccharide produced by submerged culture of Inonotus obliquus. *Life Sci*. 77, 2438–2456.

Kobayashi, M., Kawashima, H., Takemori, K., Ito, H., Murai, A., Masuda, S., et al. (2012). Ternatin, a cyclic peptide isolated from mushroom, and its derivative suppress hyperglycemia and hepatic fatty acid synthesis in spontaneously diabetic KK-Ay mice. *Biochem. Biophys. Res. Commun.* 427, 299–304.

Kou, L., Du, M., Liu, P., Zhang, B., Zhang, Y., Yang, P., et al. (2019). Anti-diabetic and anti-nephritic activities of Grifola frondosa mycelium polysaccharides in diet-streptozotocin-induced diabetic rats via modulation on oxidative stress. *Appl. Biochem. Biotechnol*. 187, 310–322.

Kwon, A.-H., Qiu, Z., Hashimoto, M., Yamamoto, K., and Kimura, T. (2009). Effects of medicinal mushroom (Sparassis crispa) on wound healing in streptozotocin-induced diabetic rats. *Am. J. Surg*. 197, 503–509.

Lee, I.-K., Lee, J.-H., and Yun, B.-S. (2008a). Polychlorinated compounds with PPAR-γ agonistic effect from the medicinal fungus Phellinus ribis. *Bioorg. Med. Chem. Lett*. 18, 4566–4568.

Lei, H., Guo, S., Han, J., Wang, Q., Zhang, X., and Wu, W. (2012). Hypoglycemic and hypolipidemic activities of MT-α-glucan and its effect on immune function of diabetic mice. *Carbohydr. Polym*. 89, 245–250.

Li, J.-P., Lei, Y., and Zhan, H. (2014b). The effects of the king oyster mushroom Pleurotus eryngii (higher Basidiomycetes) on glycemic control in alloxan-induced diabetic mice. *Int. J. Med. Mushrooms,* 16(3), 219-225.

Li, L., Guo, W.-L., Zhang, W., Xu, J.-X., Qian, M., Bai, W.-D., et al. (2019). Grifola frondosa polysaccharides ameliorate lipid metabolic disorders and gut microbiota dysbiosis in high-fat diet fed rats. *Food Funct*. 10, 2560–2572.

Li, S. P., Zhang, G. H., Zeng, Q., Huang, Z. G., Wang, Y. T., Dong, T. T. X., et al. (2006). Hypoglycemic activity of polysaccharide, with antioxidation, isolated from cultured Cordyceps mycelia. *Phytomedicine* 13, 428–433.

Li, T. H., Hou, C. C., Chang, C. L. T., & Yang, W. C. (2010). Anti-hyperglycemic properties of crude extract and triterpenes from Poria cocos. Evidence-Based Complementary and Alternative Medicine, 2011. vol. 2011, Article ID 128402, 8 pages, 2011. https://doi.org/10.1155/2011/128402

Li, Y., Zhang, J., Li, T., Liu, H., and Wang, Y. (2016). A comprehensive and comparative study of Wolfiporia extensa cultivation regions by Fourier transform infrared spectroscopy and ultra-fast liquid chromatography. *PLoS One* 11, e0168998.

Li, Z. (2011). Protective effects of Ganoderma lucidum polyccharide on myocardial fibrosis. *Chin. Hosp. Pharm. J.* 31 (20), 1706–1710.

Liao, X., Yang, L., Chen, M., Yu, J., Zhang, S., and Ju, Y. (2015). The hypoglycemic effect of a polysaccharide (GLP) from Gracilaria lemaneiformis and its degradation products in diabetic mice. *Food Funct*. 6, 2542–2549.

Lin, L., Cui, F., Zhang, J., Gao, X., Zhou, M., Xu, N., et al. (2016). Antioxidative and renoprotective effects of residue polysaccharides from Flammulina velutipes. *Carbohydr. Polym.* 146, 388–395

Liu, M., Song, X., Zhang, J., Zhang, C., Gao, Z., Li, S., et al. (2017). Protective effects on liver, kidney and pancreas of enzymatic-and acidic-hydrolysis of polysaccharides by spent mushroom compost (Hypsizigus marmoreus). *Sci. Rep*. 7, 1–12.

Liu, Y., Chen, D., You, Y., Zeng, S., Hu, Y., Duan, X., et al. (2016b). Structural characterization and antidiabetic activity of a glucopyranose-rich heteropolysaccharide from Catathelasma ventricosum. *Carbohydr. Polym*. 149, 399–407.

Liu, Y., Sun, J., Rao, S., Su, Y., and Yang, Y. (2013c). Antihyperglycemic, antihyperlipidemic and antioxidant activities of polysaccharides from Catathelasma ventricosum in streptozotocin-induced diabetic mice. *Food Chem. Toxicol*. 57, 39–45.

Liu, Y., Wang, C., Li, J., Li, T., Zhang, Y., Liang, Y., et al. (2020). Phellinus linteus polysaccharide extract improves insulin resistance by regulating gut microbiota composition. *FASEB J.* 34, 1065–1078.

Lo, H.-C., Hsu, T.-H., and Chen, C.-Y. (2008). Submerged culture mycelium and broth of Grifola frondosa improve glycemic responses in diabetic rats. *Am. J. Chin. Med*. 36, 265–285.

Lo, H.-C., Tsai, F.-A., Wasser, S. P., Yang, J.-G., and Huang, B.-M. (2006). Effects of ingested fruiting bodies, submerged culture biomass, and acidic polysaccharide glucuronoxylomannan of Tremella mesenterica Retz.: Fr. on glycemic responses in normal and diabetic rats. *Life Sci*. 78, 1957–1966.

Lu, X., Chen, H., Dong, P., Fu, L., and Zhang, X. (2010). Phytochemical characteristics and hypoglycaemic activity of fraction from mushroom Inonotus obliquus. *J. Sci. Food Agric*. 90, 276–280.

Lv, X.-C., Guo, W.-L., Li, L., Yu, X.-D., and Liu, B. (2019). Polysaccharide peptides from Ganoderma lucidum ameliorate lipid metabolic disorders and gut microbiota dysbiosis in high-fat diet-fed rats. *J. Funct. Foods* 57, 48–58.

Ma, Y., Mao, D., Geng, L., Wang, Z., and Xu, C. (2013). Production, fractionation, characterization of extracellular polysaccharide from a newly isolated Trametes gibbosa and its hypoglycemic activity. *Carbohydr. Polym*. 96, 460–465.

Manohar, V., Talpur, N. A., Echard, B. W., Lieberman, S., and Preuss, H. G. (2002). Effects of a water-soluble extract of maitake mushroom on circulating glucose/insulin concentrations in KK mice. *Diabetes Obes. Metab*. 4, 43–48.

Maschio, B. H., Gentil, B. C., Caetano, E. L. A., Rodrigues, L. S., Laurino, L. F., Spim, S. R. V., et al. (2017). Characterization of the effects of the shiitake culinary-medicinal mushroom, lentinus edodes (agaricomycetes), on severe gestational diabetes mellitus in rats. *Int. J. Med. Mushrooms,* 19(11), 991-1000.

Meera, K. S., Sudha, G., Rajathi, K., and Manjusha, G. V. (2011). Antidiabetic effect of aqueous extract of Hypsizygus ulmarius on Streptozotocin-Nicotiinamide induced diabetic rats. *Asian J. Pharm. Biol. Res. AJPBR* 1(2), 151-157.

Meng, G., Zhu, H., Yang, S., Wu, F., Zheng, H., Chen, E., et al. (2011). Attenuating effects of Ganoderma lucidum polysaccharides on myocardial collagen cross-linking relates to advanced glycation end product and antioxidant enzymes in high-fat-diet and streptozotocin-induced diabetic rats. *Carbohydr. Polym*. 84, 180–185.

Mihailović, M., Arambašić Јovanović, J., Uskoković, A., Grdović, N., Dinić, S., Vidović, S., et al. (2015). Protective effects of the mushroom Lactarius deterrimus extract on systemic oxidative stress and pancreatic islets in streptozotocin-induced diabetic rats. *J. Diabetes Res*. 2015, 576726. doi: 10.1155/2015/576726

Nakahara, D., Nan, C., Mori, K., Hanayama, M., Kikuchi, H., Hirai, S., et al. (2020). Effect of mushroom polysaccharides from Pleurotus eryngii on obesity and gut microbiota in mice fed a high-fat diet. *Eur. J. Nutr.* 59, 3231–3244.

Ng, S. H., Mohd Zain, M. S., Zakaria, F., Wan Ishak, W. R., and Wan Ahmad, W. A. N. (2015). Hypoglycemic and antidiabetic effect of Pleurotus sajor-caju aqueous extract in normal and streptozotocin-induced diabetic rats. *BioMed Res. Int*. 2015, 214918. doi: 10.1155/2015/214918.

Nweze, C. C., Rasaq, N. O., and Istifanus, B. I. (2020). Ameliorating effect of Agaricus bisponus and Pleurotus ostreatus mixed diet on Alloxan-induced hyperglycemic rats. *Sci. Afr*. 7, e00209.

Nyam, K. L., Chow, C. F., Tan, C. S., and Ng, S. T. (2017). Antidiabetic properties of the tiger’s milk medicinal mushroom, Lignosus rhinocerotis (Agaricomycetes), in streptozotocin-induced diabetic rats. *Int. J. Med. Mushrooms*, 19(7), 607-617. doi: 10.1615/IntJMedMushrooms.2017021186.

Ogbole, O. O., Nkumah, A. O., Linus, A. U., & Falade, M. O. (2019). Molecular identification, in vivo and in vitro activities of Calvatia gigantea (macro-fungus) as an antidiabetic agent. Mycology, 10(3), 166-173.

Onuekwuzu, I. M., Chidinma, I. C., and Chigozie, I. J. (2019). Anti-diabetic effect of a flavonoid and sitosterol-rich aqueous extract of Pleurotus tuberregium sclerotia in alloxan-induced diabetic rabbits. *Endocr. Metab. Immune Disord.-Drug Targets Former. Curr. Drug Targets-Immune Endocr. Metab. Disord.* 19, 1148–1156.

Pan, D., Zhang, D., Wu, J., Chen, C., Xu, Z., Yang, H., et al. (2013). Antidiabetic, antihyperlipidemic and antioxidant activities of a novel proteoglycan from Ganoderma lucidum fruiting bodies on db/db mice and the possible mechanism. *PloS One* 8, e68332.

Paravamsivam, P., Heng, C. K., Malek, S. N. A., Sabaratnam, V., and Kuppusamy, U. R. (2016). Giant oyster mushroom pleurotus giganteus (agaricomycetes) enhances adipocyte differentiation and glucose uptake via activation of pparγ and glucose transporters 1 and 4 in 3t3-l1 cells. *Int. J. Med. Mushrooms*.18(9), 821-831. doi: 10.1615/IntJMedMushrooms.v18.i9.60

Preuss, H. G., Echard, B., Bagchi, D., Perricone, N. V., and Zhuang, C. (2007). Enhanced insulin-hypoglycemic activity in rats consuming a specific glycoprotein extracted from maitake mushroom. *Mol. Cell. Biochem*. 306, 105–113.

Qiao, J., Dou, Z., Xu, J., Wu, F., Meng, G., & Chen, H., et al. (2014). Intervention of Ganoderma Lucidum polysaccharide in combination with metformin on myocardial fibrosis in diabetes mellitus rats. *Chinese Tradit. Herb. Drugs*. 34 (20), 1709–1713.

Rašeta, M., Popović, M., Čapo, I., Stilinović, N., Vukmirović, S., Milošević, B., et al. (2020). Antidiabetic effect of two different Ganoderma species tested in alloxan diabetic rats. *Rsc. Adv.* 10, 10382–10393.

Ratnaningtyas, N. I., Ekowati, N., Sukmawati, D., and Widianti, H. (2019). Chicken drumstick mushroom (Coprinus comatus) ethanol extract exerts a hypoglycaemic effect in the Rattus norvegicus model of diabetes. *Biocatal. Agric. Biotechnol*. 19, 101050.

Ravi, B., Renitta, R. E., Prabha, M. L., Issac, R., and Naidu, S. (2013). Evaluation of antidiabetic potential of oyster mushroom (Pleurotus ostreatus) in alloxan-induced diabetic mice. Immunopharmacol. *Immunotoxicol.* 35, 101–109.

Rony, K. A., Ajith, T. A., and Janardhanan, K. K. (2015). Hypoglycemic and hypolipidemic effects of the cracked-cap medicinal mushroom Phellinus rimosus (Higher Basidiomycetes) in streptozotocin-induced diabetic rats. *Int. J. Med. Mushrooms*, 17(6), 521-31. doi: 10.1615/intjmedmushrooms.v17.i6.30.

Rony, K. A., Ajith, T. A., Mathew, J., and Janardhanan, K. K. (2013). The medicinal cracked-cap polypore mushroom Phellinus rimosus (higher Basidiomycetes) attenuates alloxan-induced hyperglycemia and oxidative stress in rats. *Int. J. Med. Mushrooms*, 15(3), 287-300. doi: 10.1615/intjmedmushr.v15.i3.60.

Rushita, S., Vijayakumar, M., Noorlidah, A., Abdulla, M. A., & Vikineswary, S. (2013). Effect of Pleurotus citrinopileatus on blood glucose, insulin and catalase of streptozotocin-induced type 2 diabetes mellitus rats. J. Anim. Plant. Sci, 23, 1566-1571.

Seedevi, P., Ramu Ganesan, A., Moovendhan, M., Mohan, K., Sivasankar, P., Loganathan, S., et al. (2020). Anti-diabetic activity of crude polysaccharide and rhamnose-enriched polysaccharide from G. lithophila on Streptozotocin (STZ)-induced in Wistar rats. Sci. Rep. 10, 1–12.

Shin, S., Lee, S., Kwon, J., Moon, S., Lee, S., Lee, C.-K., et al. (2009). Cordycepin suppresses expression of diabetes regulating genes by inhibition of lipopolysaccharide-induced inflammation in macrophages. *Immune Netw*. 9, 98–105.

Singh, V., Bedi, G. K., and Shri, R. (2017). In vitro and in vivo antidiabetic evaluation of selected culinary-medicinal mushrooms (Agaricomycetes). Int. J. Med. Mushrooms, 19(1), 17-25. doi: 10.1615/IntJMedMushrooms.v19.i1.20.

Sonawane, H., Arya, S., Ghole, V., Apte, K., Shelke, D., and Chaskar, M. (2020). Hypoglycemic and anticataract activity of crude exopolysaccharides of medicinal mushroom Phellinus badius on streptozotocin-induced diabetic rats and goat eye lenses respectively. Bioact. *Carbohydr. Diet. Fibre* 24, 100241.

Su, C.-H., Lu, T.-M., Lai, M.-N., and Ng, L.-T. (2013). Inhibitory potential of Grifola frondosa bioactive fractions on α-amylase and α-glucosidase for management of hyperglycemia. *Biotechnol. Appl. Biochem*. 60, 446–452.

Teng, J.-F., Lee, C.-H., Hsu, T.-H., and Lo, H.-C. (2018). Potential activities and mechanisms of extracellular polysaccharopeptides from fermented Trametes versicolor on regulating glucose homeostasis in insulin-resistant HepG2 cells. *Plos One* 13, e0201131.

Tian, Y., Nichols, R. G., Roy, P., Gui, W., Smith, P. B., Zhang, J., et al. (2018). Prebiotic effects of white button mushroom (Agaricus bisporus) feeding on succinate and intestinal gluconeogenesis in C57BL/6 mice. *J. Funct. Foods* 45, 223–232.

Tie, L., Yang, H.-Q., An, Y., Liu, S.-Q., Han, J., Xu, Y., et al. (2012). Ganoderma lucidum polysaccharide accelerates refractory wound healing by inhibition of mitochondrial oxidative stress in type 1 diabetes. *Cell. Physiol. Biochem*. 29, 583–594.

Uribarri, J., Woodruff, S., Goodman, S., Cai, W., Chen, X., Pyzik, R., et al. (2010). Advanced glycation end products in foods and a practical guide to their reduction in the diet. *J. Am. Diet. Assoc*. 110, 911–916.

Vincent, M., Philippe, E., Everard, A., Kassis, N., Rouch, C., Denom, J., et al. (2013). Dietary supplementation with Agaricus blazei murill extract prevents diet-induced obesity and insulin resistance in rats. *Obesity* 21, 553–561.

Vyacheslav, B., Alexey, B., Aliaksandr, A., Valentina, M., Tatyana, P., Aliaksandar, K., et al. (2019). Polysaccharides of mushroom phallus impudicus mycelium: immunomodulating and wound healing properties. *Modern Food Sci. Tech*. 35 (9), 30–37..

Wahab, N. A. A., Abdullah, N., and Aminudin, N. (2014). Characterisation of potential antidiabetic-related proteins from Pleurotus pulmonarius (Fr.) Quél.(grey oyster mushroom) by MALDI-TOF/TOF mass spectrometry. *BioMed Res. Int*. 2014, 131607. doi: 10.1155/2014/131607

Wang, C. R., Ng, T. B., Li, L., Fang, J. C., Jiang, Y., Wen, T. Y., et al. (2011). Isolation of a polysaccharide with antiproliferative, hypoglycemic, antioxidant and HIV-1 reverse transcriptase inhibitory activities from the fruiting bodies of the abalone mushroom Pleurotus abalonus. *J. Pharm. Pharmacol.* 63, 825–832.

Wang, C., Gao, X., Santhanam, R. K., Chen, Z., Chen, Y., Xu, L., et al. (2018a). Effects of polysaccharides from Inonotus obliquus and its chromium (III) complex on advanced glycation end-products formation, α-amylase, α-glucosidase activity and H2O2-induced oxidative damage in hepatic L02 cells. *Food Chem. Toxicol*. 116, 335–345.

Wang, J. C., Hu, S. H., Wang, J. T., Chen, K. S., and Chia, Y. C. (2005). Hypoglycemic effect of extract of Hericium erinaceus. *J. Sci. Food Agric*. 85, 641–646.

Wang, J., Teng, L., Liu, Y., Hu, W., Chen, W., Hu, X., et al. (2016). Studies on the antidiabetic and antinephritic activities of Paecilomyces hepiali water extract in diet-streptozotocin-induced diabetic Sprague Dawley rats. *J. Diabetes Res*. 2016, 4368380. doi: 10.1155/2016/4368380.

Wang, J., Wang, C., Li, S., Li, W., Yuan, G., Pan, Y., et al. (2017). Anti-diabetic effects of Inonotus obliquus polysaccharides in streptozotocin-induced type 2 diabetic mice and potential mechanism via PI3K-Akt signal pathway. *Biomed. Pharmacother*. 95, 1669–1677.

Wang, J., Wu, C., Chen, Y., Chen, C., Hu, S., and Chang, S. (2014). Antihyperglycemic activity of exopolysaccharide produced by mushroom Pleurotus ferulae with submerged liquid culture on streptozotocin-induced diabetic rats. *J. Food Nutr. Res*. 2, 419–424.

Wu, T.-R., Lin, C.-S., Chang, C.-J., Lin, T.-L., Martel, J., Ko, Y.-F., et al. (2019). Gut commensal Parabacteroides goldsteinii plays a predominant role in the anti-obesity effects of polysaccharides isolated from Hirsutella sinensis. *Gut* 68, 248–262.

Wu, W.-T., Hsu, T.-H., Lee, C.-H., and Lo, H.-C. (2020). Fruiting bodies of Chinese caterpillar mushroom, Ophiocordyceps sinensis (Ascomycetes) alleviate diabetes-associated oxidative stress. *Int. J. Med. Mushrooms* 22(1), 15-29. doi: 10.1615/IntJMedMushrooms.2019033275.

Xian, H., Che, H., Qin, Y., Yang, F., Meng, S., Li, X., et al. (2018). Coriolus versicolor aqueous extract ameliorates insulin resistance with PI3K/Akt and p38 MAPK signaling pathways involved in diabetic skeletal muscle. *Phytother. Res*. 32, 551–560.

Xiang, H., Sun-Waterhouse, D., and Cui, C. (2021). Hypoglycemic polysaccharides from Auricularia auricula and Auricularia polytricha inhibit oxidative stress, NF-κB signaling and proinflammatory cytokine production in streptozotocin-induced diabetic mice. *Food Sci.* *Hum. Wellness* 10, 87–93.

Xiao, C., Wu, Q., Xie, Y., Zhang, J., and Tan, J. (2015). Hypoglycemic effects of Grifola frondosa (Maitake) polysaccharides F2 and F3 through improvement of insulin resistance in diabetic rats. *Food Funct.* 6, 3567–3575.

Xiao, C., Wu, Q., Zhang, J., Xie, Y., Cai, W., and Tan, J. (2017). Antidiabetic activity of Ganoderma lucidum polysaccharides F31 down-regulated hepatic glucose regulatory enzymes in diabetic mice. *J. Ethnopharmacol*. 196, 47–57.

Xiao, C., Wu, Q.-P., Cai, W., Tan, J.-B., Yang, X.-B., and Zhang, J.-M. (2012). Hypoglycemic effects of Ganoderma lucidum polysaccharides in type 2 diabetic mice. *Arch. Pharm. Res.* 35, 1793–1801

Xiong, M., Huang, Y., Liu, Y., Huang, M., Song, G., Ming, Q., et al. (2018). Antidiabetic activity of ergosterol from Pleurotus ostreatus in KK-Ay mice with spontaneous type 2 diabetes mellitus. *Mol. Nutr. Food Res*. 62, 1700444.

Xue, J., Tong, S., Wang, Z., and Liu, P. (2018). Chemical characterization and hypoglycaemic activities in vitro of two polysaccharides from Inonotus obliquus by submerged culture. *Molecules* 23, 3261.

Yamac, M., Kanbak, G., Zeytinoglu, M., Bayramoglu, G., Senturk, H., and Uyanoglu, M. (2008). Hypoglycemic effect of Lentinus strigosus (Schwein.) Fr. crude exopolysaccharide in streptozotocin-induced diabetic rats. J*. Med. Food* 11, 513–517.

Yamac, M., Zeytinoglu, M., Kanbak, G., Bayramoglu, G., and Senturk, H. (2009). Hypoglycemic effect of crude exopolysaccharides produced by Cerrena unicolor, Coprinus comatus, and Lenzites betulina isolates in streptozotocin-induced diabetic rats. *Pharm. Biol*. 47, 168–174.

Yamaç, M., Zeytinoglu, M., Senturk, H., Kartkaya, K., Kanbak, G., Bayramoglu, G., et al. (2016). Effects of black hoof medicinal mushroom, Phellinus linteus (Agaricomycetes), polysaccharide extract in streptozotocin-induced diabetic rats. *Int. J. Med. Mushrooms,* 18(4), 301-11. doi: 10.1615/IntJMedMushrooms.v18.i4.30.

Yang CC, Chen SN, Lu CL, Chen S, Lai KC, et al. (2014) Effect of Mushroom Beta Glucan (MBG) on Immune and Haemocyte Response in Pacific White Shrimp (Litopenaeus vannamei). J Aquac Res Development 5: 275 doi:10.4172/2155-9546.1000275

Yang, B.-K., Kim, D.-H., Jeong, S.-C., Das, S., Choi, Y.-S., Shin, J.-S., et al. (2002). Hypoglycemic effect of a Lentinus edodes exo-polymer producedfrom a submerged mycelial culture. *Biosci. Biotechnol. Biochem.* 66, 937–942.

Yoo, O.-K., Son, J.-H., and Lee, D.-H. (2005). 4-acetoxyscirpendiol of Paecilomyces tenuipes inhibits Na+/D-glucose cotransporter expressed in Xenopus laevis oocytes. *BMB Rep*. 38, 211–217.

Yu, S.-H., Dubey, N. K., Li, W.-S., Liu, M.-C., Chiang, H.-S., Leu, S.-J., et al. (2016). Cordyceps militaris treatment preserves renal function in type 2 diabetic nephropathy mice. *PLoS One* 11, e0166342.

Zhang, A., Deng, J., Yu, S., Zhang, F., Linhardt, R. J., and Sun, P. (2018). Purification and structural elucidation of a water-soluble polysaccharide from the fruiting bodies of the Grifola frondosa. *Int. J. Biol. Macromol*. 115, 221–226.

Zhang, A., Zhang, J., Tang, Q., Jia, W., Yang, Y., Liu, Y., et al. (2006). Structural elucidation of a novel fucogalactan that contains 3-O-methyl rhamnose isolated from the fruiting bodies of the fungus, Hericium erinaceus. *Carbohydr. Res*. 341, 645–649.

Zhang, C., Li, J., Hu, C., Wang, J., Zhang, J., Ren, Z., et al. (2017). Antihyperglycaemic and organic protective effects on pancreas, liver and kidney by polysaccharides from Hericium erinaceus SG-02 in streptozotocin-induced diabetic mice. *Sci. Rep*. 7, 1–13.

Zhang, C., Zhang, L., Liu, H., Zhang, J., Hu, C., and Jia, L. (2018b). Antioxidation, anti-hyperglycaemia and renoprotective effects of extracellular polysaccharides from Pleurotus eryngii SI-04. *Int. J. Biol. Macromol*. 111, 219–228.

Zhang, H.-N., He, J.-H., Yuan, L., and Lin, Z.-B. (2003). In vitro and in vivo protective effect of Ganoderma lucidum polysaccharides on alloxan-induced pancreatic islets damage. *Life Sci.* 73, 2307–2319

Zhang, J., Meng, G., Zhang, C., Lin, L., Xu, N., Liu, M., et al. (2015). The antioxidative effects of acidic-, alkalic-, and enzymatic-extractable mycelium zinc polysaccharides by Pleurotus djamor on liver and kidney of streptozocin-induced diabetic mice. *BMC Complement. Altern. Med.* 15, 1–12.

Zhang, Y., Hu, T., Zhou, H., Zhang, Y., Jin, G., and Yang, Y. (2016c). Antidiabetic effect of polysaccharides from Pleurotus ostreatus in streptozotocin-induced diabetic rats. *Int. J. Biol. Macromol*. 83, 126–132.

Zhang, Z. C., Lian, B., Huang, D. M., and Cui, F. J. (2009). Compare activities on regulating lipid-metabolism and reducing oxidative stress of diabetic rats of Tremella aurantialba broth’s extract (TBE) with its mycelia polysaccharides (TMP). *J. Food Sci*. 74, H15–H21.

Zhao, C., Liao, Z., Wu, X., Liu, Y., Liu, X., Lin, Z., et al. (2014). Isolation, purification, and structural features of a polysaccharide from Phellinus linteus and its hypoglycemic effect in alloxan-induced diabetic mice. *J. Food Sci*. 79, H1002–H1010.

Zhao, H., Lai, Q., Zhang, J., Huang, C., & Jia, L. (2018). Antioxidant and hypoglycemic effects of acidic-extractable polysaccharides from Cordyceps militaris on type 2 diabetes mice. Oxid. Med. Cell. Longev. 2018, 9150807. doi: 10.1155/2018/9150807. eCollection 2018.

Zheng, J., Yang, B., Yu, Y., Chen, Q., Huang, T., and Li, D. (2012). Ganoderma lucidum polysaccharides exert anti-hyperglycemic effect on streptozotocin-induced diabetic rats through affecting β-cells. *Comb. Chem. High Throughput Screen*. 15, 542–550.

Zheng, Y., Bai, L., Zhou, Y., Tong, R., Zeng, M., Li, X., et al. (2019). Polysaccharides from Chinese herbal medicine for anti-diabetes recent advances. *Int. J. Biol. Macromol*. 121, 1240–1253.

Zheng, Y., Fan, J., Chen, H.W., & Liu, E.Q. (2019). Trametes orientalis polysaccharide alleviates PM 2.5-induced lung injury in mice through its antioxidant and anti-inflammatory activities. *Food & function*, 10(12), 8005-8015.

Zhou, S., Liu, Y., Yang, Y., Tang, Q., and Zhang, J. (2015). Hypoglycemic activity of polysaccharide from fruiting bodies of the shaggy ink cap medicinal mushroom, Coprinus comatus (higher Basidiomycetes), on mice induced by alloxan and its potential mechanism. *Int. J. Med. Mushrooms,* 17(10), 957-64. doi: 10.1615/intjmedmushrooms.v17.i10.50.
